# Pyrimidine-based NNRTIs in HIV therapy: advances in synthesis, potency and structure–activity relationships

**DOI:** 10.1039/d6ra04544h

**Published:** 2026-08-18

**Authors:** Hammad Ali, Muhammad Aown Hashmi, Nimra Ajmal, Muhammad Saad Goraija, Nasir Rasool, Umme Habibah Siddiqua, Ayesha Malik

**Affiliations:** a Department of Chemistry, Government College University Faisalabad Faisalabad 38000 Pakistan drayeshamalik@gcuf.edu.pk; b Department of Chemistry, University of Jhang Jhang Pakistan u.habibah5@gmail.com

## Abstract

Acquired Immunodeficiency Syndrome (AIDS) caused by human immunodeficiency virus (HIV), as a global health concern, needs to be addressed by the development of new antiretroviral drugs. Pyrimidine is a valued scaffold for the treatment of HIV/AIDS due to its resemblance to the nucleotide base pairs of DNA and RNA. Pyrimidine-based compounds, which have turned out to be the most important compounds with lower cytotoxicity and enhanced antiviral activity, can serve as leads to synthesize new antiretroviral drugs. This review covers the recent advances in the synthesis of pyrimidine-based antiretroviral drugs showing better activity against wild-type and resistant HIV strains, with a key emphasis on their structure–activity relationships. This review aims to assist future research endeavors in designing potent pyrimidine-based non-nucleoside reverse transcriptase inhibitors for resistant HIV strains to overcome the current problems by summarizing compounds reported from 2018 onwards. Based on our analysis, compounds with a pyrimidine core (31, 52c, 56c, 66a, 72b, 82a, 85a, 132b, 185a and 191c) tend to be highly potent against various HIV targets and have the potential to overcome antiviral resistance. This review highlights the significance of this pharmacophoric fragment for developing effective and innovative antiviral drugs.

## Abstract 2

Acquired immunodeficiency syndrome (AIDS) caused by human immunodeficiency virus (HIV), as a global health concern, needs to be addressed by the development of new antiretroviral drugs

## Graphical abstract

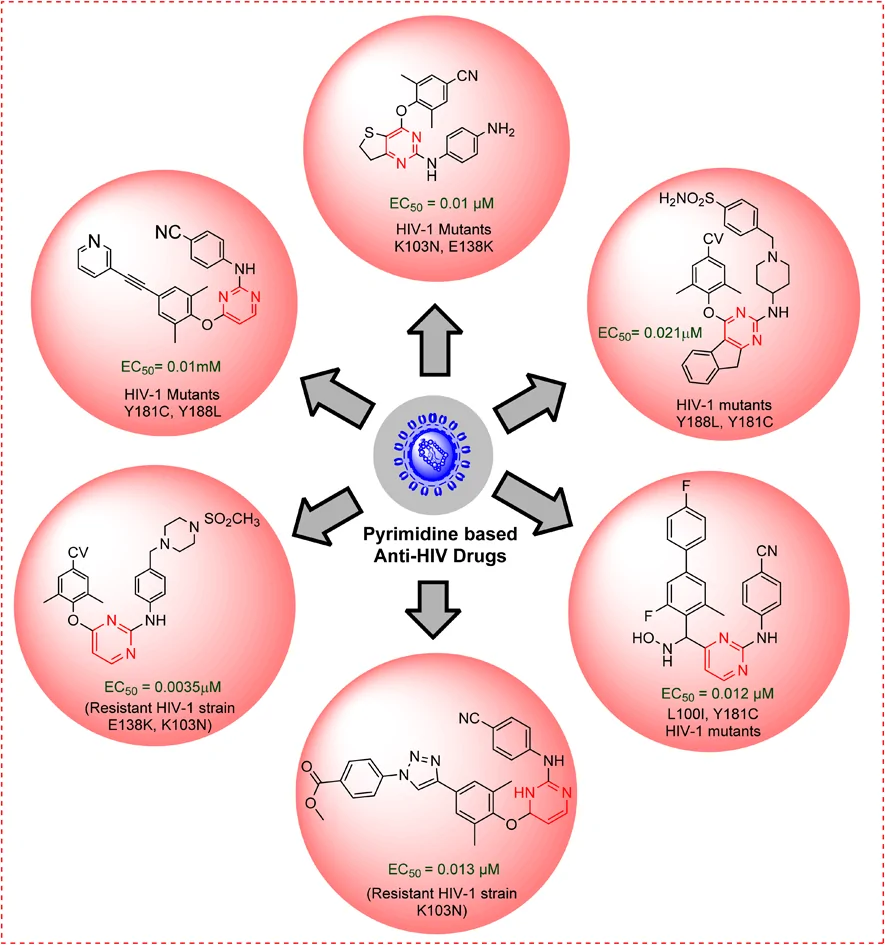

## 1. Introduction

**Fig. 1:**
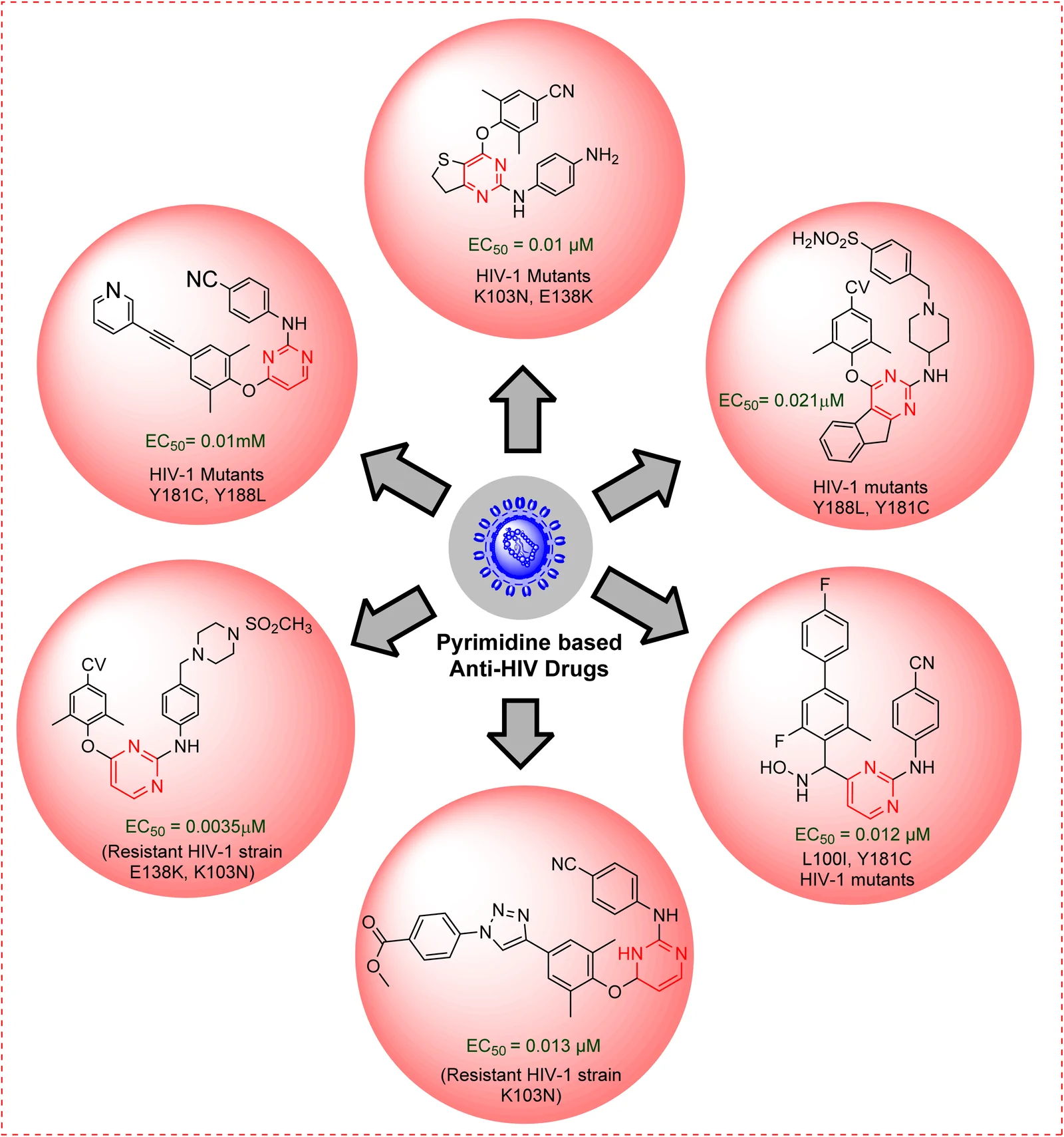
Graphical abstract.

Human immunodeficiency virus-induced AIDS continues to be one of the deadliest diseases worldwide.^1–3^ By the end of 2023, nearly 39 900 000 people were living with HIV, with 65% in the WHO African region. Tragically, about 0.63 million lives were lost to HIV-related causes as per the statistical data from the WHO.^4,5^ The first confirmed case of HIV infection was seen in a preserved blood sample of a male from Kinshasa, Central Africa, in 1959. There were a few early HIV medicines, but the majority of patients died even after the administration of these medications (Fig. 1).

However, more than thirty years of research have yielded a lot of understanding regarding the natural history of HIV, which enabled the development of successful antiretroviral therapy (ART).^6^

Among the two classes of HIV strains, namely, HIV-1 and HIV-2,^7^ HIV-1 is the most pathogenic one.^8^ HIV is a member of Retroviridae (retrovirus) and is transmitted through blood, semen, and vaginal fluid and from mother to child.^9^ The immune system of the host is compromised by HIV-1 as it attacks the CD4 + T lymphocytes, which are important for fighting off infections.^10^ HIV-1 replicates by converting its viral RNA into complementary DNA (cDNA)^11^ by an enzyme called reverse transcriptase (RT) through a reverse transcription process.^12,13^

Antiretroviral drugs (ARV) have been developed to combat HIV-1,^14^ which have managed to transform it into a manageable chronic condition from a fatal disease.^15,16^ Among the various classes of antiretroviral drugs, reverse transcriptase inhibitors are very important,^17^ including non-nucleoside reverse transcriptase inhibitors^18^ and nucleoside reverse transcriptase inhibitors.^19^ Their common target is reverse transcriptase.^20^

NNRTIs, because of their distinctive efficacy, minimal toxicity, and high specificity,^21,22^ have established a firm position in antiretroviral therapy (ART) to treat HIV-1.^23,24^ The FDA has approved 6 NNRTIs, out of which 3 are pyrimidine derivatives.^25,26^

**Fig. 2:**
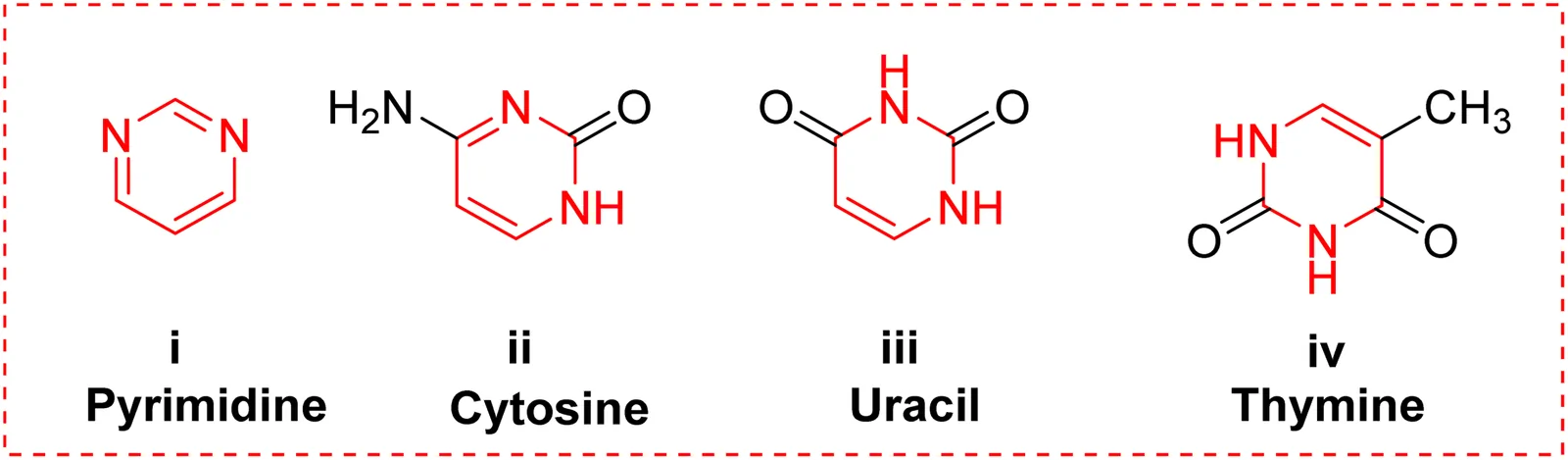
Structures of pyrimidine, cytosine, uracil and thymine.

Pyrimidine is a colorless, nitrogen-containing heterocycle, which is a fundamental component of DNA [thymine (iv) and cytosine (ii)] and RNA [cytosine (ii) and uracil (iii)] (Fig. 2)^27,28^ and a very important heterocycle in medicinal chemistry.^29,30^ It is also a building block of many natural compounds such as barbiturates,^31^ which are a class of sedatives and hypnotic medications,^32^ and thiamine (vitamin B1).^33^

The pyrimidine scaffold is valued for its synthetic accessibility and has diverse therapeutic applications, including anticancer,^34,35^ antibacterial,^36,37^ antihistamine,^38,39^ anti-inflammatory,^40,41^ anticonvulsant,^42,43^ antimalarial,^43,44^ antimicrobial,^45,46^ antihypertensive,^47,48^ antifungal,^49,50^ analgesic,^51,52^ antiviral,^53,54^ and antioxidant^55^ applications.^56^

**Fig. 3:**
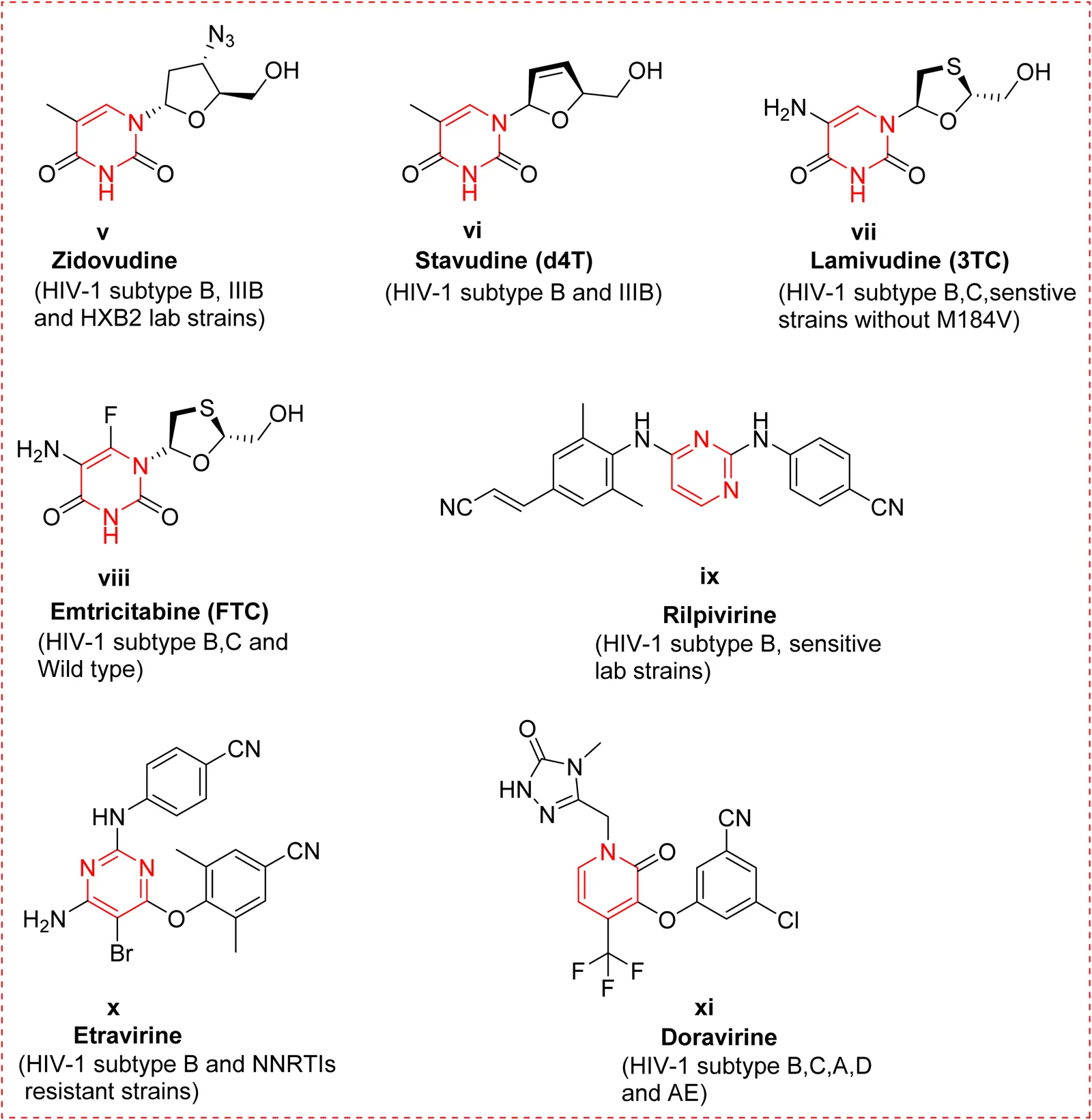
FDA-approved pyrimidine-based anti-HIV-1 drugs.

Pyrimidine derivatives, especially diarylpyrimidines, show excellent antiviral activity against HIV-1.^22,57,58^ To date, seven pyrimidine-based drugs against HIV have been approved by the FDA, including four NRTIs (zidovudine (v),^59,60^ stavudine (vi),^61,62^ lamivudine (vii),^63,64^ and emtricitabine (viii)^65^) and three NNRTIs (rilpivirine (ix),^66,67^ etravirine (x),^68,69^ and doravirine (xi)^70^) (Fig. 3).

### 1.1. Structural basis of resistance and evolution of NNRTIs

Nevirapine, delavirdine and efavirenz are first-generation NNRTIs introduced in the 1990s and have been used as drugs in antiretroviral therapy; these three drugs share cross-resistance and are prone to resistance development because only one mutation (K103N) at a single position is sufficient to confer resistance to all three agents.^71^ This fragility led to the development of “conformational” or “flexible” second-generation NNRTIs (etravirine and rilpivirine), which were more conformationally flexible than the original structurally rigid scaffolds and were able to bind to an expanded array of K103N-containing NNRTI-resistant viral variants; however, the resulting benefits were less as more resistance mutations were acquired.^72^ Third-generation doravirine was developed with a more controlled, rational approach: it was designed to directly address known issues with previous-generation NNRTIs, such as CNS toxicity associated with efavirenz and cardiac ion-channel effects associated with rilpivirine, while addressing resistance and drug-interaction concerns. Second- and third-generation NNRTIs were specifically developed to be potent against the K103N, Y181C and G190A resistance mutations that first-generation NNRTIs were vulnerable to.^73^ Overall, the three generations clearly follow each other in a logical progression from serendipity-driven drugs to increasingly resistance-driven to more structure-driven drugs.^74^

Consistent with the number of available crystal structures of RT, the mutation of NRTI resistance is located further away from the binding site, and for NNRTIs, the mutations are more concentrated. The clustering indicates that there may be a direct stereochemical basis for NNRTI resistance mechanisms, as is often the case, *e.g.*, loss of key ring stacking interactions with inhibitors through Tyr181 and Tyr188 mutations. However, some indirect resistance mechanisms have also been seen, such as V108I and K103N.^75^ The Y181C and Y188C mutations were found to validate the absence of aromatic ring stacking in first-generation inhibitors (FGIs), like nevirapine and Tyr181/Tyr188. The loss of nevirapine's binding potency by 100-fold for Y181C can be attributed, to a significant extent, to the loss of aromatic ring stacking.^76^ K103N creates an energy barrier for the NNRTI, thus providing some reduction in the potency. The cross-resistance between K103N and several NNRTIs may also be related to the mechanism of the stabilization of the apoenzyme.^77^ The loss of potency by 10-fold is attributed to the presence of the V108I mutation at the back of Tyr188, which is part of the active site side chain.^78^ The K101E mutation distorts the network of water molecules that surrounds the inhibitor on one side of the NNRTI pocket, resulting in a reduction in potency, most likely as a result of NNRTI/Tyr181 interaction changes.^79^ The aim of the structure-based drug design study was to minimize interactions of the phenyl ring of MKC-442 with Tyr181 and Tyr188 and maximize interactions with the conserved Trp229. Adding a di-*meta* substituent caused increased contacts with Trp229, but reduced contacts with Tyr181 and Tyr188. Thus, the obtained GCA-186 compound was highly active against the Y181C mutant RT. The effect of the TMC-125-type inhibitors was shown to be very good, and this was explained by the ‘wiggling’ and ‘jiggling’ of the inhibitor. Capravirine (S-1153) has been shown to form three main-chain hydrogen bonds. Side-chain interactions are less likely to be disrupted by mutations than by such interactions.^80^

The non-nucleoside inhibitor binding pocket (NNIBP) of HIV-1 RT is mostly hydrophobic, with residues L100, V106, T107, V108, Y181, Y188, G190, F227, W229, L234 and Y318 playing a significant role in making up the non-nucleoside inhibitor-binding pocket (NNIBP), while others, such as P236, K101, and K103, do not.^81^ This type of NNRTIs interacts with key residues, such as Y181, Y188, K103, F227, E138, V106 and Y318, which are repeatedly found to be involved in drug binding and resistance.^80,82^ The mutations at these positions affect the conformational flexibility of the pocket and its ability to bind inhibitors, and therefore, they have been identified as important for the mechanistic principles of NNRTI design.^83^ The mechanism of binding is explained by the structural framework of pyrimidine derivatives, which are involved in hydrophobic contacts and hydrogen-bonding interactions in the NNIBP, explaining their potency and resistance profiles.^25^

Etravirine^84^ and rilpivirine^85^ are second-generation NNRTIs^86^ and are highly active against HIV-1 WT strains.^87,88^ However, because viruses change their genetic material quite often, new mutations, especially single mutations like K103N when combined with Y181C and double mutations like F227L when combined with V106A, show strong cross-resistance to rilpivirine and etravirine, thus leading to a reduction in drug effectiveness.^89,90^

Although more than 50 structurally diverse classes of NNRTIs have been discovered in the past few decades, pyrimidine-based compounds (such as etravirine (ETR) and rilpivirine (RPV), which were approved in 2008 and 2011, respectively) continue to be a major focus in current optimization efforts. Newer derivatives are still being added to the medicinal chemistry literature.^91^

**Fig. 4:**
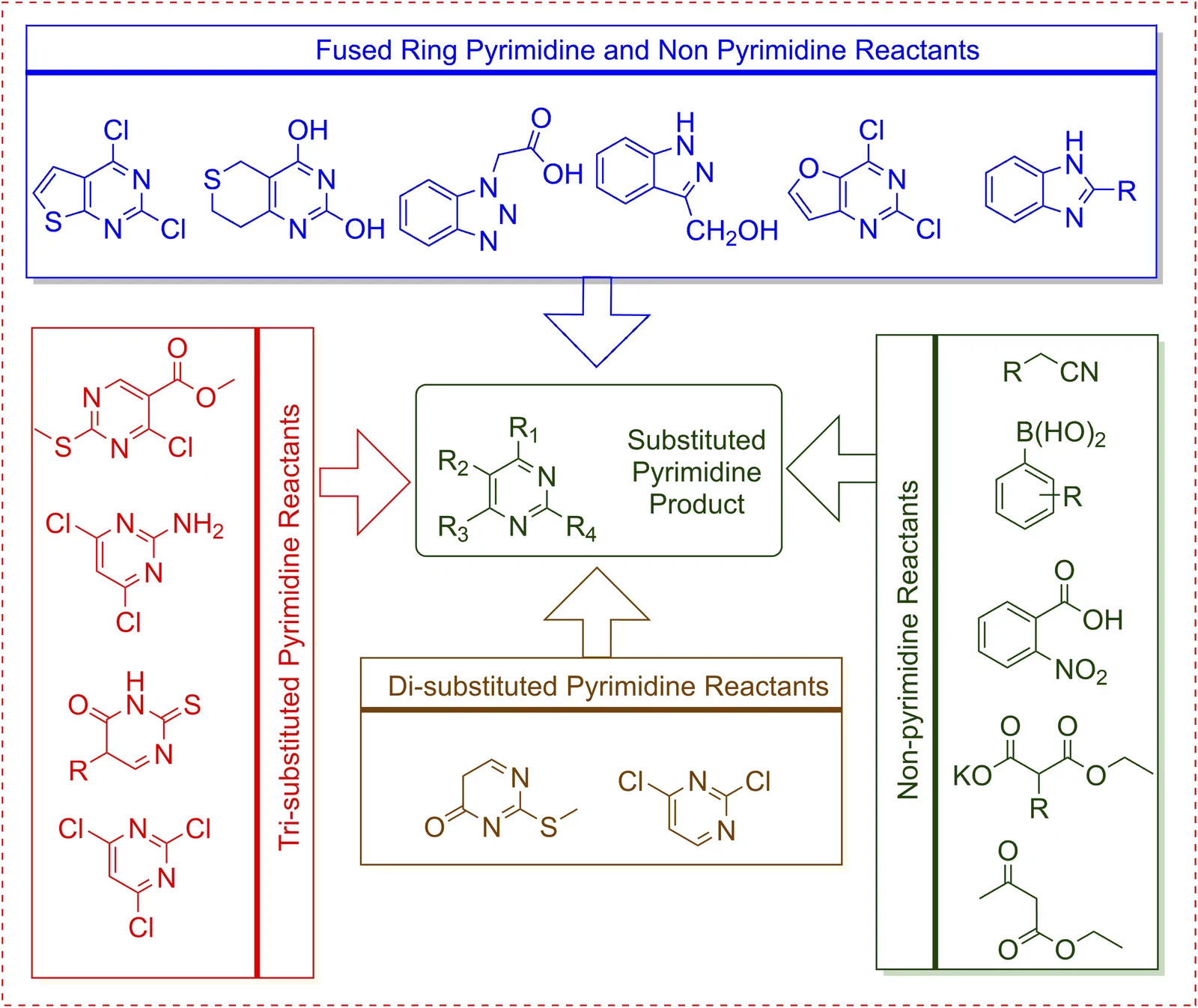
Synthetic routes for pyrimidines from non-pyrimidine moieties, tri-substituted pyrimidines and fused ring pyrimidines.

Thus, we still need new DAPY NNRTIs to solve this problem.^92^ The latest developments in the synthesis of novel pyrimidine-based NNRTIs showing better potency against double and single mutations, along with their structure–activity relationships, as documented in the preceding years, are combined in this review. This review discusses the synthesis of reported pyrimidine derivatives that can serve as lead compounds in the development of new drugs against HIV-1 with better resistance profiles compared to commercially available drugs (Fig. 4).

This review encompasses the literature from 2018 to 2026 from various journals. Studies were selected by evaluating if they involved HIV-1 non-nucleoside reverse transcriptase inhibitors (NNRTIs), their structural scaffolds and related resistance mechanisms. This criterion helped to make this review clear and reproducible and keep the time-span statements consistent throughout.

## 2. Synthesis of potent anti-HIV pyrimidines

### 2.1. Piperazine-substituted pyrimidine derivatives

**Scheme 1:**
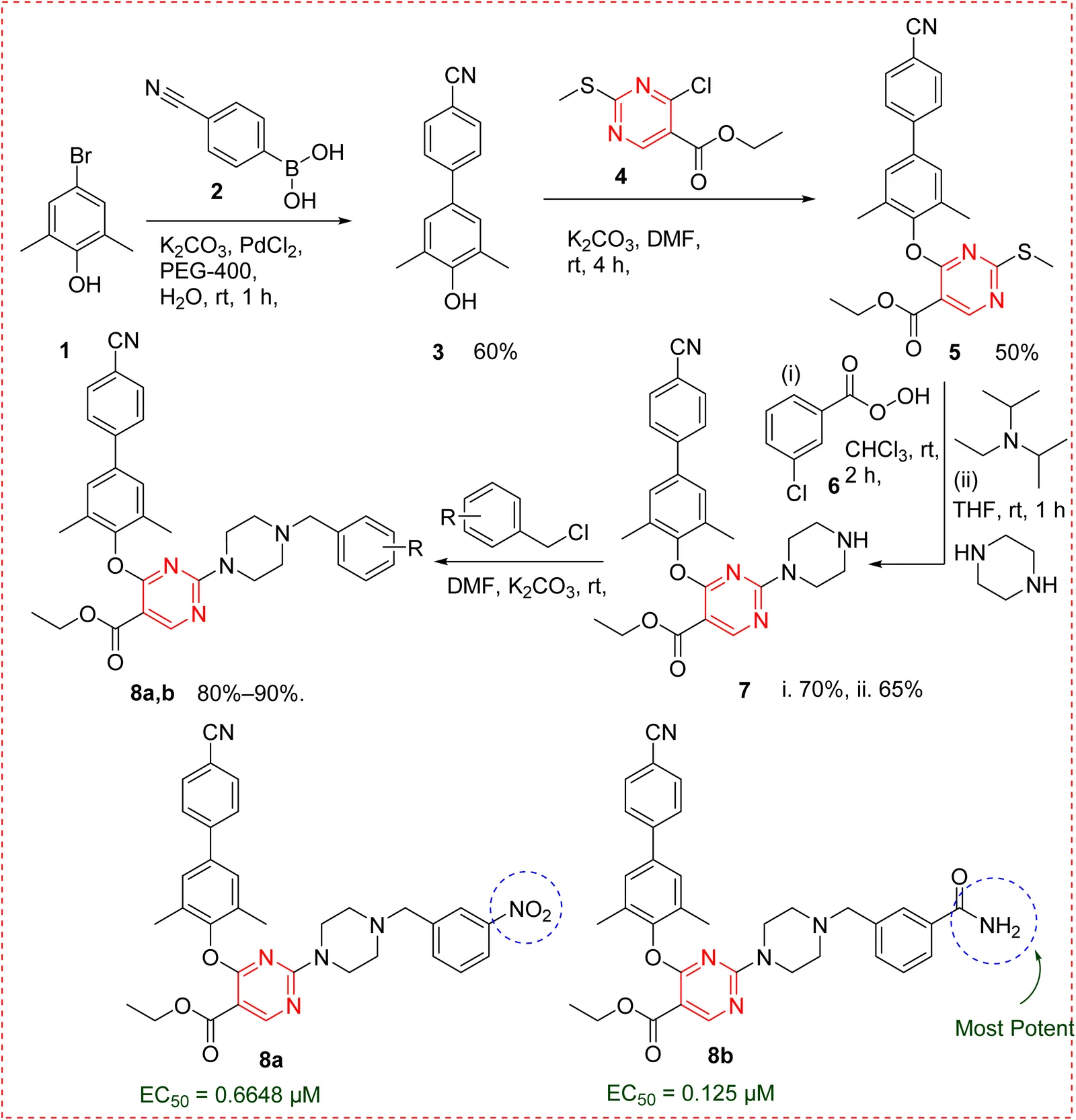
Synthesis of pyrimidine derivatives 8a, b.

Jin *et al.* synthesized novel pyrimidine derivatives (8a, b) that were active as non-nucleoside reverse transcriptase inhibitors (NNRTIs) against HIV-1 (Scheme 1). The Suzuki cross-coupling of commercially available 4-bromo-2,6-dimethylphenol (1) with (4-cyanophenyl)boronic acid (2) in the presence of K_2_CO_3_ and PdCl_2_ led to intermediate 3. The next step involved intermediate 3 reacting with ethyl 4-chloro-2-(methylthio)pyrimidine-5-carboxylate (4) to form a pyrimidine-coupled intermediate (5), which was then oxidized with *m*-CPBA (6) and aminated with piperazine to produce compound 7. The target compounds (8a, b) were obtained by the nucleophilic substitution of 7 with substituted benzyl chlorides in the presence of K_2_CO_3_ in DMF at room temperature. Structure–activity relationship (SAR) analysis showed that the introduction of an *N*-substituted piperazine ring improved the flexibility of the molecule but caused a drop in solubility, which has an impact on the binding to the reverse transcriptase pocket; however, polar 3-carbamoyl substitutions resulted in better antiviral activity than nitro group substitution. Compound 8b was the most active against the wild-type HIV-1 IIIB strain (EC_50_ = 0.125 µM) but was inactive against the double-mutant RES056 with K103N and Y181C mutations.^93^

### 2.2. Thiouracil derivatives

**Scheme 2:**
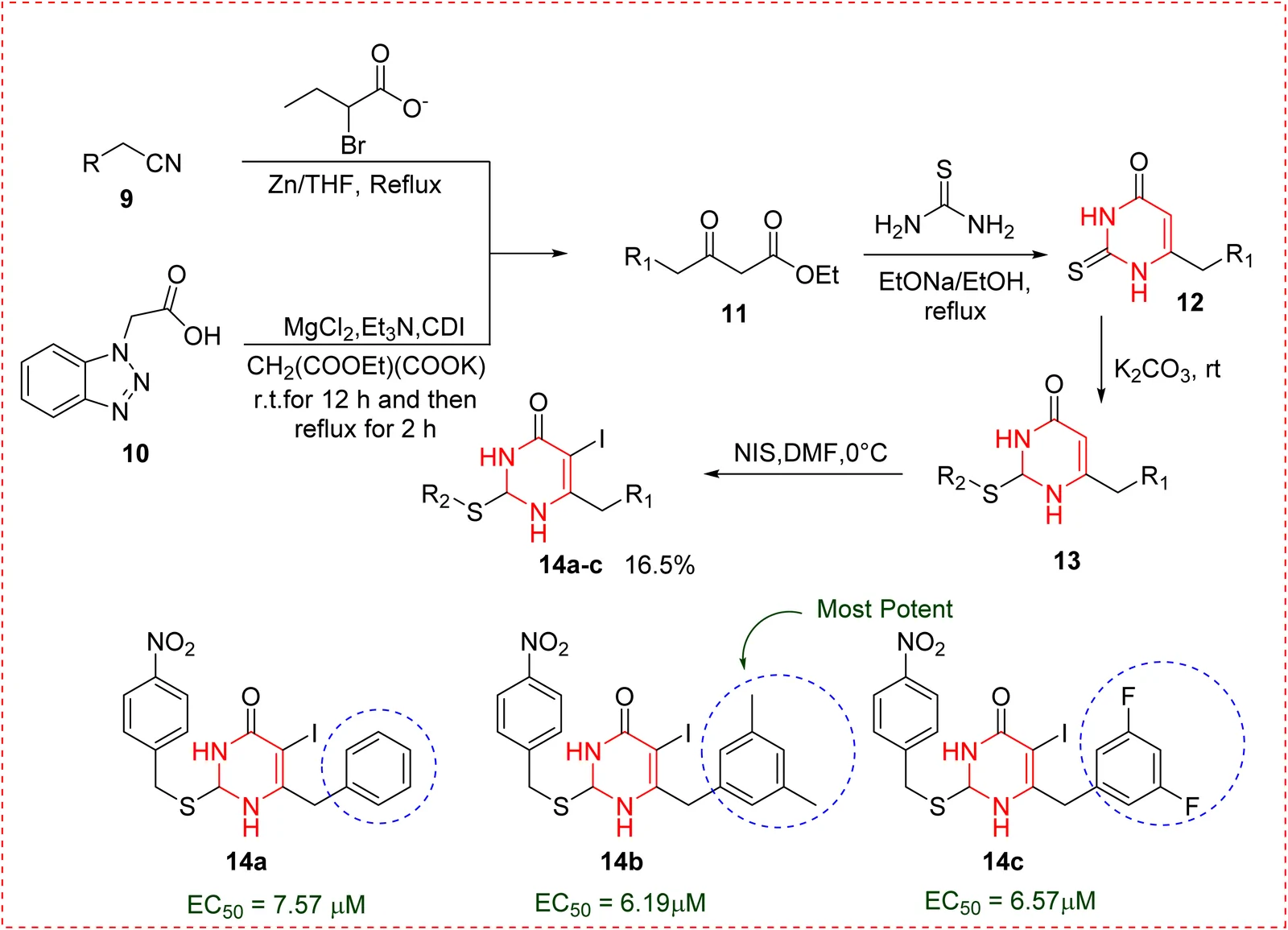
Synthesis of pyrimidine derivatives 14a–c.

Wang *et al.* synthesized 14a–c, which were tested as the inhibitors of hepatitis B virus (HBV) polymerase (Scheme 2). The preparation of B-ketoesters (11) *via* synthesis from benzyl cyanides (9) or from benzo[*d*]triazol-1-yl acetic acid (10) was used to start the synthesis. The condensation of 11 with thiourea in the presence of EtONa/EtOH under reflux gave 6-aryl thiouracils (12). The substitution of the alcohol (12) with alkyl halides in the presence of K_2_CO_3_ gave intermediate 13. The target 2-aryl thio-5-iodopyrimidines (14a–c) were obtained by subsequent iodination at the C-5 position with *N*-iodosuccinimide (NIS) in DMF at 0 °C. Moderate yields, instability of the isolated polymerase, and the presence of impurities (predominantly formed by side-chain substitutions and incomplete iodination) were the drawbacks of this synthetic route to 14a–c. The method was suitable for small-scale assays, moderately cost-effective and considered to be safer than radioisotopic methods in that it used non-radioisotopic ELISA assays, but the environmental safety of the halogen intermediate remained a concern. The mutants that were especially resistant to nucleosides occurred in the reverse transcriptase region, indicating the need for mutation-proof nucleosides with greater potency and resistance profiles. Compound 14b demonstrated a high degree of potency against DNA replication in HepG2.2.15 cell lines, with an EC_50_ value of 6.19 µM against K103N and Y181C mutants.^94^

### 2.3. Tetrasubstituted pyrimidines

**Scheme 3:**
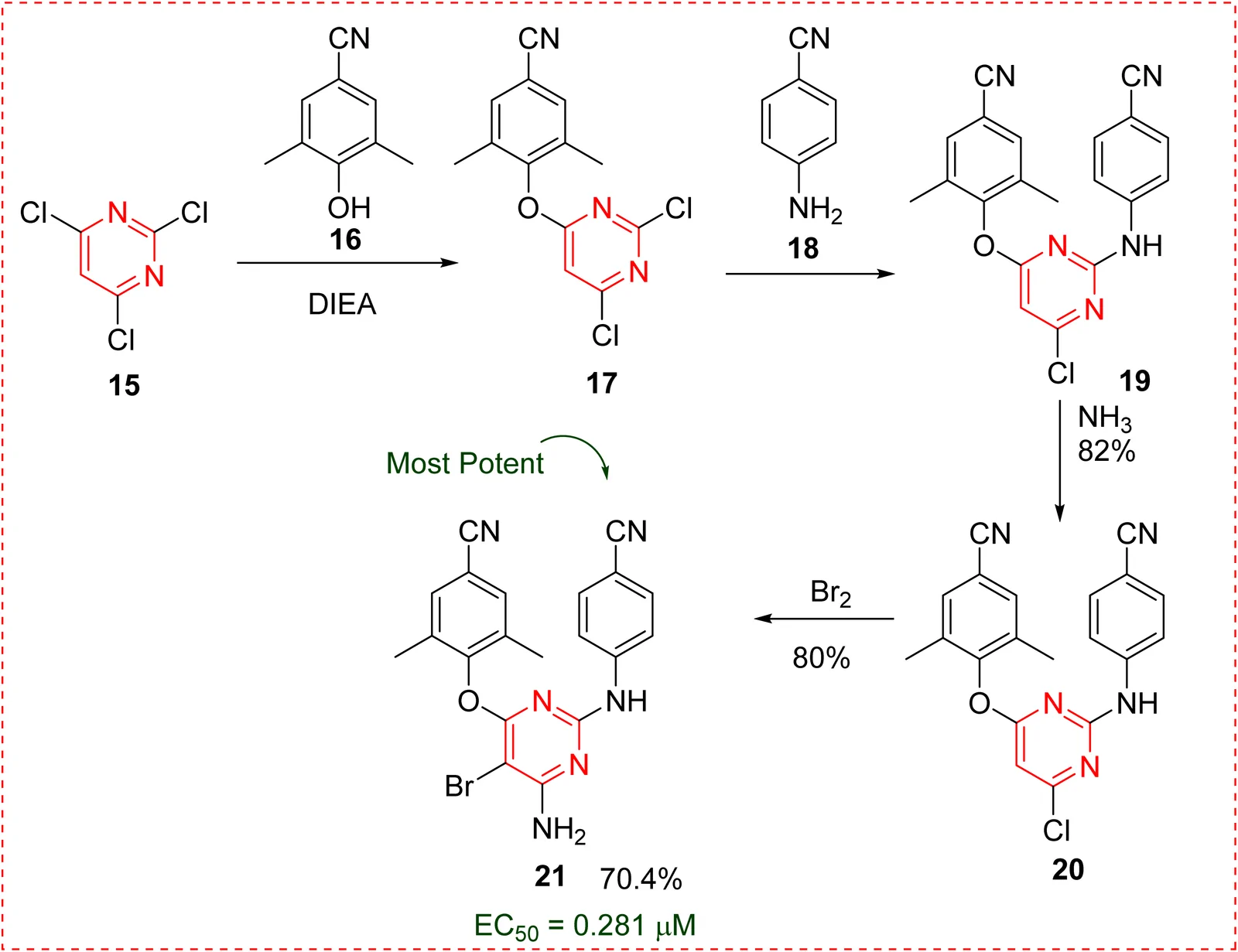
Synthesis of pyrimidine derivative 21.

Feng *et al.* used a microwave-promoted synthesis to prepare an NNRTI named etravirine, which targets the reverse transcriptase of HIV-1 to inhibit the viral replication of drug-resistant variants. The synthesis was started by substituting 2,4,6-trichloropyrimidine (15) with 4-hydroxy-3,5-dimethylbenzonitrile (19) and DIEA to give intermediate 17. In the following step, intermediate 17 reacted with 4-aminobenzonitrile (18) to give intermediate 19. Compound 19 was then aminated using microwave heating to produce compound 20 in 15 minutes. The final step involved the bromination of 20 using bromine, which gave the desired compound 21 with an EC_50_ of 0.281 µM against K103N and Y181C mutants (Scheme 3). This was only performed at the laboratory scale and not scaled up to the industrial scale. The reactions generally afforded high yields, and the final product was purified to >95% purity by HPLC with low content of impurities. The route was very promising considering the conditions required, but the use of organic solvents, such as ethanol and DMF, posed environmental concerns. The reagents used were readily available, but multiple purification steps increased the cost. Although the study was not concerned with structural variations, it reported that compound 21 (etravirine) is a very potent second-generation compound with a high drug-resistant HIV-1 genetic barrier due to its optimized electronic and steric balance.^95^

### 2.4. Thiophene[3,2-*d*]pyrimidines

#### 2.4.1. Thieno[3,2-*d*]pyrimidine-based hydrazone derivatives

**Scheme 4:**
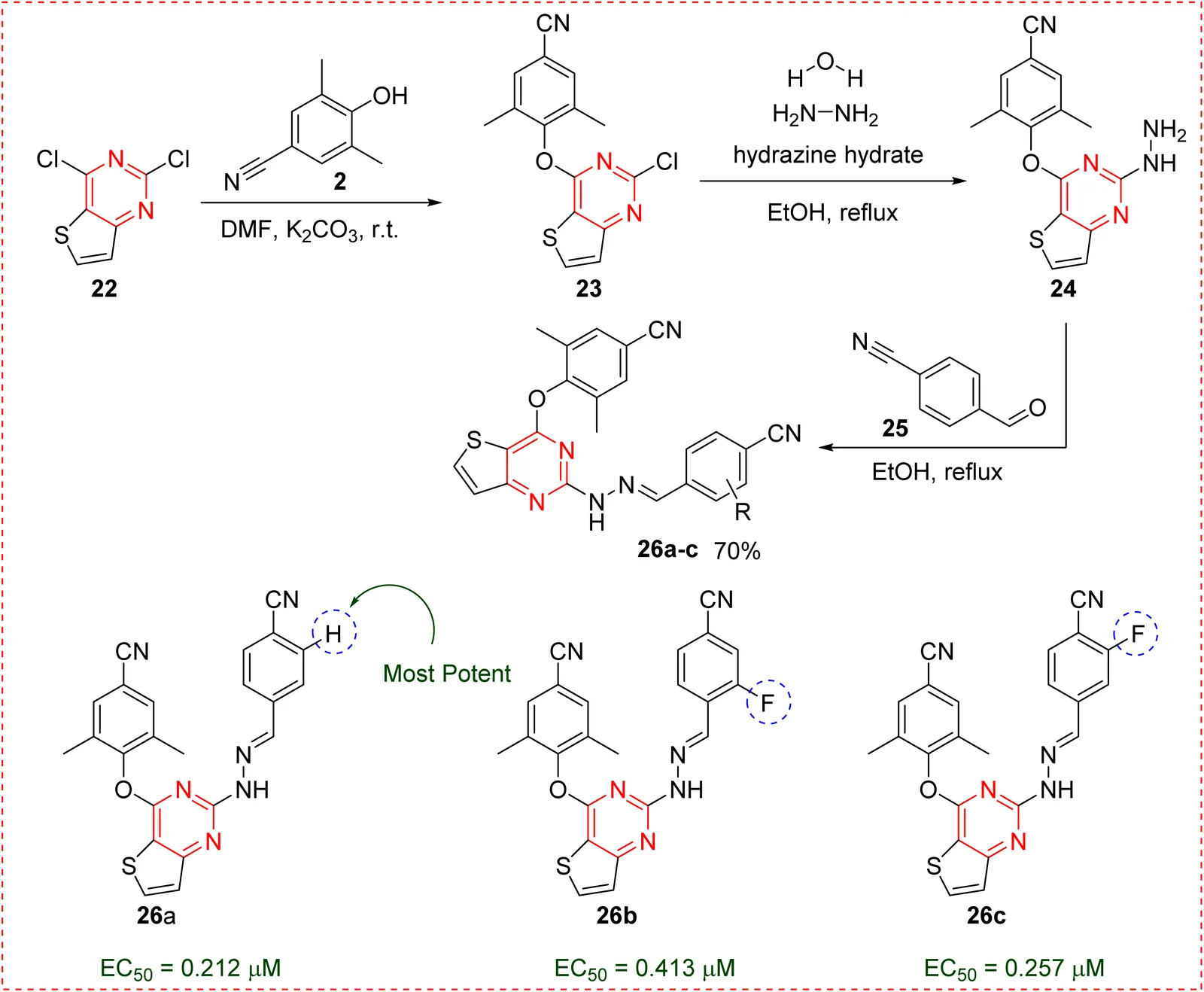
Synthesis of pyrimidine derivatives 26a–c.

A number of pyrimidine-based hydrazine derivatives (26a–c) with a modified linker between the right wing and the thienopyrimidine core were designed, synthesized, and biologically assessed by Wang *et al.* (Scheme 4). Excellent HIV-1 inhibitory efficacy was shown by some of them. The starting material, thieno-based pyrimidine (22), underwent nucleophilic substitution with 3,5-dimethyl-4-hydroxybenzonitrile (2) to yield intermediate 23. After reacting with hydrazine hydrate under ethanol reflux, 23 produced intermediate 24, which condensed with different substituted 4-cyanobenzaldehydes (25) to produce the target chemicals (26a–c). Recrystallization and HPLC were used to minimize impurities, but there was very little detailed impurity profiling reported. Common reagents were used, and this made the reactions reproducible and cost-effective; however, the use of organic solvents posed environmental safety concerns. SAR analysis revealed that 26a was more potent as it was able to adopt a horseshoe conformation in the NNRTI binding pocket and was able to make strong π–π stacking interactions with Tyr181, Tyr188, Phe227 and Trp229, along with two hydrogen bonds with Lys101. This binding mode resulted in an EC_50_ of 21.2 nM and a very high selectivity index (SI = 8632), which was ideal for anti-HIV-1 action against K103N and Y181C.^96^

#### 2.4.2. Thieno[3,2-*d*]pyrimidines featuring a primary aniline

**Scheme 5:**
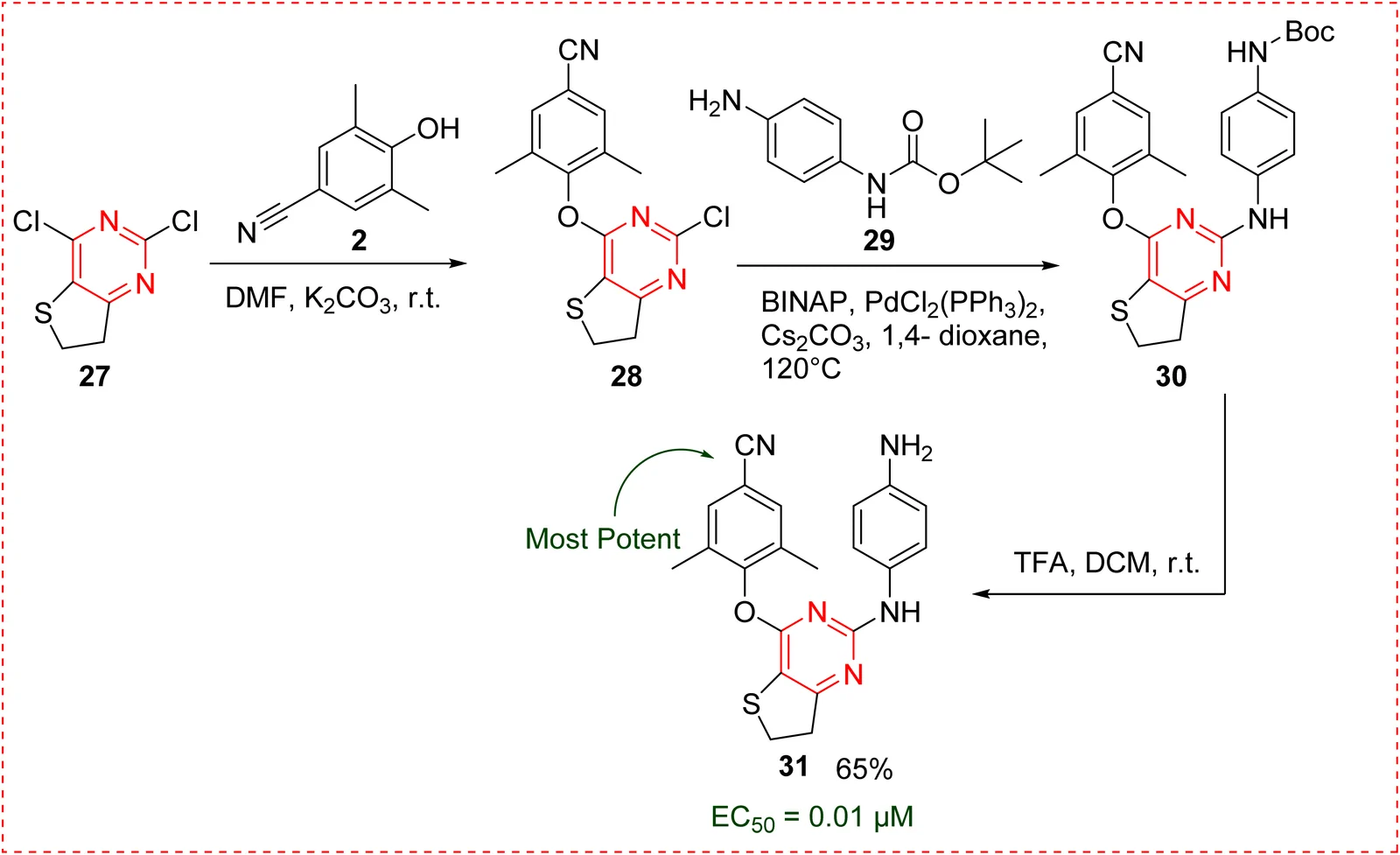
Synthesis of pyrimidine derivative 31.

A novel thieno-based pyrimidine (31) was created by Kang *et al.*, and it showed strong effectiveness against HIV-1 and resistant mutant strains (Scheme 5). The synthesis began when the starting material, dichlorothieno-based pyrimidine (27), was nucleophilically substituted with 4-hydroxy-3,5-dimethylbenzonitrile (2), yielding intermediate 28. This was followed by Buchwald–Hartwig amination with *t*-butyl(4-aminophenyl)carbamate (29) to afford intermediate 30, which was catalyzed by palladium and underwent acidic Boc deprotection to yield the target compound (31). The presence of palladium in the catalytic steps and the use of toxic solvents limited the route to the laboratory scale only. Residual catalysts, sulfonyl chlorides and unreacted intermediates were the major sources of impurities. The reactions were reproducible but not cost-effective because of the use of expensive reagents and moderate yields, and environmental safety was limited because they generated waste and used hazardous solvents. SAR analysis showed that the overall drug resistance profile was greatly enhanced by preserving the aniline moiety. As a result, compound 31 proved to be the most effective inhibitor, demonstrating remarkable antiviral activity against both the common K103N and E138K mutant strains of HIV-1.^97^

#### 2.4.3. Thieno[3,2-*d*]pyrimidines bearing a biphenyl ether linkage

**Scheme 6:**
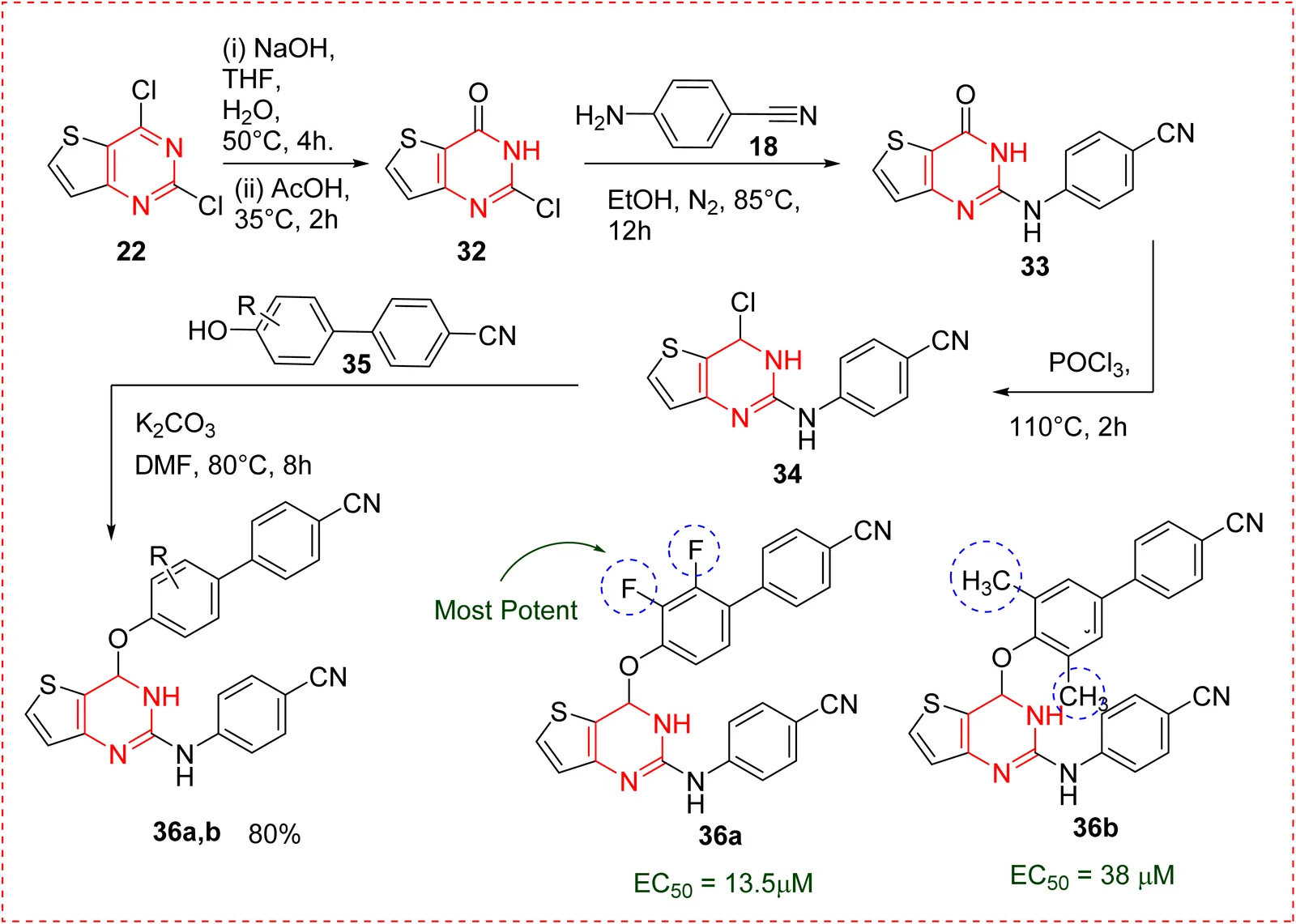
Synthesis of pyrimidine derivatives 36a, b.

By the structural optimization of biphenyl DAPYs, Sang *et al.* identified a series of new thieno-based pyrimidine analogues (36a, b) by a scaffold-hopping strategy (Scheme 6). The synthesis of 36a, b was initiated when selective hydrolysis occurred at 22 to yield 32. The subsequent amination of 32 resulted in the formation of intermediate 33, which was further chlorinated to 34. In the last step, 34 underwent nucleophilic substitution with substituted 35 in the presence of a base to yield the final products (36a, b). No unusual impurities were found in the profile; HPLC purities were typically greater than 75%. Reactions were robust under the optimized conditions, moderately environmentally problematic (given the use of chlorinated solvents and POCl_3_), and cost-effective compared to earlier analogues. The structure–activity relationship (SAR) analysis showed that the introduction of flexible linkers and electron-donating groups at the central pyrimidine enhanced potency, while bulky hydrophobic groups attached to the terminal ring improved binding affinity to the NNRTI pocket. Activity was enhanced by substitutions at positions that could allow π–π stacking with Tyr181 and Tyr188 and modifications that reduced steric clash with Lys103. Activity was increased by substitutions at positions that could allow π–π stacking with Tyr181 and Tyr188 and truncations that reduced steric clash with Lys103. Moreover, the synthesized 36a was the most potent inhibitor and exhibited high efficiency against K103N, L100I, Y181C and E138K.^98^

#### 2.4.4. Piperidine-based thiophene[3,2-*d*]pyrimidine scaffolds

**Scheme 7:**
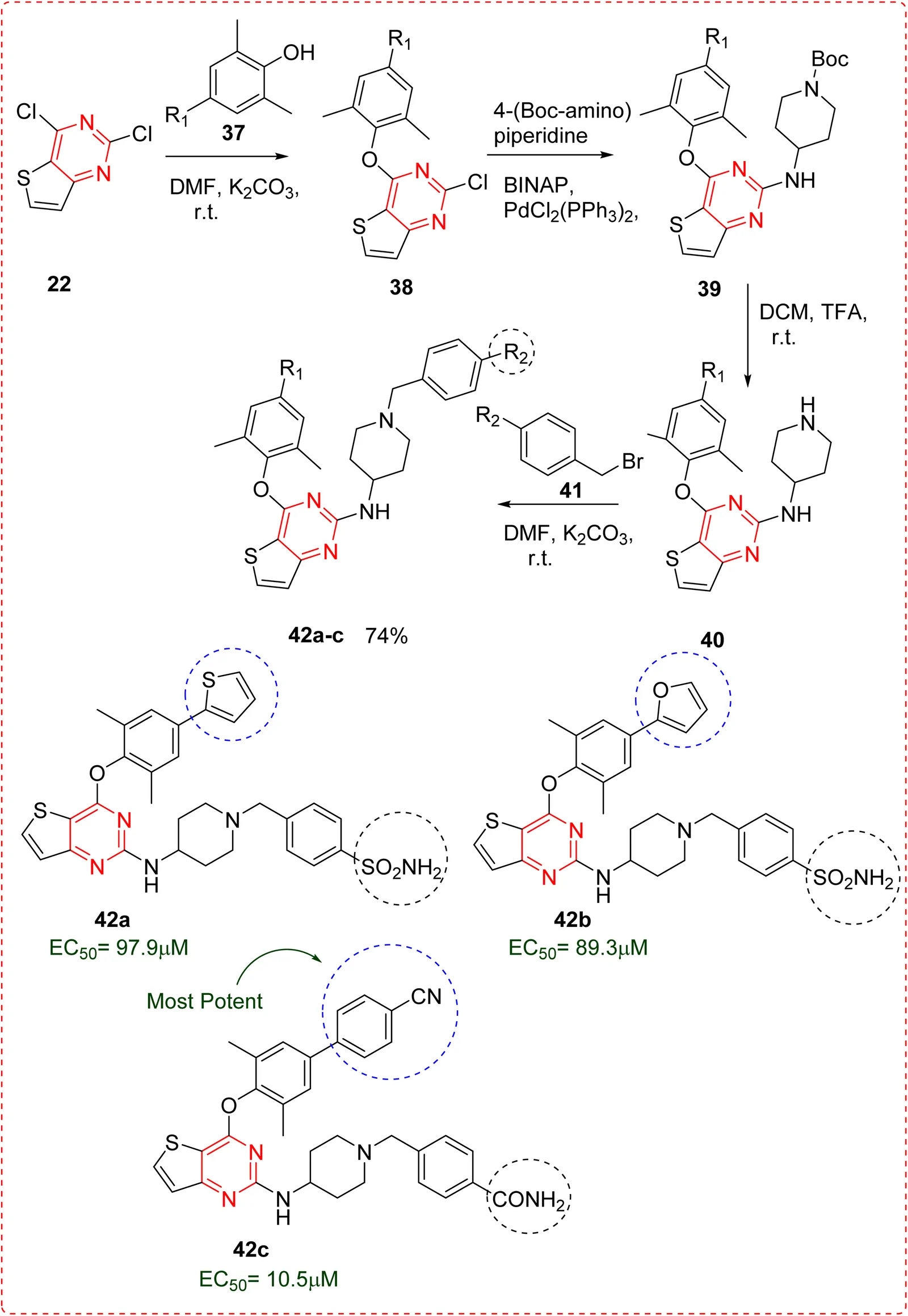
Synthesis of pyrimidine derivatives 42a–c.

The synthesis of piperidine-substituted pyrimidine derivatives (42a–c), which turned out to be potent for single mutations in HIV-1 and wild-type strains, was reported by Kang *et al.* (Scheme 7). The synthesis initiated upon the nucleophilic substitution of commercially available piperidine-based pyrimidine (22) with substituted phenol (37) to afford intermediate 38. In the next step, intermediate 38 reacted with Boc-protected piperidine to generate intermediate 39. Upon removing the Boc protecting group of 39, the key intermediate 40 was obtained, which, upon reacting with substituted benzyl bromide, afforded target compounds (42a–c). Multi-step Pd-catalyzed couplings were problematic for the synthesis of the 42a–c NNRTIs because they generated impurities from side products, which needed to be carefully purified. These reactions were only carried out at the laboratory scale and were demonstrated as moderately robust reactions, but they have not yet been optimized for greener solvents or for industry cost-effectiveness. The processes were based on DMF and halogenated reagents, which could raise concerns regarding environmental safety. The SAR analysis revealed that phenyl ring substitution at position R_1_ enhanced the antiviral activity compared to thiophene or the furan ring. 42b emerged as the most potent against the HIV-1 L1001 mutation and also demonstrated activity against resistant strains like K103N and Y181C.^99^

#### 2.4.5. Thieno[3,2-*d*]pyrimidines featuring a terminal fluorosulfate

**Scheme 8:**
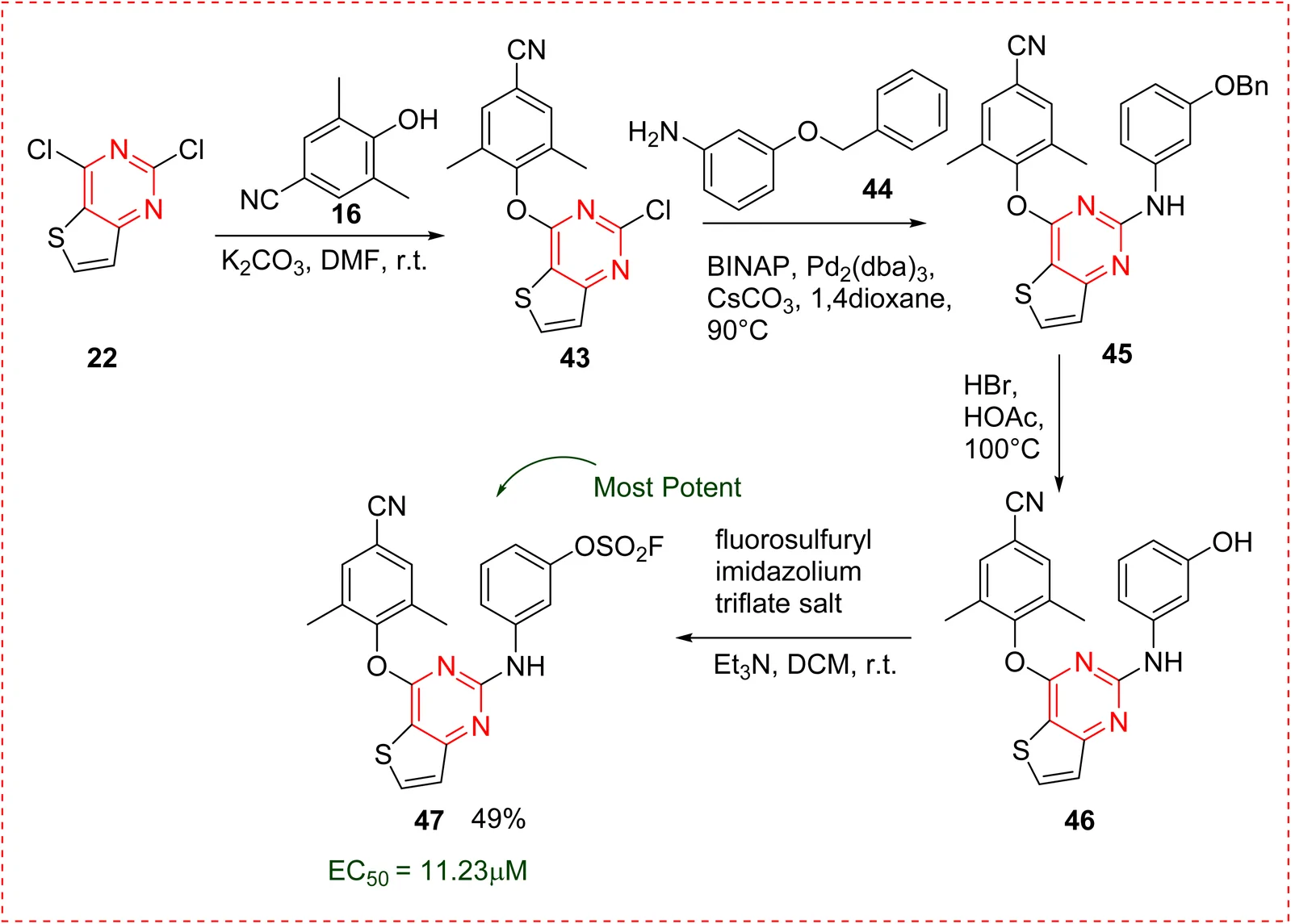
Synthesis of pyrimidine derivative 47.

A novel covalent thienopyrimidine derivative (47) was synthesized with the aim of strategically targeting the highly conserved Tyr318 residue present in the binding pocket of the HIV-1 reverse transcriptase (RT) by Zhou *et al.* (Scheme 8). The chemical synthesis started from the starting material 22 by nucleophilic substitution with 4-hydroxy-3,5-dimethylbenzonitrile (16) and eventually produced intermediate 43. Intermediate 43 was then reacted with 3-(benzyloxy)aniline (44) in the sequential step *via* the palladium-catalyzed Buchwald–Hartwig cross-coupling reaction to generate intermediate 45. The acidic deprotection of compound 45 in the presence of hydrogen bromide in acetic acid gave intermediate phenol 46. Finally, the precursor compound 46 was reacted with the fluorosulfuryl imidazolium salt, and it was found to be an efficient source of the target compound (47). Tissue-based bioactivity assessments showed that the addition of a fluorosulfate warhead allowed one-to-one covalent association with the target enzyme, leading to a dramatically enhanced drug resistance profile and, at the same time, low cytotoxicity.^100^ Thus, compound 47 proved to be the most effective covalent inhibitor, displaying potent and broad-spectrum antiviral activity against wild-type HIV-1 as well as against common NNRTI-resistant mutants such as K103N, E138K and L100I and the very resistant F227L and V106A double mutants.^101^

#### 2.4.6. Amino acid (analogue)-substituted thiophene[3,2-*d*]pyrimidines

**Scheme 9:**
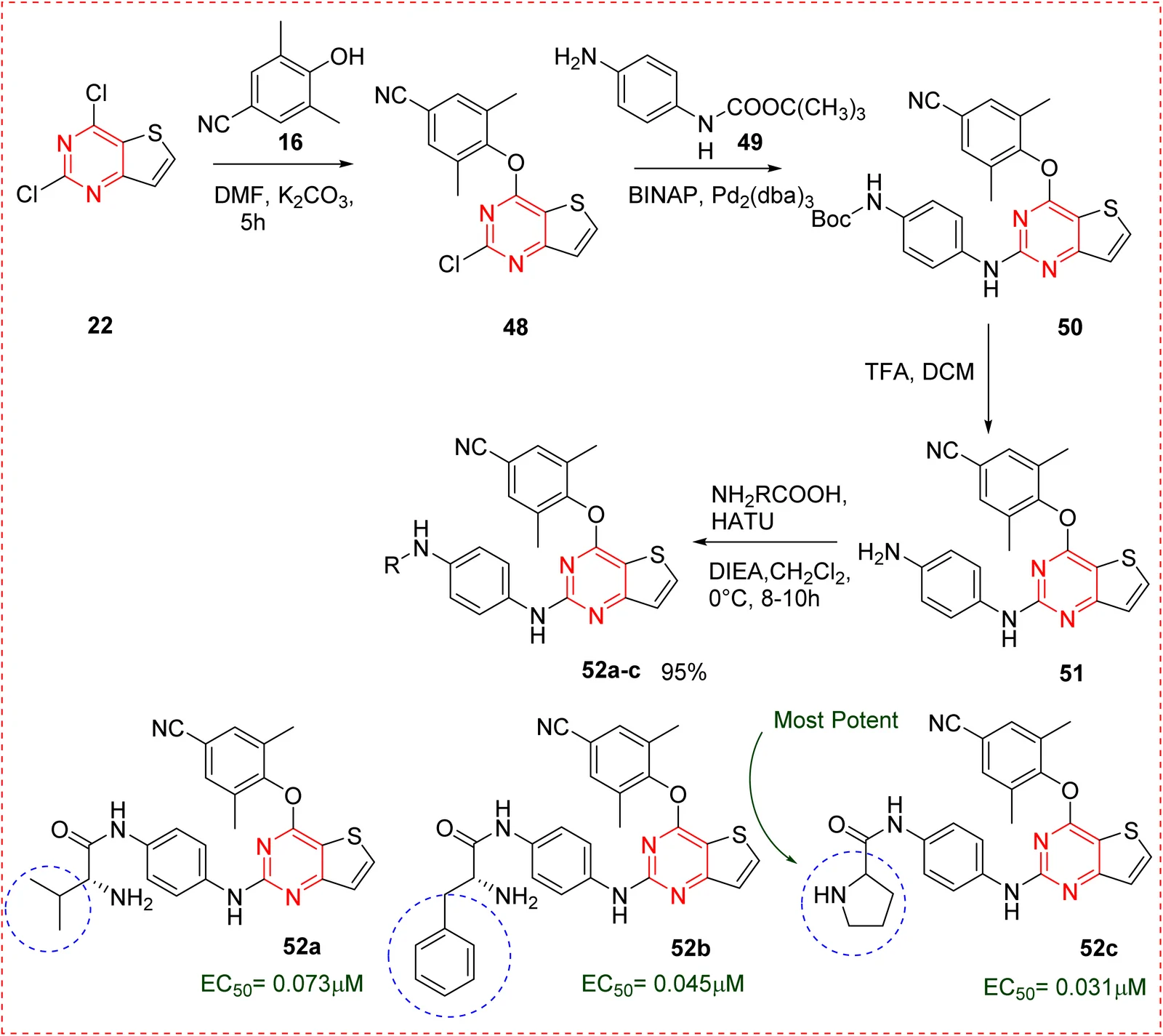
Synthesis of pyrimidine derivatives 52a–c.

Zhu *et al.* synthesized amino acid-substituted thiophene pyrimidines (52a–c) that showed activity against different single-mutant HIV-1 strains with higher selectivity (Scheme 9). The synthesis initiated when commercially available 22 underwent nucleophilic substitution with 4-hydroxy-3,5-dimethylbenzonitrile (16) to afford intermediate 48. In the next step, intermediate 48 generated intermediate 50 upon reacting with *tert*-butyl (4-aminophenyl)carbamate (49), which, upon reacting with trifluoroacetic acid (TFA), yielded compound 51. In the next step, compound 51, upon amide condensation with various amino acids at 0 °C using *N*,*N*-diisopropylethylamine (DIEA), generated the target compounds (52a–c). Impurity profiles were feasible, and at the Gram level, they could be purified to achieve the desired purity; however, large-scale purification beyond the gram scale has not yet been proven. The cost-effectiveness was moderate due to the use of palladium catalysts and the use of special reagents, while the environmental safety value was acceptable because the reaction was carried out under mild conditions with standard solvents. Amino acid substituents with non-polar groups were found to be more potent than those with polar groups, according to SAR analysis. The additional amino acid substitution on the right side of compound 52c resulted in stronger hydrogen bonding in the solvent-exposed area and preserved the classical horseshoe shape and hydrophobic interactions, leading to 52c being more potent. It showed good activity against the HIV-1 K103N, E138K, Y181C, Y188L, L100I and F227L/V106A strains but was only moderately active against K103N + Y181C (RES056). SAR analysis revealed that by substituting R with a non-polar amino acid groups, anti-viral activity was enhanced, while polar groups reduced the activity.^102^

### 2.5. Diarylpyrimidines (DAPYs)

**Scheme 10:**
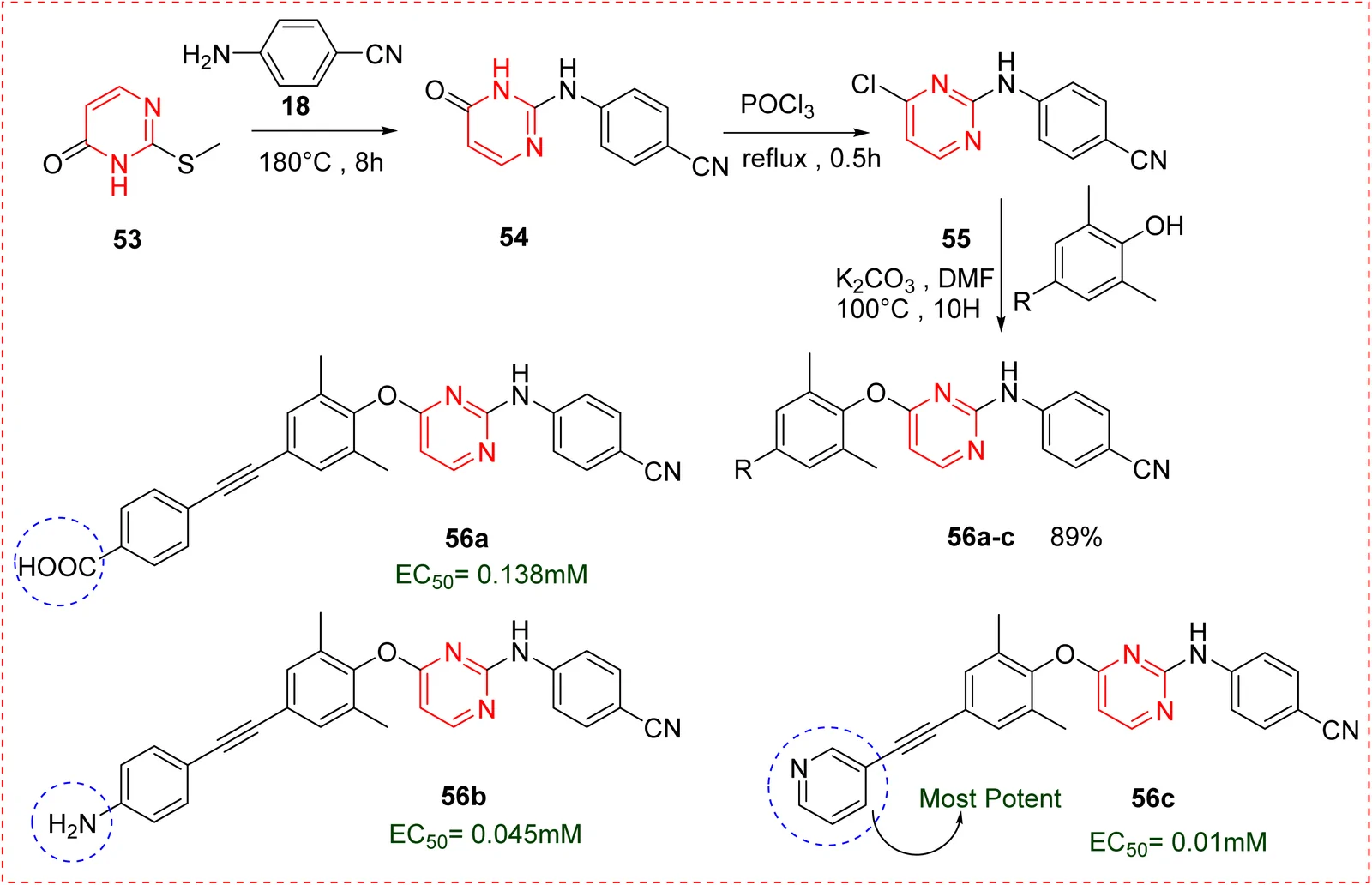
Synthesis of pyrimidine derivatives 56a–c.

Zhou *et al.* reported the synthesis of 56a–c, which belong to the class of HIV NNRTIs, known for their high potency and moderate cytotoxicity (Scheme 10).^103^ The synthesis initiated when commercially available 53 was reacted with 4-aminobenzonitrile (18) to yield intermediate 54, which afforded compound 55 upon chlorination with POCl_3_. In the next step, compound 55 generated 56a–c upon substitution with different phenyl derivatives. The synthetic strategies to the 56a–c derivatives were comparatively simple but suffered from medium yields and required palladium-catalyzed coupling reactions that increased economic and environmental issues. The main impurities were formed by incomplete couplings and side reactions with alkynes/alkenes, but these impurities could be easily removed. Compound 56c had good activity against Y181C, Y188L, and F227L + V106A. To sum up, these compounds had good coverage of mutants, were less toxic than current drugs, and possessed interesting SAR properties, but the synthetic routes need to be improved for cost-effectiveness and the environmental impact. SAR analysis revealed that polar group substitution, such as amino groups, at the *para* position in the left phenyl wing enhanced activity; also, substituting the phenyl ring with pyridine further enhanced the antiviral activity, while the carboxylic group decreased the activity. Upon comparing *para* and *ortho* substitutions, it was found that *para* substitutions enhanced the activity.^104^

#### 2.5.1. Diarylpyrimidine derivatives

**Scheme 11:**
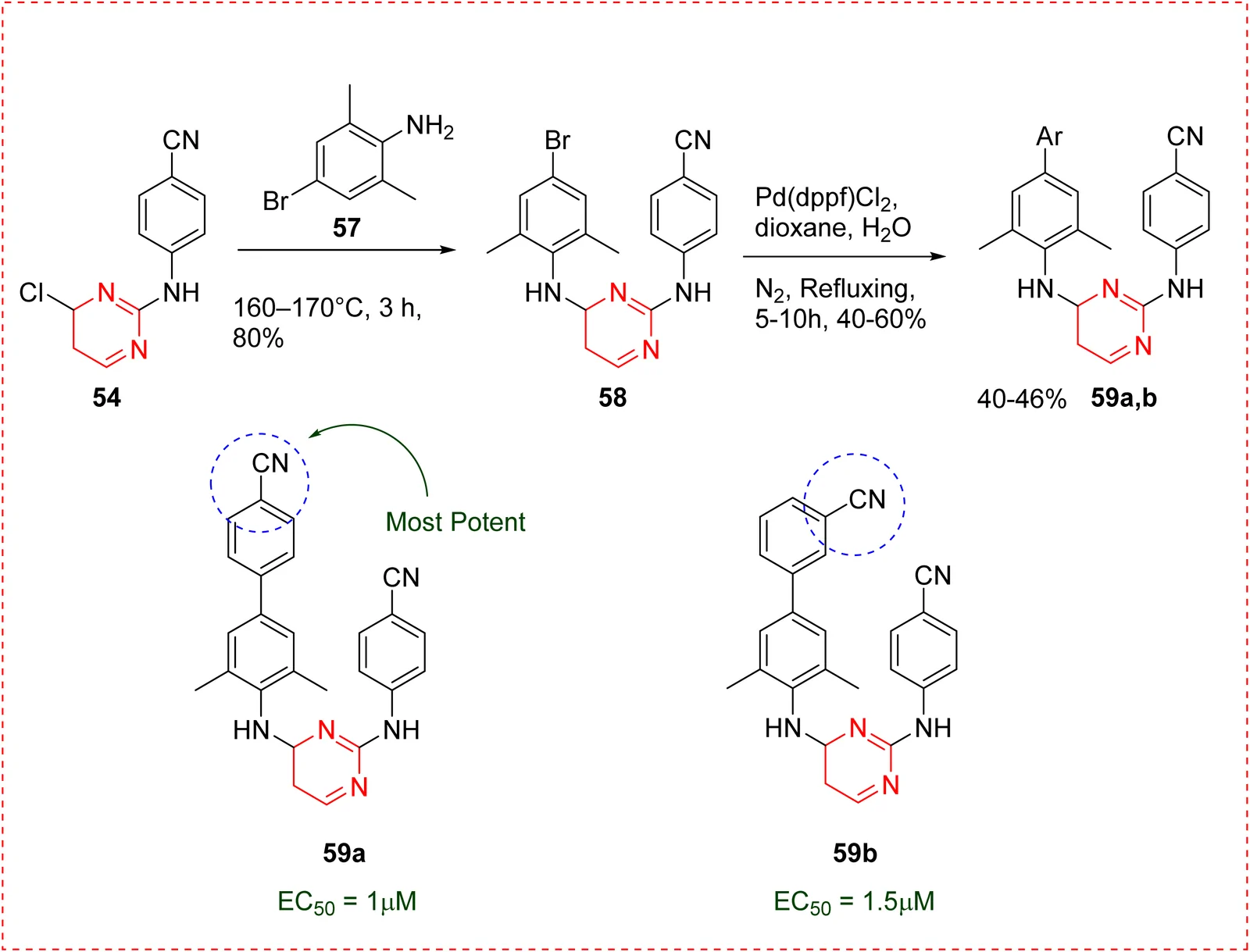
Synthesis of pyrimidine derivatives 59a, b.

Jin *et al*. developed a series of pyrimidine derivatives (59a, b) that target the hydrophobic portion of the non-nucleoside reverse transcriptase inhibitor binding pocket and possess anti-HIV-1 resistance properties (Scheme 11). Synthesis started with the previously synthesized 54, which was reacted with 57 at 160–170 °C to yield intermediate 58 through the Suzuki–Miyaura cross-coupling reaction with various arylboronic acids under refluxing nitrogen conditions using Pd(dppf)Cl_2_, yielding the target compounds (59a, b). Initial SAR analysis showed that incorporating the biphenyl ring to increase the π–π conjugated system led to a greatly improved aromatic interaction and antiviral activity against resistant variants. The most active overall was 59a, which showed high levels of activity against a broad range of wild-type HIV-1 variants and excellent activity against a wide panel of difficult single- and double-mutant variants, namely, L1001, K103N, Y181C, Y188L, E138K, and K103N/Y181C.^105^

#### 2.5.2. Diarylpyrimidine derivatives

**Scheme 12:**
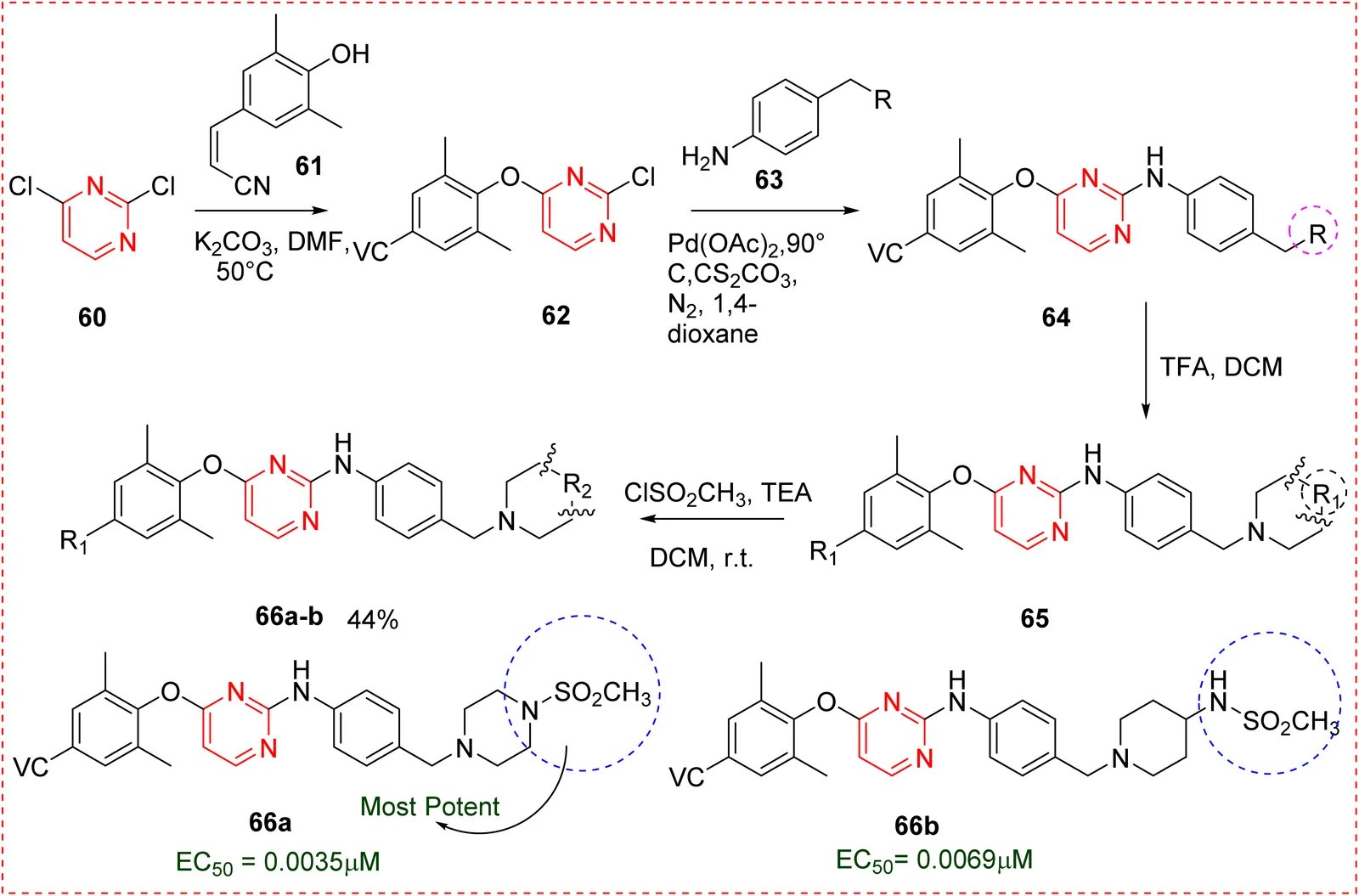
Synthesis of pyrimidine derivatives 66a, b.

Huang *et al.* developed novel pyrimidine derivatives (66a, b) that target the solvent-exposed tolerant region I of the HIV-1 reverse transcriptase and are highly water-soluble to overcome resistance conferred by the E138K variant and poor water solubility (Scheme 12). The synthesis began with the nucleophilic substitution of 2,4-dichloropyrimidine (60) with (*E*)-3-(4-hydroxy-3,5-dimethylphenyl)acrylonitrile (61) to give intermediate 62. The Buchwald–Hartwig coupling of intermediate 62 with a substituted 4-aminobenzyl derivative (63) gave intermediate 64. In the next step, the deprotection with trifluoroacetic acid (TFA) provided intermediate 65, which was converted to the desired compounds (66a, b) by reacting with methanesulfonyl chloride. SAR analysis showed that the inclusion of a cyanovinyl group at the left wing, in combination with hydrophilic methylsulfonyl-substituted piperazines at the right wing, had a substantial effect on antiviral activity and water solubility, and the rationale for exploring this combination was validated. The rationale for exploring this combination was validated by SARs showing that the cyanovinyl group at the left wing and hydrophilic methyl sulfonyl-substituted piperazines at the right wing had a substantial effect on antiviral activity and water solubility. 66a was the most active inhibitor of wild-type HIV-1 and was also highly potent against the more prevalent E138K and K103N mutant strains.^106^

#### 2.5.3. 1,2,3-Triazole-substituted diarylpyrimidine derivatives

**Scheme 13:**
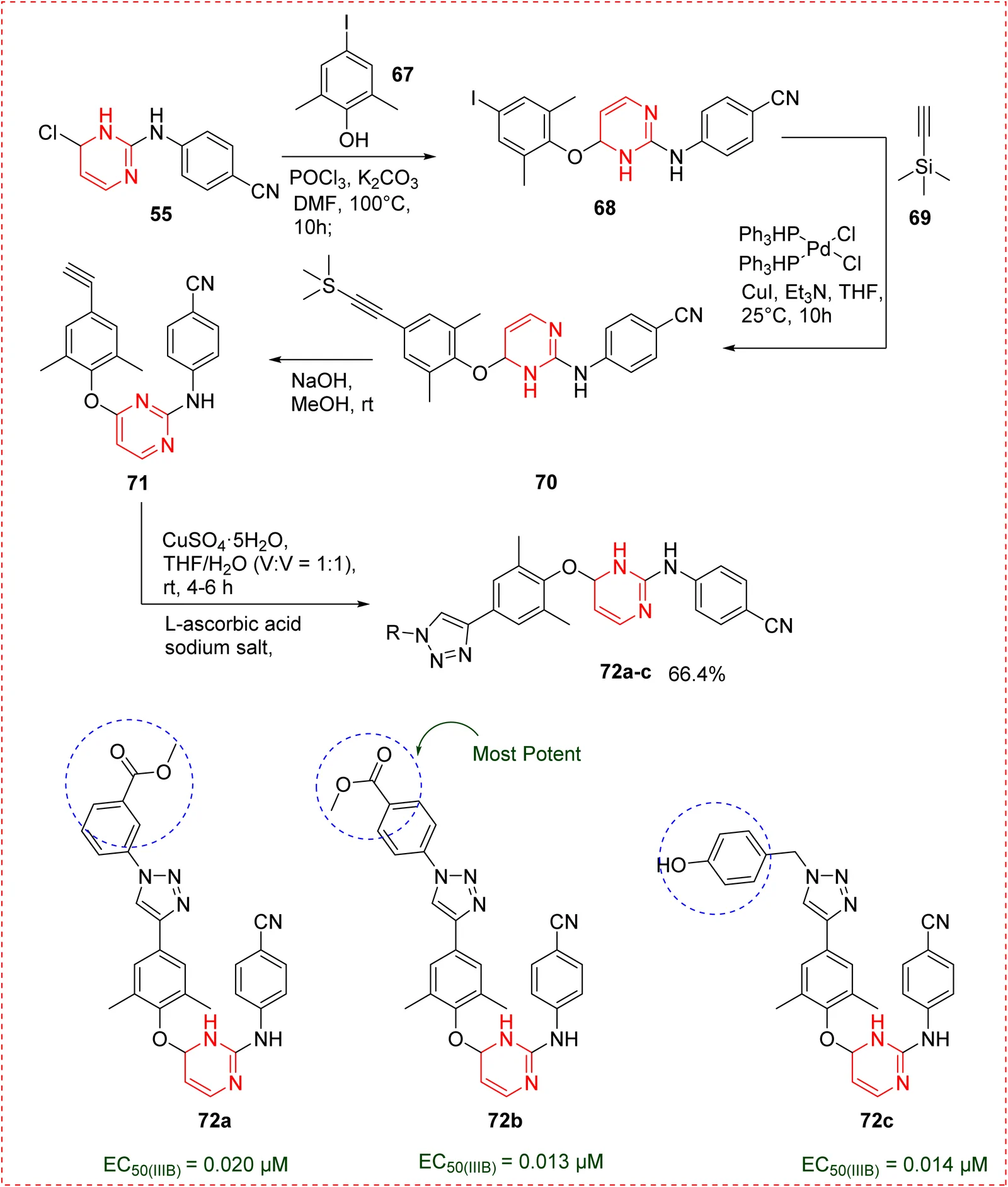
Synthesis of pyrimidine derivatives 72a–c.

To address the issue of drug resistance in HIV therapy, Zhou *et al.* developed and synthesized a series of novel compounds (72a, b) to bind to the HIV non-nucleoside inhibitor binding pocket (NNIBP), a hydrophobic channel. The previously synthesized compound 55 was reacted with 4-iodo-2,6-dimethylphenol (67) using potassium carbonate (K_2_CO_3_) as a base in DMF to obtain compound 68. The next step was to couple 68 with trimethylsilylacetylene (69) in the presence of a palladium catalyst and cuprous iodide to give intermediate 70, which was subsequently deprotected by reacting with sodium hydroxide in methanol to give terminal alkyne 71. Finally, upon the CuAAC “click” cycloaddition reaction of various substituted azides (with copper(ii) sulfate and sodium ascorbate) at room temperature, the target compounds (72a–c) were obtained (Scheme 13). SAR analysis showed that introducing substitutes on the triazole ring greatly affected the antiviral activity. Direct attachment of a rigid aromatic ring, *e.g.*, a methyl benzoate, to the triazole gave very good inhibitory activity, with *para*-substitution slightly superior to *meta*-substitution. Also, the use of flexible benzyl linkers necessitated the presence of hydrogen-bonding donors such as *para*-hydroxyl. These derivatives were highly effective against the dominant K103N mutant and had excellent resistance profiles. 72b emerged as the most potent compound against both wild-type HIV-1 and the K103N mutation.^107^

#### 2.5.4. Morpholine-substituted diarylpyrimidines

**Scheme 14:**
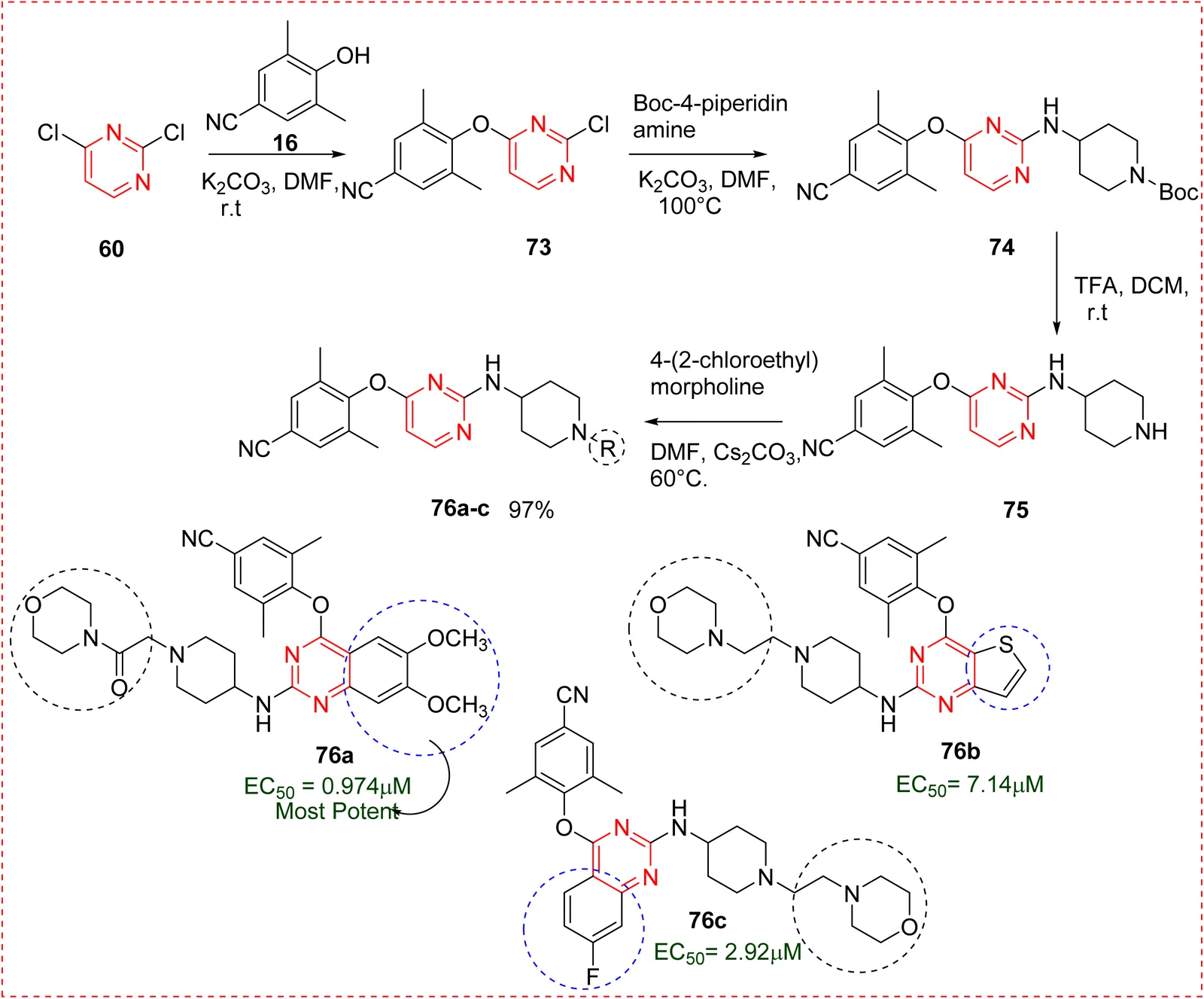
Synthesis of pyrimidine derivatives 76a–c.

Wang *et al.* reported the synthesis of 76a–c (Scheme 14) that showed activity against the wild-type HIV-1 strain and resistant strains. The synthesis initiated when commercially available 60, upon nucleophilic substitution with 4-aminobenzonitrile (16) using K_2_CO_3_, generated compound 73. In the next step, compound 73, upon reacting with Boc-4-piperidinamide, generated compound 74, which underwent deprotection in the presence of TFA to afford compound 75. In the final step, compound 75 generated the target compounds (76a–c) upon reacting with 4-(2-chloroethyl)-morpholine in the presence of DMF at 60 °C. The synthetic pathways to morpholine-containing diarylpyrimidines had relatively simple procedures, but the drawback was low activity against double-mutant strains. The overall cytotoxicity was lower than that of reference drugs, indicating a more favourable drug pharmacology. Reactions were conducted at a laboratory scale (millimolar), and the robustness of the reactions was shown by the consistent yield and reproducible antiviral activity. The moderate cost-effectiveness was due to the availability of morpholine and fused pyrimidine scaffolds, and the acceptable environmental safety was attributed to the use of common reagents and mild conditions. SAR analysis revealed that substituting the central core of pyrimidine with different aromatic rings influenced the activity, and the quinazoline core strongly enhanced the antiviral activity. Replacing R with acetyl morpholine instead of morpholine further enhanced the antiviral activity. 76a turned out to be most potent against double-mutant strains F227L + V106A.^108^

#### 2.5.5. Biphenyl-substituted pyrimidine oxime derivatives

**Scheme 15:**
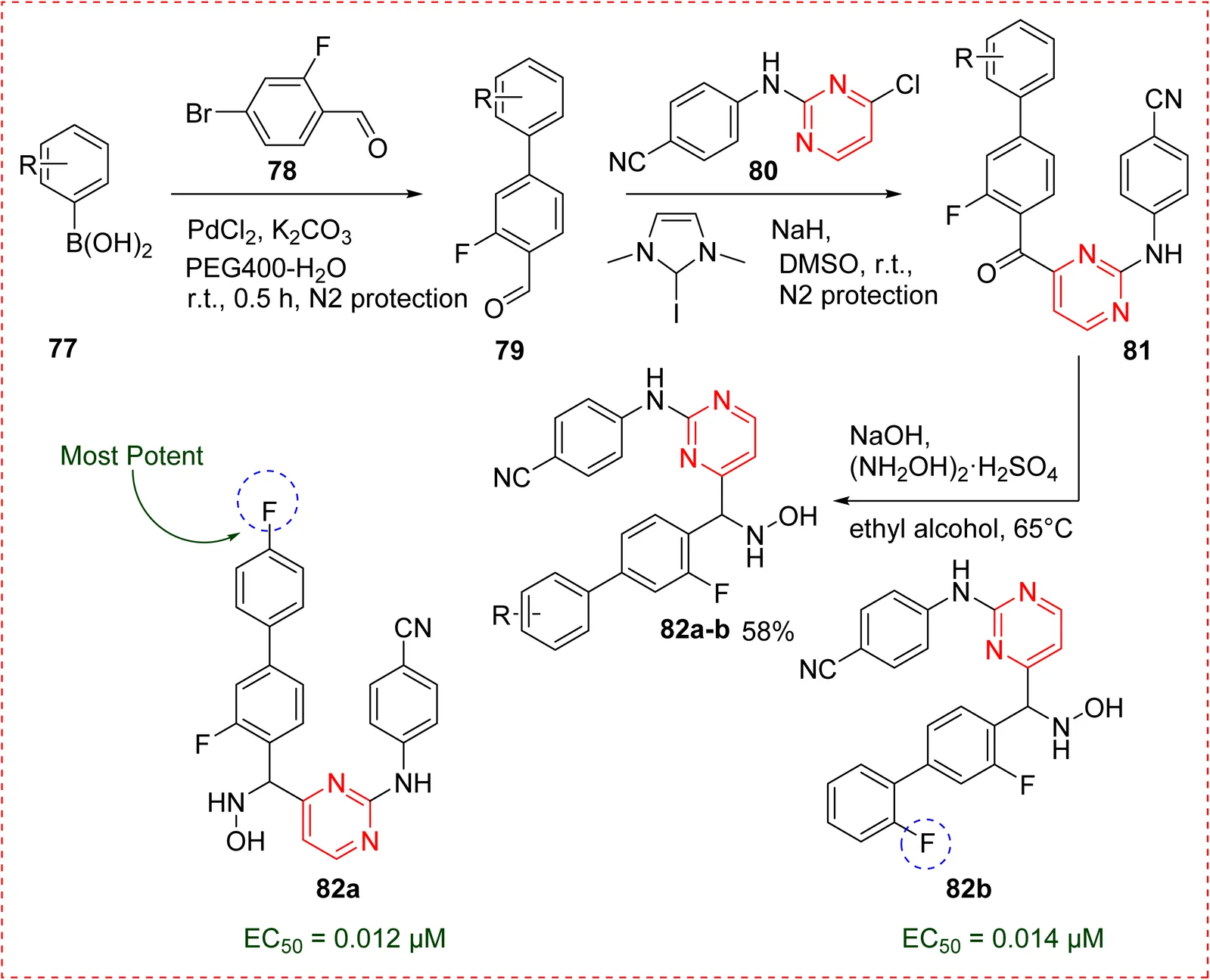
Synthesis of pyrimidine derivatives 82a, b.

Yang *et al.* designed and synthesized novel 82a, b with very promising antiviral activity against the wild-type and clinically observed HIV-1 mutants (Scheme 15). Commercially available 77 was used for the synthesis, which started with palladium-catalyzed cross-coupling with 4-bromo-2-fluorobenzaldehyde (78) in PEG400-H_2_O to give intermediate 79. Intermediate 79 reacted with 4-((4-chloropyrimidin-2-yl)amino)benzonitrile (80) with the help of 1,3-dimethylimidazolium iodide and NaH in DMSO to form intermediate 81. The target compounds (82a, b) were synthesized by the oximation of compound 82 with hydroxylamine sulfate in the presence of NaOH in ethyl alcohol at 65 °C in the next step. 82a, b were prepared by Pd-catalyzed coupling and oximation, but their hydrophobicity was a drawback, as it decreased their aqueous solubility and potentially impacted their bioavailability. The impurities were controlled by column chromatography and HPLC, and most of the compounds had >97% purity with moderate yields (35–65%). This method offered cost-effectiveness due to the use of commonly available reagents and solvents, but the handling of PEG400/H_2_O and NaH were important, and the method offered moderate environmental safety with typical concerns about the safety of PdCl_2_ and organic solvents. SAR analysis revealed that by substituting R on the biphenyl ring with *ortho*- and *para*-substituted moderate electron-withdrawing groups (such as fluorine), antiviral activity was enhanced, while *meta*-substitutions or electron-donating groups reduced the activity. 82a showed activity against wild-type and clinically relevant mutants of HIV-1, including E138K, L100I, K103N, and Y181C, but exhibited reduced activity against the double mutants K103N + Y181C and F227L + V106A.^109^

#### 2.5.6. Biphenyl-acetonitrile-substituted pyrimidine derivatives

**Scheme 16:**
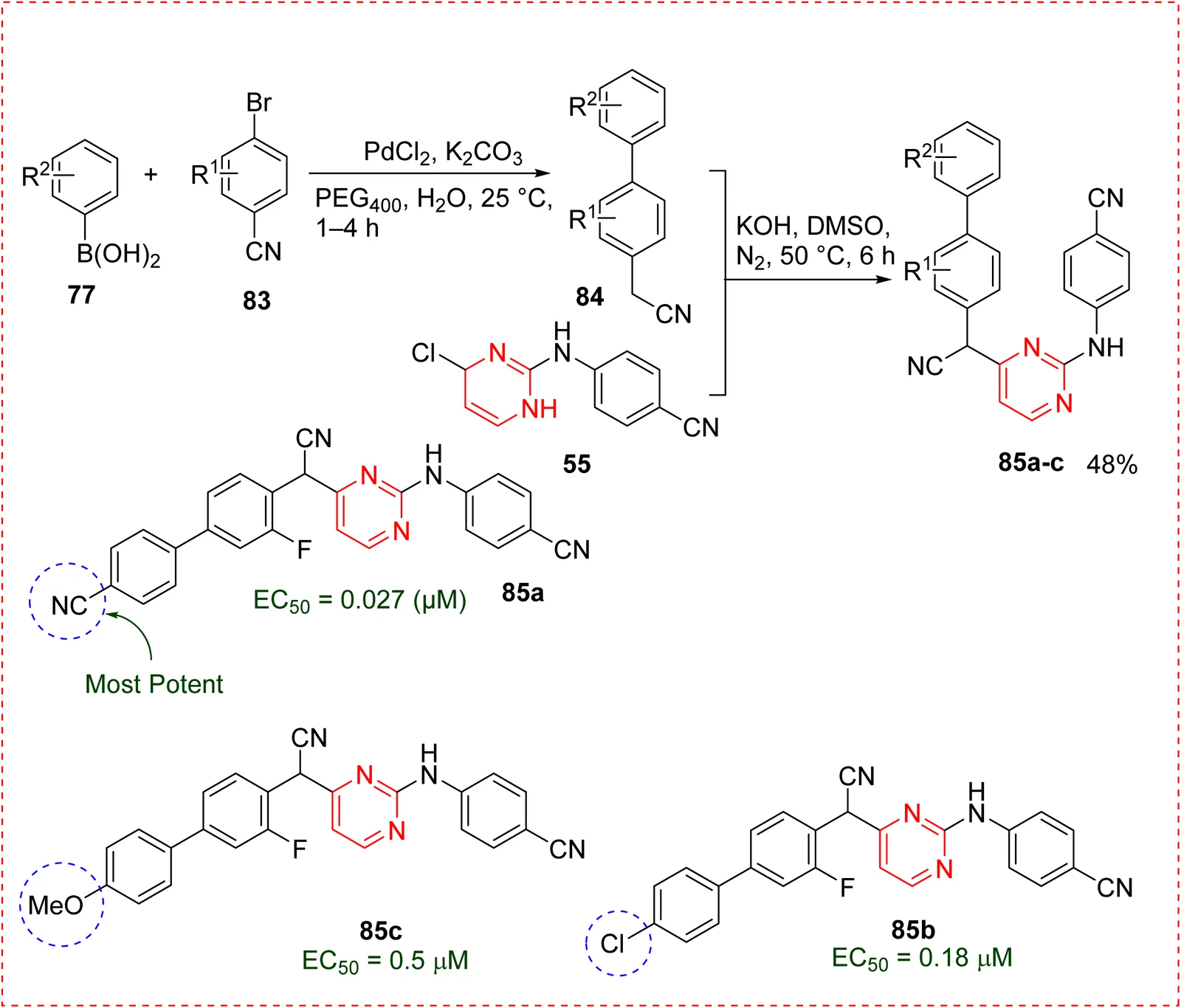
Synthesis of pyrimidine derivatives 85a–c.

Using a molecular hybridization approach, Lei *et al.* designed and synthesized a series of biphenyl-substituted diarylpyrimidines containing a cyanomethyl HIV-1 NNRTI connecting group, and these compounds were found to have moderate to good activities against wild-type HIV-1 and clinically relevant mutant strains of the virus. Key biphenyl acetonitrile intermediates (84) were synthesized by the Suzuki–Miyaura coupling reaction of commercially available arylboronic acids (77) with different substituted 2-(4-bromophenyl)acetonitriles (83) using PdCl_2_ and K_2_CO_3_ in PEG_400_/H_2_O at 25 °C for 1–4 h. The previously synthesized 135 was combined with 84 to give the required products 85a–c (Scheme 16). The antiviral activity of 85 was determined through SAR analysis, showing that groups with electron-withdrawing properties at the 4′-position of the biphenyl, such as CN, F, and Cl, significantly improved activity, whereas the electron-donating group OMe decreased activity. The 4′-position substitution was always found to be better than the 2′- and 3′-position substitutions. However, 85a proved to be the most active compound against wild-type HIV-1, with moderate activity against the single mutants K103N and L100I and no activity against HIV-1 double mutants K103N + Y181C and F227L + V106A.^110^

#### 2.5.7. Cyanophenyl acetonitrile-substituted pyrimidine derivatives

**Scheme 17:**
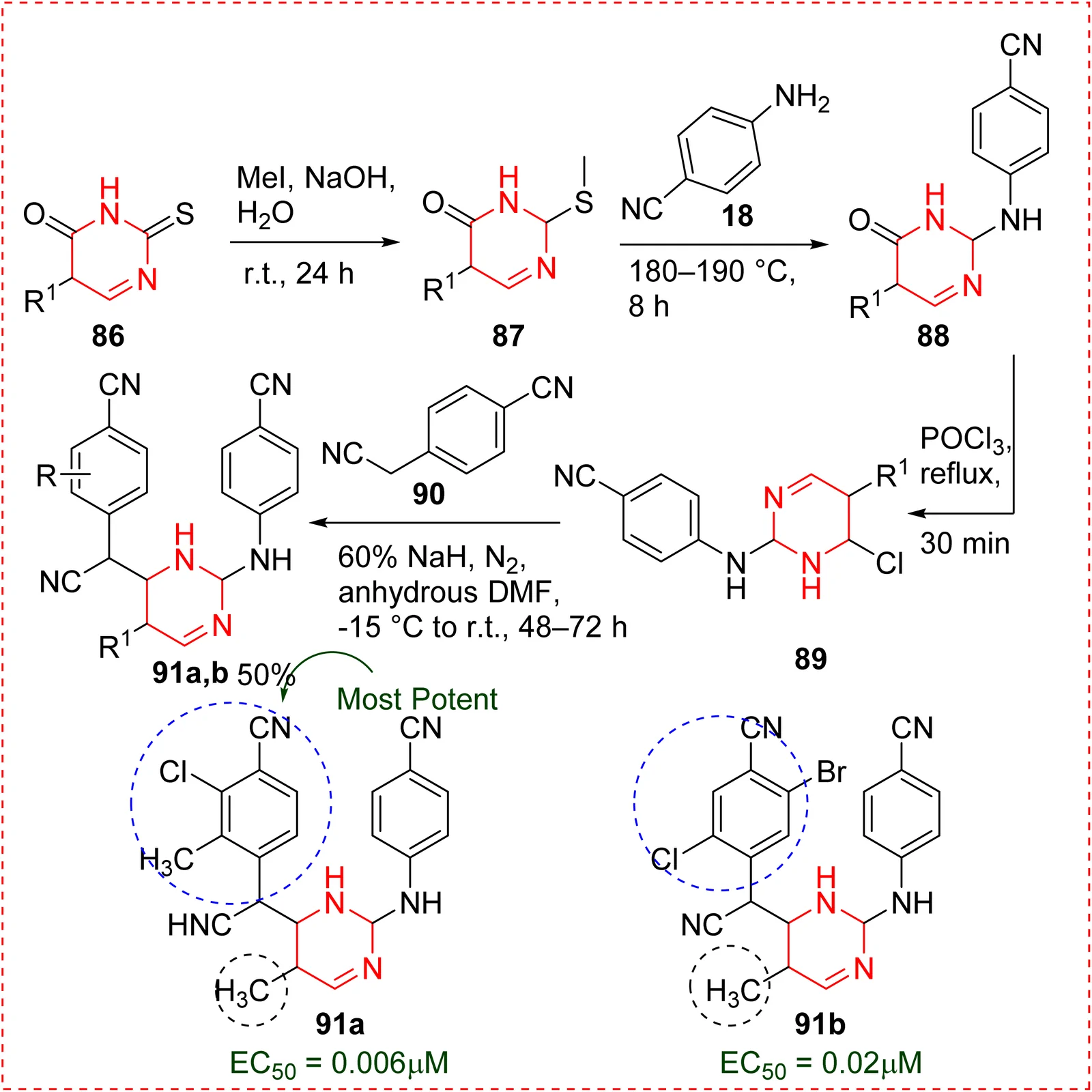
Synthesis of pyrimidine derivatives 91a, b.

Using a scaffold hopping approach, Li *et al.* designed and synthesized a new series of derivatives by replacing the cyano–methylene linker of CH(CN)-DABOs with the etravirine (ETR) scaffold, which demonstrated micromolar to nanomolar HIV-1 NNRTI antiviral activity against wild-type HIV-1. The treatment of commercially available thiouracil (86) with MeI and NaOH in H_2_O at room temperature for 24 h led to the formation of the methylthio intermediate 87 as the initial product of the synthesis. Intermediate 87 was condensed with 4-cyanoaniline (18) to yield intermediate 88; in the next step, at 180–190 °C for 8 h, the chloropyrimidine intermediate 89 was obtained by stirring intermediate 88 with POCl_3_ for 30 min under reflux. Finally, intermediate 89 gave the target compounds 91a, b upon treatment with substituted 4-cyanophenylacetonitriles under N_2_ in anhydrous DMF from −15 °C to room temperature for 48–72 h (Scheme 17). The 2-position of the 4-cyanophenyl group in 91a appeared to be the most preferred position for substitution to increase antiviral activity, with the most favorable substituents being electron-withdrawing halogens and the least being a methyl group; the introduction of a methyl group at the C5-position of the central pyrimidine ring was found to promote anti-HIV-1 activity by 6-30-fold compared to nonmethylated compounds. The most effective compound against wild-type HIV-1, and moderately active against the single mutants K103N, E138K, L100I, and Y181C.^111^

#### 2.5.8. Piperidine-biphenyl-diarylpyrimidines

**Scheme 18:**
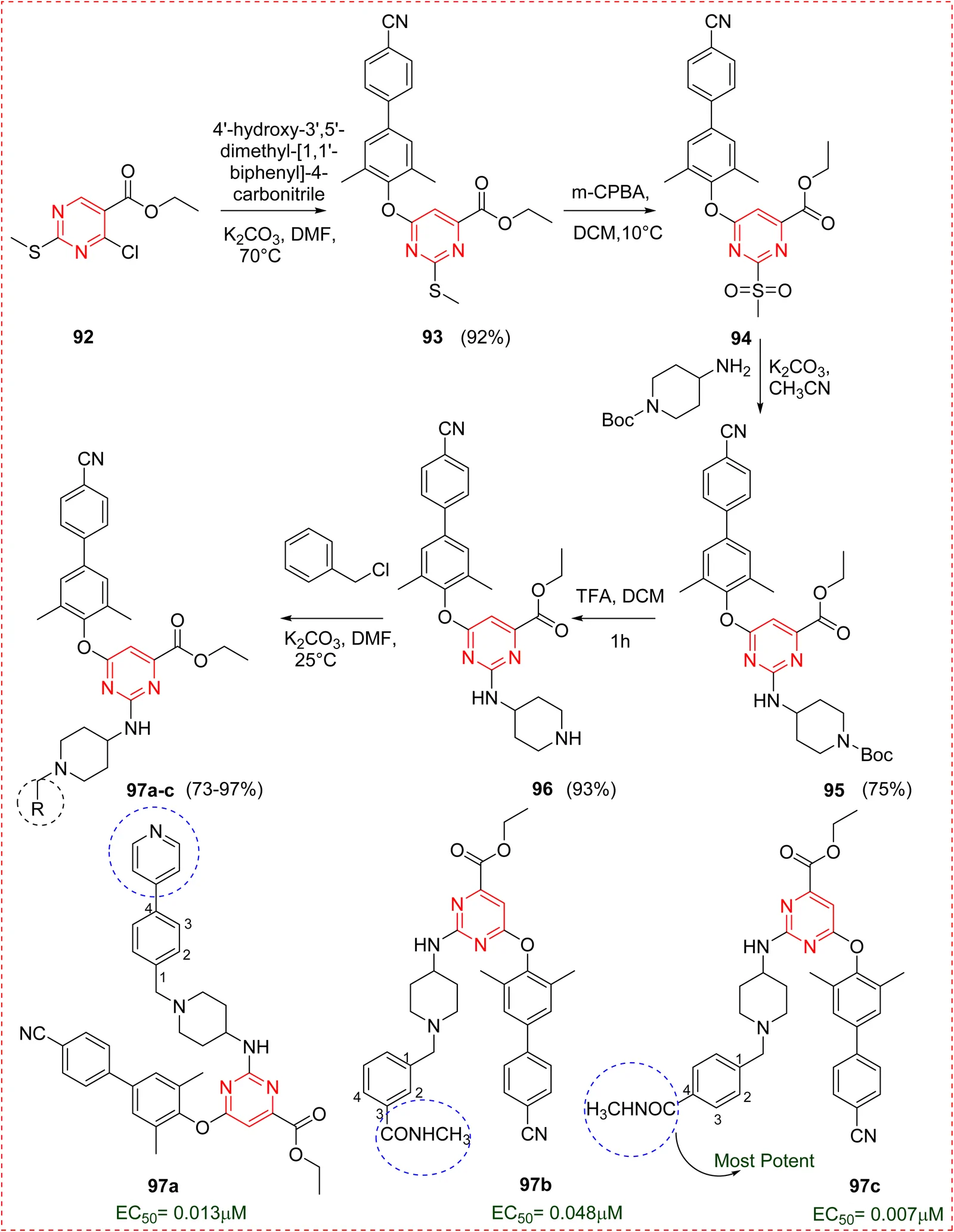
Synthesis of pyrimidine derivatives 97a–c.

Huang *et al.* reported the synthesis of 97a–c that showed strong activity against the WT HIV-1 strain (Scheme 18). The synthesis proceeded as commercially available 92 reacted with 4-cyano-4′-hydroxy-3′,5′-dimethyl-[1,1′-biphenyl]-4-carbonitrile in the presence of K_2_CO_3_ and generated compound 93, which, upon oxidation in the presence of *meta*-chloroperoxybenzoic acid (*m*-CPBA), afforded compound 94. In the next step, compound 94 generated key compound 95 upon nucleophilic substitution with Boc-protected amino piperidine, which, upon reacting with TFA, afforded compound 96. In the presence of K_2_CO_3_, the reaction between benzyl chloride and compound 96 generated the target compounds (97a–c). SAR analysis revealed that replacing the R group with the substituted phenyl ring enhanced the potency, and the position of the substituent on the phenyl ring also influenced the activity. Substituting *N*-methyl amide groups at the C_4_ position of the phenyl ring enhanced the antiviral activity, but substituting them at the C_3_ position reduced the activity. 97c emerged as the most active inhibitor of the HIV-1 single mutation K103N and also showed potency against Y181C.^112^

#### 2.5.9. 5-Substituted diarylpyrimidines

**Scheme 19:**
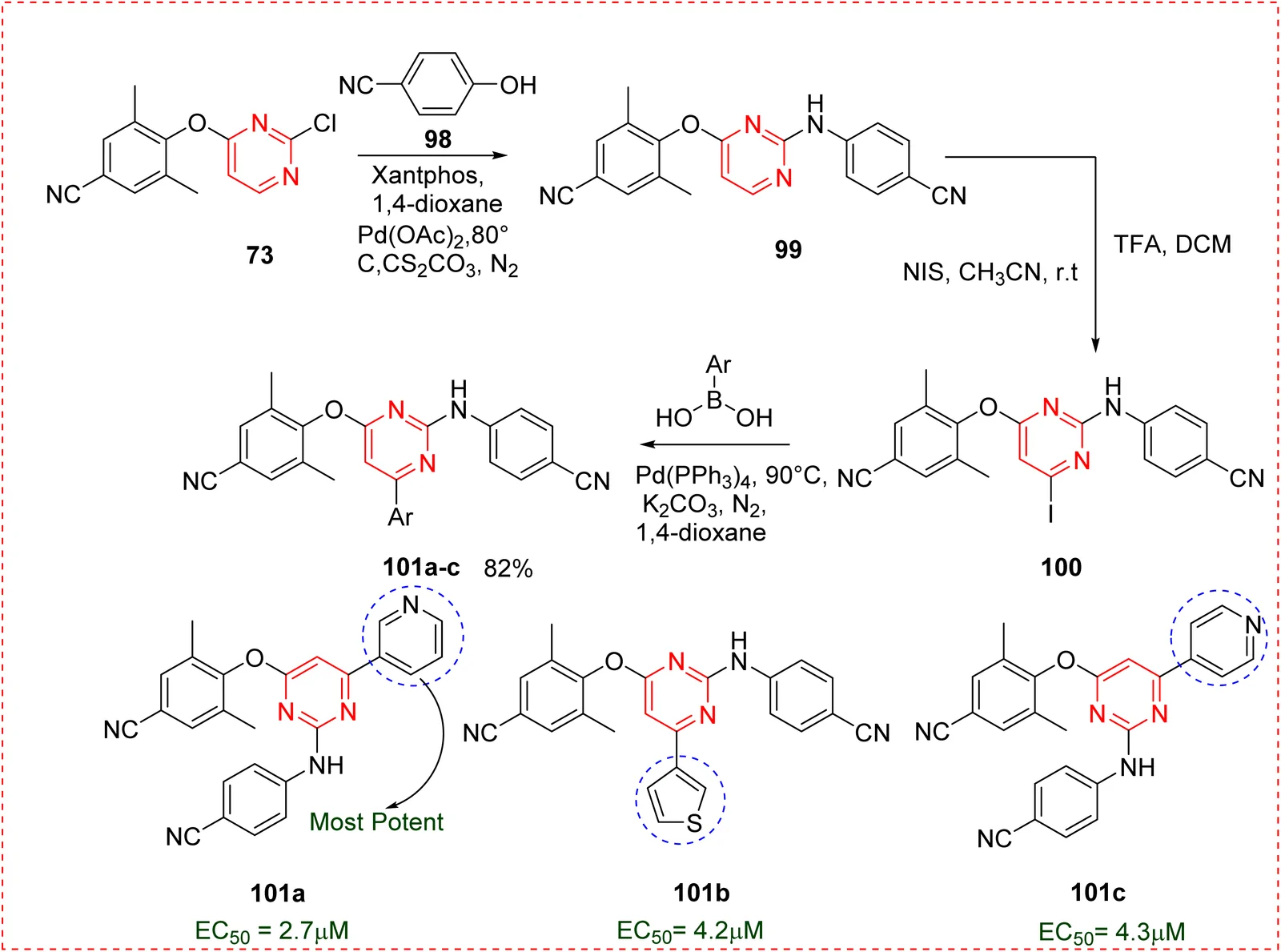
Synthesis of pyrimidine derivatives 101a–c.

Gao *et al.* synthesized 101a–c that demonstrated strong potency against the HIV-1 IIIB strain and showed moderate potency against single mutants (Scheme 19). The synthesis initiated when the previously synthesized 73, upon reacting with 4-aminobenzonitrile (98) in the presence of CS_2_CO_3_, afforded compound 99, which, in the presence of NIS and TFA, generated compound 100. In the final step, compound 100, with aryl-substituted boric acid in the presence of K_2_CO_3_, generated key compounds 101a–c. The reactions were reproducible and gave consistent products, but cost-effectiveness was restricted owing to the use of expensive catalysts and heteroaryl boronic acids, and environmental safety issues were raised by the use of organic solvents and heavy metal catalysts. The SAR analysis revealed that the potency was improved by the presence of heteroaryl substitutions (furyl, thienyl, and pyridyl), particularly in the case of resistant mutants. 101a emerged as the most active compound, with nanomolar activity against the wild-type HIV-1 and strong activity against mutants. These analogues were active against both wild-type and clinically relevant mutant strains, but further drug-likeness and safety optimization was required.^113^

### 2.6. Trisubstituted pyrimidines

#### 2.6.1. 2,4,6-Trisubstituted pyrimidine derivatives

**Scheme 20:**
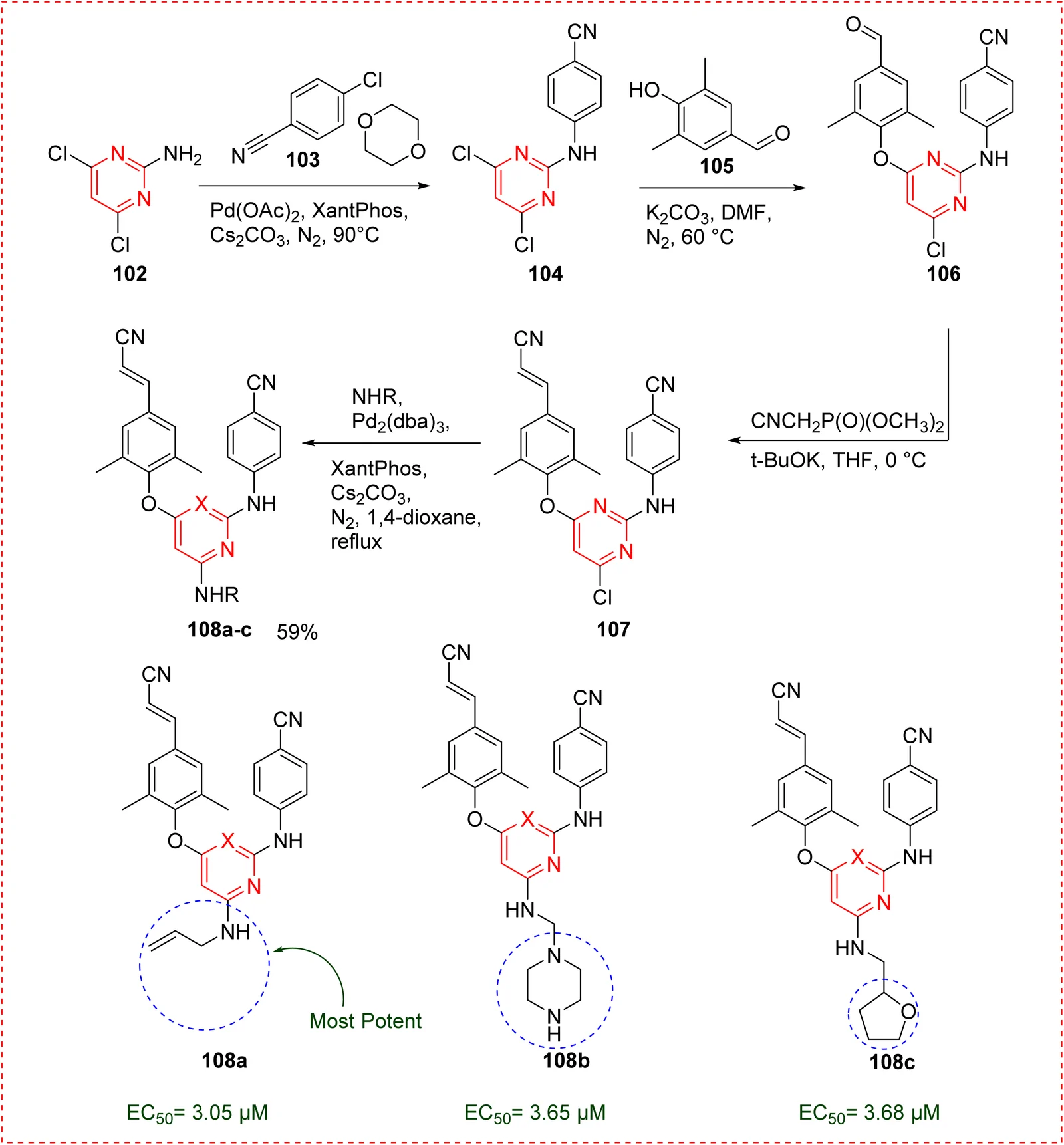
Synthesis of pyrimidine derivatives 108a–c.

Lu *et al.* reported the synthesis of a novel family of pyrimidine derivatives (108a–c) that inhibited the entrance channel of HIV-1 reverse transcriptase with high potency in both wild-type and mutant strains (Scheme 20). The synthetic pathway started with the commercially available 102, which was subjected to a cross-coupling reaction with 4-chlorobenzonitrile (103) in the presence of Pd(OAc)_2_, xanthos, and CS_2_CO_3_ under a nitrogen atmosphere at 90 °C to yield intermediate 104. Intermediate 104 was then reacted with 4-hydroxy-3,5-dimethylbenzaldehyde (105) in DMF with the aid of K_2_CO_3_ at 60 °C to form intermediate 106. Then, intermediate 106, upon reacting with dimethyl cyanomethylphosphonate at 0 °C in THF using *t*-BuOK, yielded compound 107. The final step involved a C–N cross-coupling reaction between compound 107 and various aliphatic amines with refluxing 1,4-dioxane in the presence of Pd_2_(dba)_3_ and xantPhos to afford the desired compounds (108a–c).

Heavy metal residues were a concern in this synthetic route, which involved palladium-catalyzed cross-coupling steps, for both cost and environmental reasons. Generally acceptable impurity profiles were obtained, although sometimes, pyridines containing impurities resulted in higher cytotoxicity and aggregation potential. It was found that the antiviral activity was improved by replacing the R position with a linear allylamino group, while bulky hydrophilic heterocycles showed a slight decrease in activity. Compound 108a was particularly active against wild-type HIV-1 and showed activity against common mutants like K103N, Y181C, and E138K and double mutants including K103N/Y181C.^114^

#### 2.6.2. 2,4,5-Trisubstituted pyrimidines

**Scheme 21:**
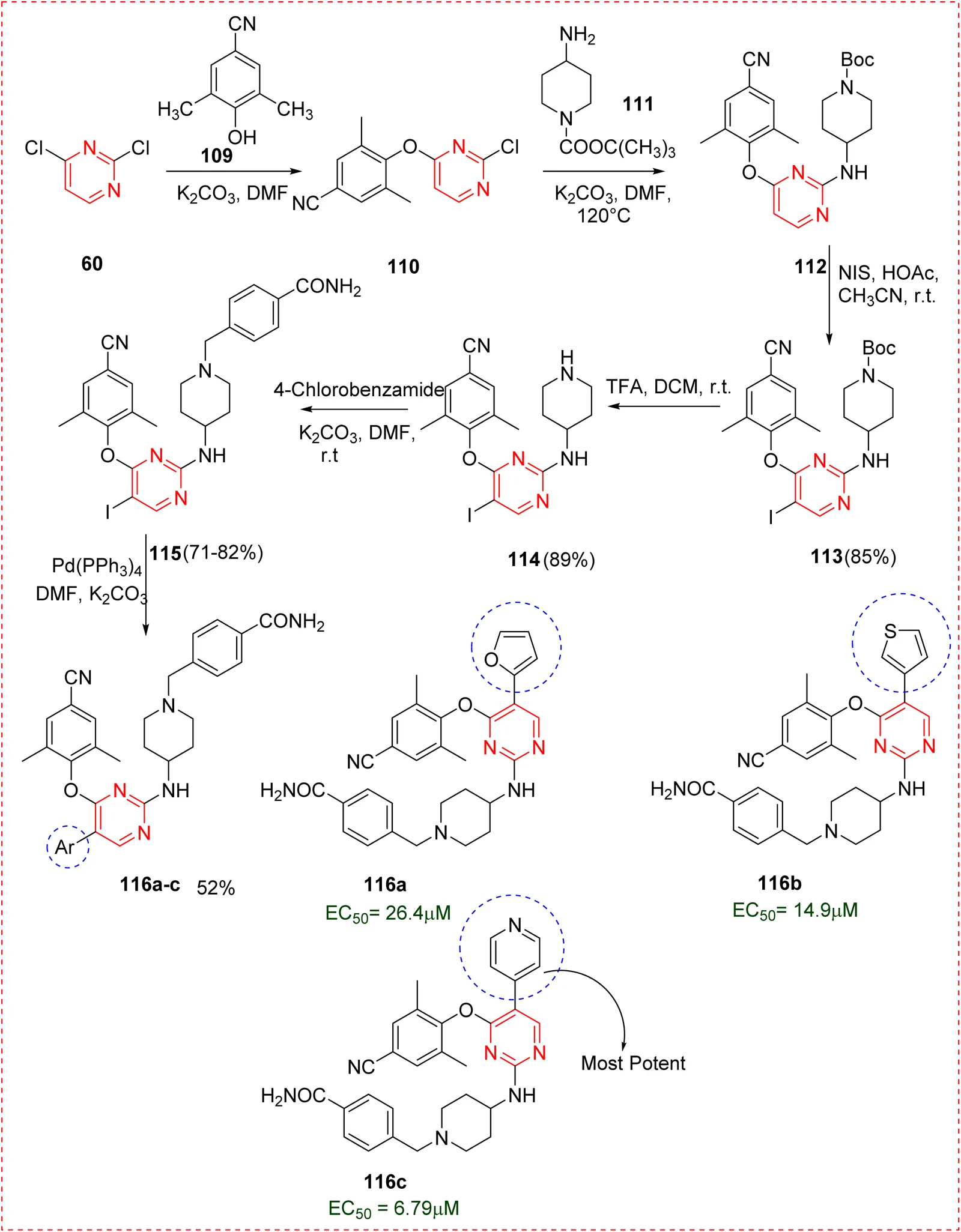
Synthesis of pyrimidine derivatives 116a–c.

Kang *et al.* synthesized the tri-substituted pyrimidine derivatives 116a–c. These pyrimidines showed potential against single-mutation HIV-1 strains. The synthesis initiated when commercially available dichloropyrimidine (60) reacted with 4-hydroxy-3,5-dimethylbenzonitrile (109), and intermediate 110 was obtained, which generated compound 112 upon a nucleophilic reaction with Boc-protected piperidine (111). In the next step, upon treatment with *N*-iodosuccinimide and acetic acid, compound 112 afforded compound 113, which reacted with TFA to generate product 114. Upon treatment with 4-chlorobenzamide, compound 114 afforded the key intermediate 115, which engaged in the Suzuki reaction in the presence of Pd(PPh_3_)_4_ and yielded the target compounds 116a–c (Scheme 21). The synthetic approach to 116 was relatively short and efficient, although some steps had moderate yields, and the palladium-catalyzed couplings might raise the cost and environmental concerns. The purity profile was well-controlled, with >95% purity confirmed by HPLC, and cytotoxicity risks were raised by the introduction of cyanovinyl groups, which may form covalent bonds. The compound that showed the most interesting activity was compound 116c, which exhibited high activity against wild-type HIV-1 and various multiresistant mutants, such as L100I, K103N, Y181C, Y188L, E138K, and F227L + V106A, and the double-mutant K103N + Y181C, with lower cytotoxicity and favourable pharmacokinetics.^115^

#### 2.6.3. 2,4,6-Trisubstituted pyrimidines

**Scheme 22:**
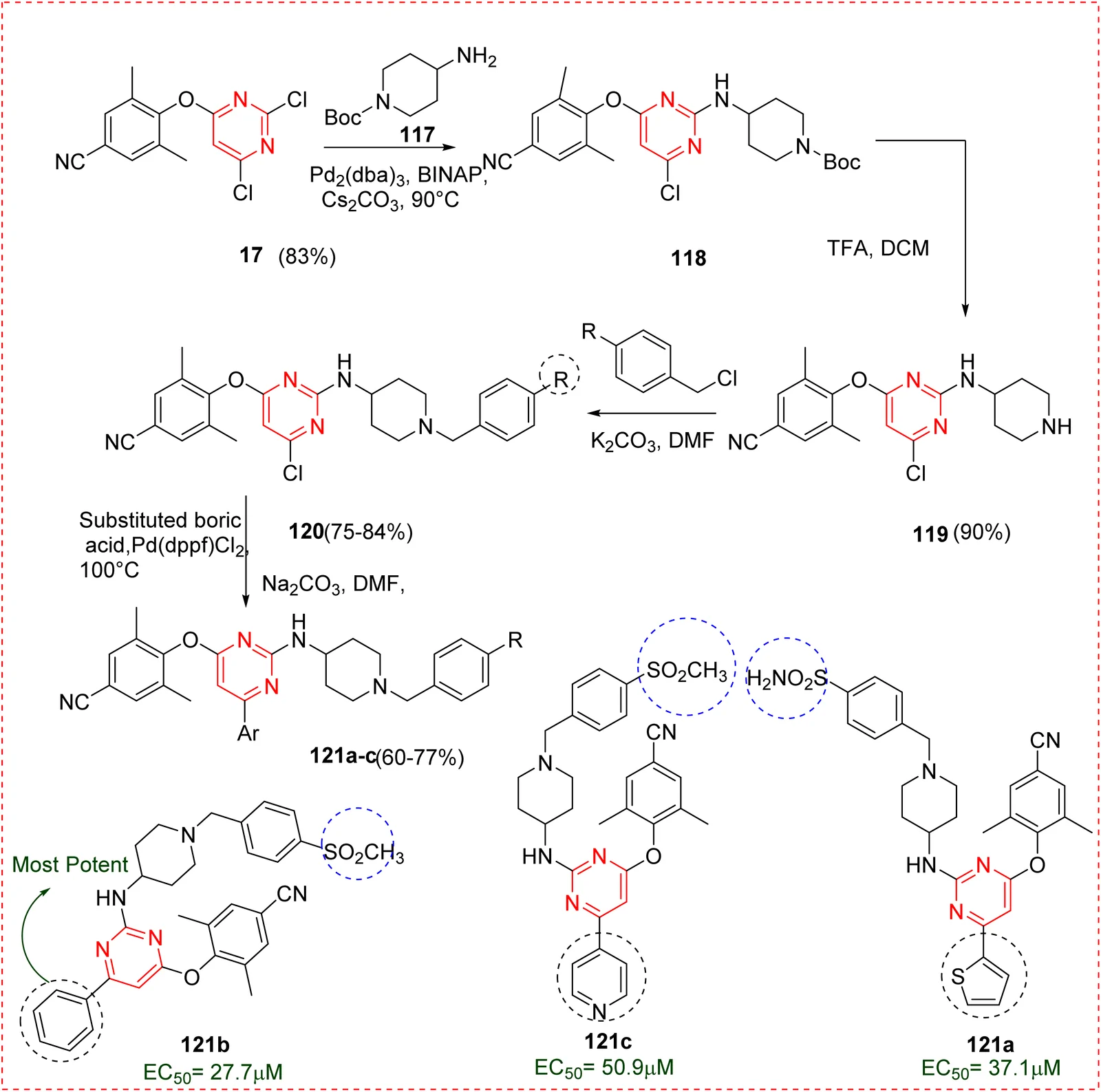
Synthesis of pyrimidine derivatives 121a–c.

The synthesis of 121a–c, which demonstrated strong potency against various mutant HIV-1 strains, was reported by Zhou *et al.* (Scheme 22). The synthesis initiated when previously synthesized 17, upon the Buchwald–Hartwig reaction with Boc-protected piperidine compound 117, generated compound 118, which, upon the removal of the Boc protecting group, yielded compound 119. Upon the reaction of compound 119 with a substituted benzyl chloride, the key intermediate 120 was obtained, which generated key compounds 121a–c*via* the Suzuki reaction with substituted boric acid using Na_2_CO_3_. However, the method afforded moderate yields and required palladium catalysts, resulting in high costs and environmental issues. Impurity profiles were well controlled, HPLC purity > 95%; however, some of the analogs were less active due to structural “active cliffs.” The cost-effectiveness was moderate, as it relied on costly reagents, and environmental safety was adequate, though heavy metal catalysts and halogenated solvents were used. SAR analysis revealed that substituting Ar with phenyl enhanced the activity; thienyl and furyl groups also enhanced the antiviral activity, but not as much as phenyl, while replacing these groups with pyridyl groups reduced the activity. 121c Emerged as the most potent inhibitor of HIV-1 strains. It was active against L100I, K103N, Y181C, E138K, and F227L/V106A, while activity against the double-mutant RES056 was reduced. This indicated that the best group for broad resistance coverage was phenyl.^116^

#### 2.6.4. 2,4,5-Trisubstituted pyrimidine derivatives

**Scheme 23:**
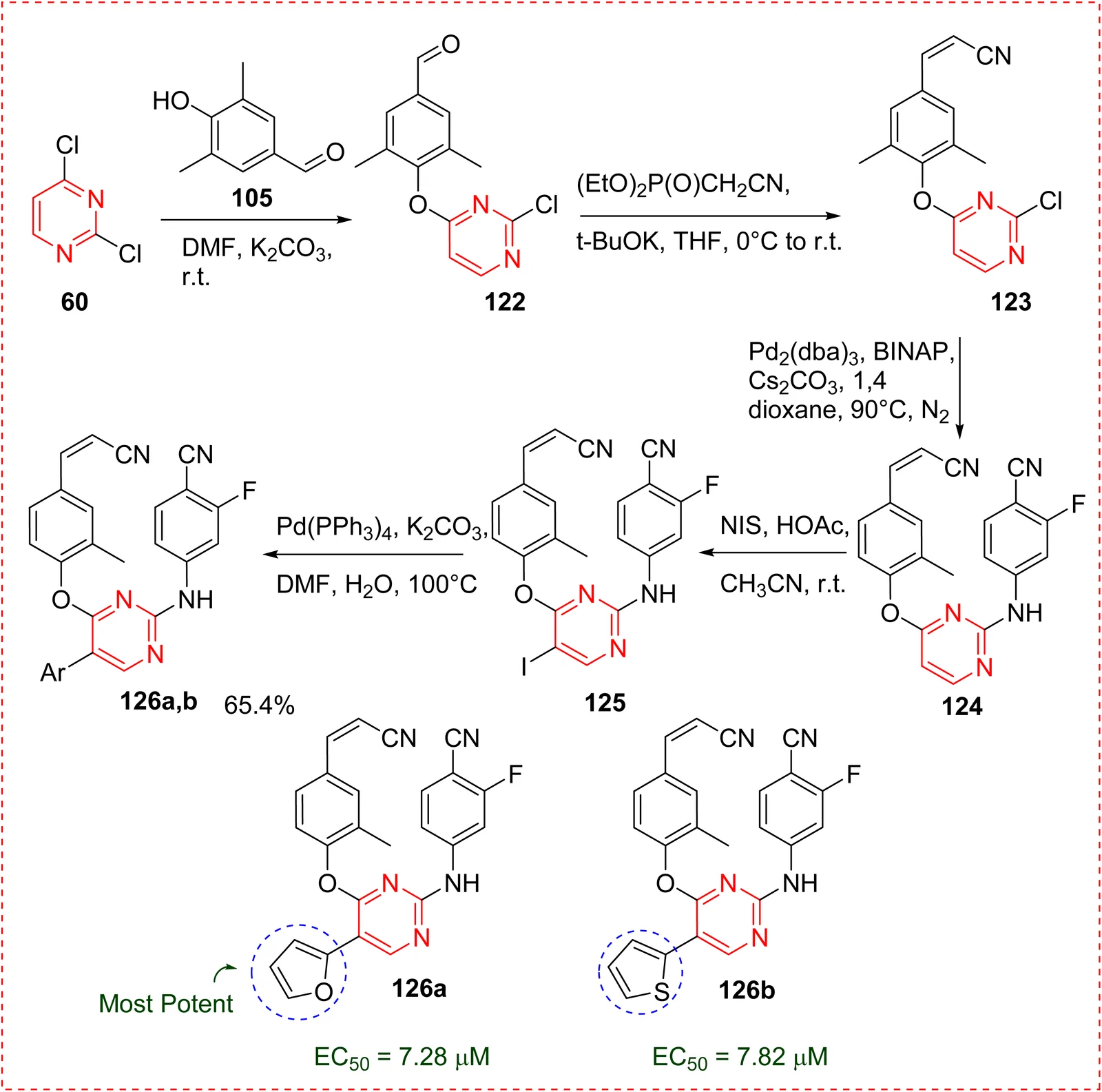
Synthesis of pyrimidine derivatives 126a, b.

Zhou *et al.* were able to synthesise a series of novel compounds (126a, b) that were potent inhibitors of the tolerant region II of the HIV-1 reverse transcriptase (Scheme 23). Synthesis started with commercially available 60, and intermediate 122 was formed by a nucleophilic substitution reaction with 3,5-dimethyl-4-hydroxybenzaldehyde (105) in DMF with the help of K_2_CO_3_ at room temperature. Intermediate 122 reacted with diethyl cyanomethylphosphonate in the presence of *t*-BuOK from 0 °C to room temperature in THF to give intermediate 123. This was followed by intermediate 123, which was cross-coupled with 4-amino-2-fluorobenzonitrile with Pd_2_(dba)_3_, BINAP and CS_2_CO_3_ in 1,4-dioxane at 90 °C under nitrogen gas to give compound 124. In the following step, compound 124, upon iodination with *N*-iodosuccinimide (NIS) and HOAc in CH_3_CN at room temperature, generated intermediate 125. Lastly, intermediate 125 was coupled with various aryl groups by the Suzuki reaction with Pd(PPh_3_)_4_ and K_2_CO_3_ in the mixture of DMF and H_2_O at 100 °C to yield the target compounds (126a, b). This method was effective but afforded moderate yields and exhibited some toxicity features. Palladium catalysts and specialty heterocycles were needed, and the environmental safety was acceptable because the workups were aqueous and no highly toxic reagents were required. In summary, the SAR analysis indicated that increased potency and selectivity of the compounds were achieved by polar substitutions at the tolerant region II, whereas five-membered heterocycles (thiophene and furan) led to better activity against resistant strains. The results of SAR analysis were helpful in optimizing the Ar position by replacing it with five-membered heterocycles to improve the antiviral activity in the tolerant region II. As such, the most active compound was 126a against the wild-type HIV-1 mutants L100I, K103N, Y181C, Y188L, E138K, and RES056.^117^

#### 2.6.5. 2,4,5-Trisubstituted pyrimidine derivatives

**Scheme 24:**
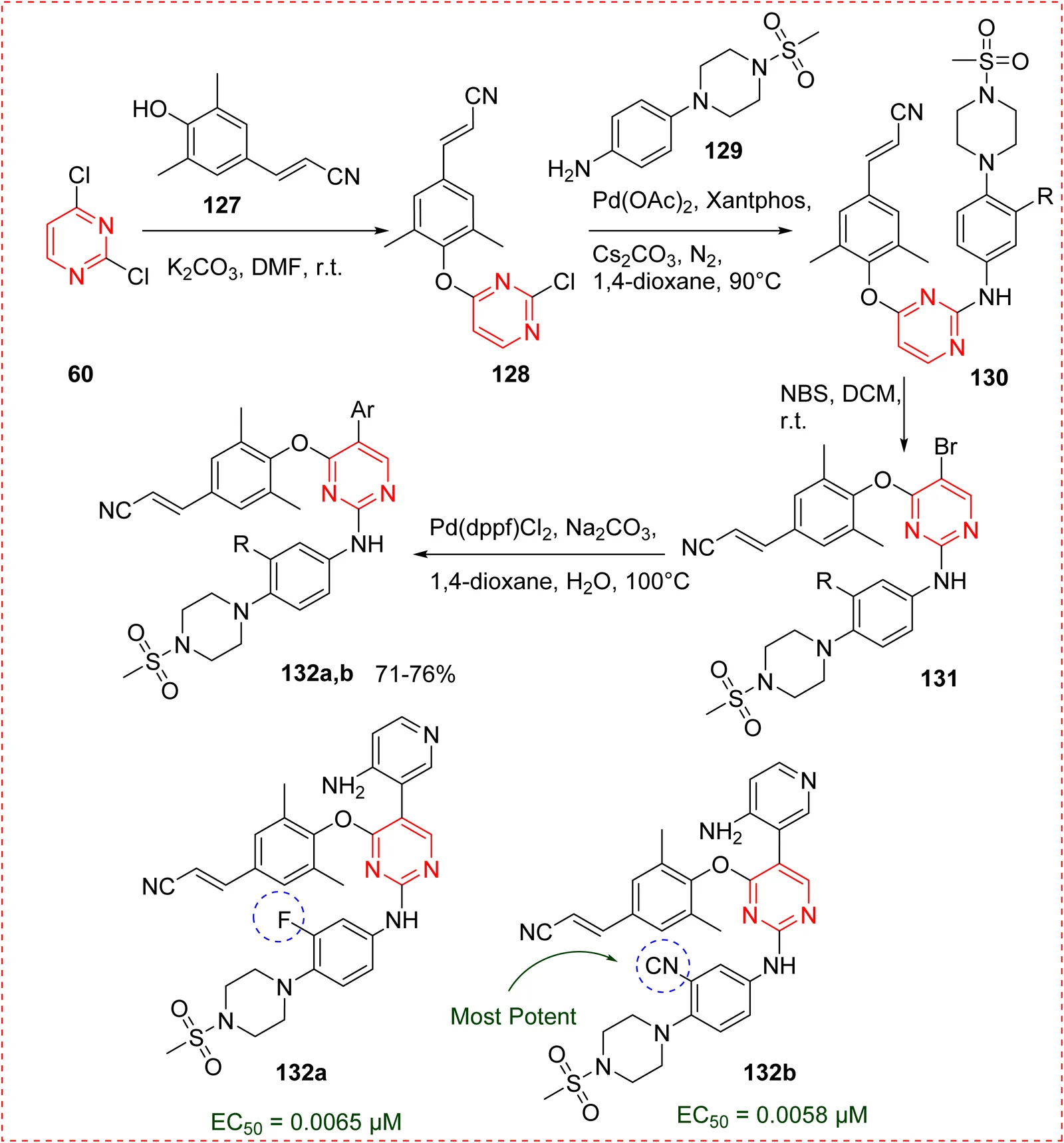
Synthesis of pyrimidine derivatives 132a, b.

Zhou *et al.* synthesized a series of compounds (132a, b), which are highly potent against the wild-type and mutant HIV-1 strains and targeted the tolerant regions of the non-nucleoside reverse transcriptase inhibitor binding pocket (Scheme 24). The synthetic approach started using commercially available 60, which underwent a nucleophilic substitution reaction with 127 at room temperature to give intermediate 128. In the next step, intermediate 128 underwent a Buchwald–Hartwig cross-coupling reaction with substituted 1-(4-aminophenyl)-4-(methylsulfonyl)piperazines (129) to provide intermediate 130, which, upon reacting with *N*-bromosuccinimide (NBS), gave the key brominated compound 131. The final step involved the Suzuki coupling reaction of compound 131 with different arylboronic acids at 100 °C to produce the desired compounds (132a, b) with the help of Pd(dppf)Cl_2_. Preliminary SAR analysis demonstrated that the introduction of certain aromatic structures to the tolerant region II was able to significantly increase the anti-viral activity against resistant mutants. Compound 132b proved to be the most active compound overall, being more active than the rest of the compounds against a panel of difficult single- and double-mutant HIV-1 strains, as well as wild-type HIV-1.^118^

### 2.7. Piperidine-based thiazolo(5,4-*d*)pyrimidines

**Scheme 25:**
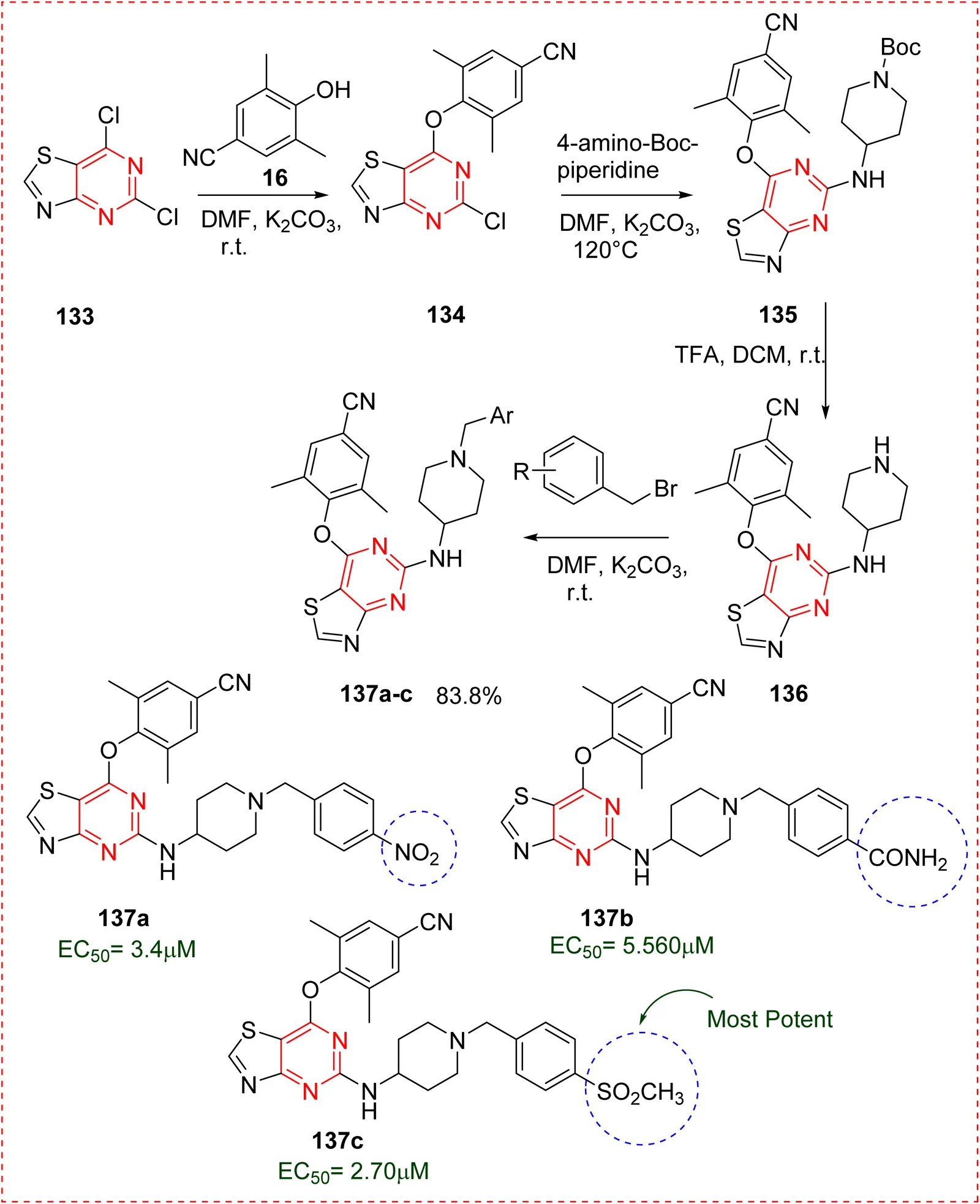
Synthesis of pyrimidine derivatives 137a–c.

The synthesis of thiazolo-based pyrimidine derivatives (137a–c) that demonstrated potent activity against mutant HIV-1 strains was reported by Kang *et al.* (Scheme 25). The synthesis initiated when commercially available dichlorothiazolo pyrimidine (133) reacted with 4-hydroxy-3,5-dimethylbenzonitrile (16), and intermediate 134 was generated. Upon the nucleophilic substitution of 135 with Boc-protected piperidine (135) and by removal of the Boc protecting group, intermediate 136 was obtained. The nucleophilic substitution between 136 and substituted benzyl bromide yielded the target compounds (137a–c). NNRTIs presented some drawbacks, including low aqueous solubility and, in a few cases, cytotoxicity from reactive substituents. Impurity profiles were primarily associated with nitro-substituted derivatives that showed an increase in cytotoxicity. SAR analysis revealed that hydrophilic substitutions at position 4 in the phenyl ring enhanced the antiviral activity, and *para* substitutions were preferred over *meta* substitutions, while nitro substitutions reduced the activity. 137c turned out to be the most potent drug for L100I and K103N + Y181C mutations in HIV-1 with reduced cytotoxicity due to the sulfonyl moiety.^119^

### 2.8. Diarylaminoquinazoline derivatives

**Scheme 26:**
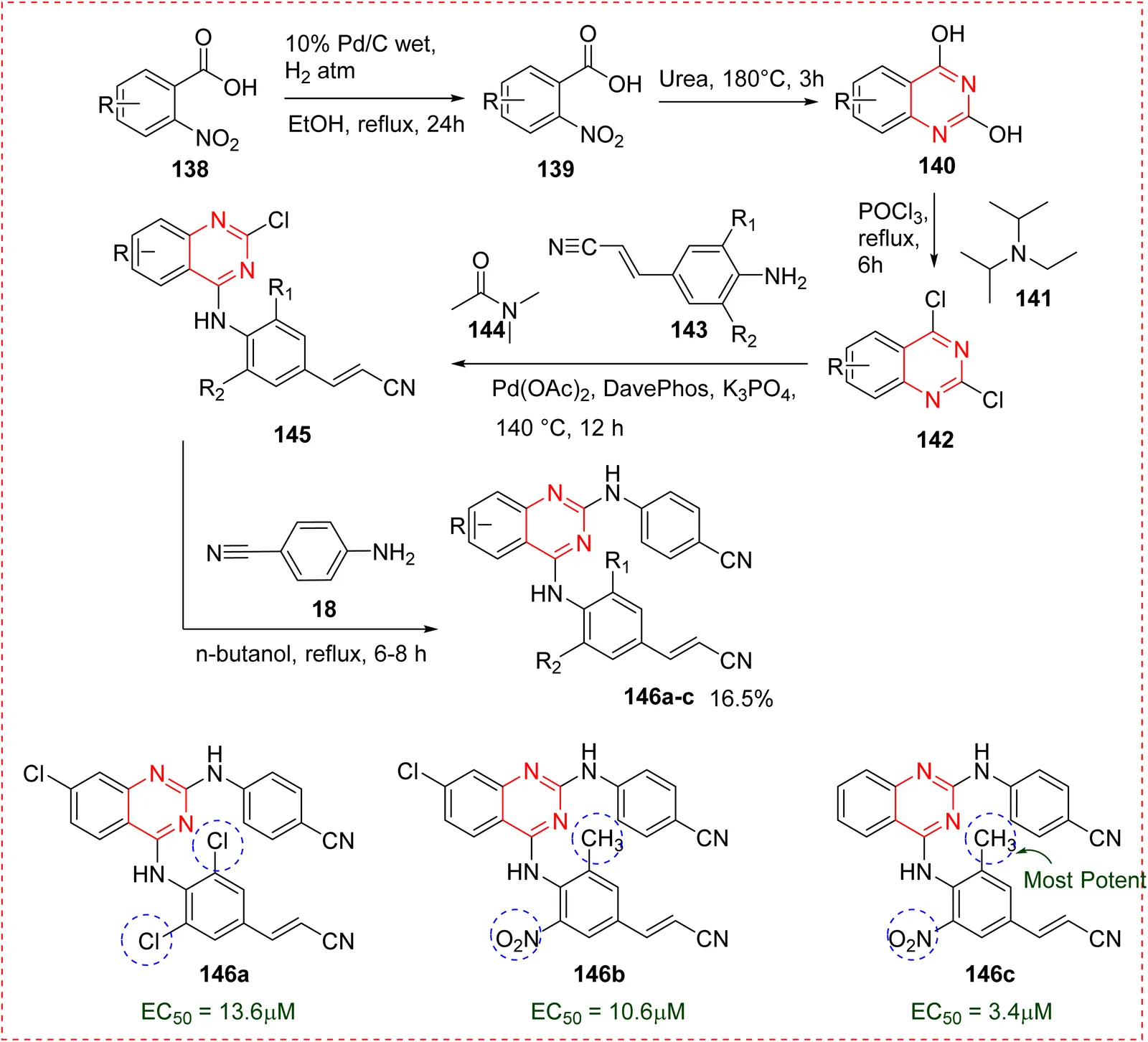
Synthesis of pyrimidine derivatives 146a–c.

Han *et al.* prepared a series of compounds (146a–c) that served as HIV-1 non-nucleoside reverse transcriptase inhibitors (NRTIs) against the wild-type and mutant viruses (Scheme 26). The synthesis started with commercially available substituted 2-nitrobenzoic acids (138), which were reduced in the presence of 10% Pd/C in a H_2_ atmosphere to give anthranilic acids (139). The condensation of 139 with urea at 180 °C gave quinazolinediones (140), which were chlorinated by POCl_3_ and *N*,*N*-diisopropylethylamine (141) to produce 2,4-dichloroquinazoline (142). Next, intermediate 144 was obtained by reacting intermediate 142 with (*E*)-3-(4-amino-3,5-disubstituted)acrylonitriles (143) in the presence of a palladium catalyst (Pd(OAc)_2_, DavePhos). 4-aminobenzonitrile (18) was used for the final amination to obtain the target compounds (146a–c) in refluxing n-butanol. The structural analysis performed using SAR analysis suggested that the use of 2-methyl and 6-nitro substitutions in the phenyl ring on the right wing increased potency. The overall antiviral activity was further enhanced by removing the substituent on the quinazoline core. Compound 146c was found to be the most active compound with excellent activity against WT strains and retained strong efficacy against E138K and K103N mutants.^120^

### 2.9. 5-Alkyl-6-(benzo[*d*][1,3]dioxol-5-alkyl)-2-mercaptopyrimidin-4(3*H*)-ones

**Scheme 27:**
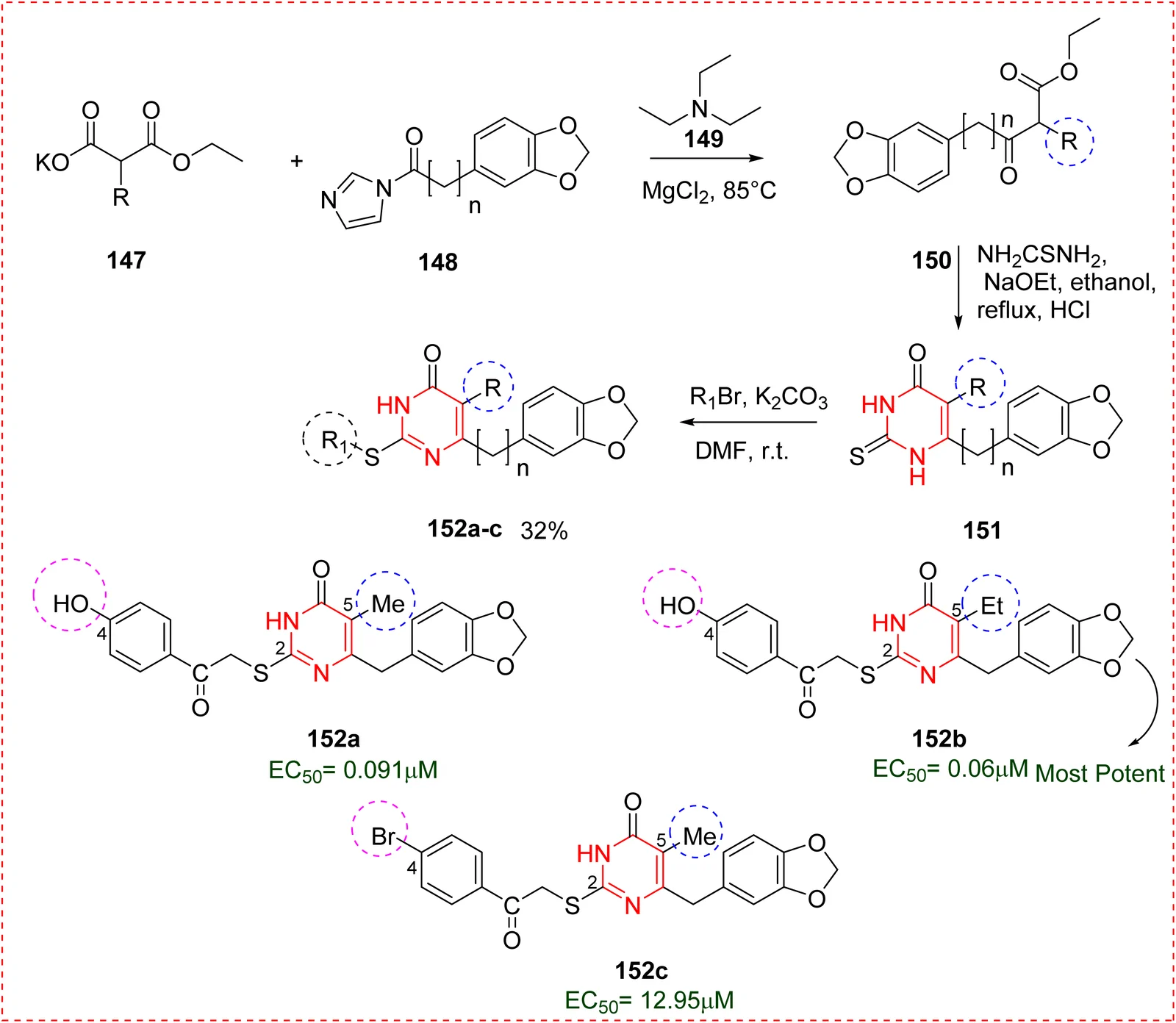
Synthesis of pyrimidine derivatives 152a–c.

Li *et al.* synthesized 151a–c that showed potency against WT HIV-1 strains. The synthesis initiated when commercially available 147 was reacted with compound 148 in the presence of trimethylamine (149) and afforded intermediate 50, which, upon condensation with NH_2_CSNH_2_ in the presence of NaOEt, generated compound 151. Finally, the target compounds 152a–c were obtained upon treating compound 151 with substituted bromine in the presence of potassium carbonate (Scheme 27). SAR analysis revealed that proper substitutions on the pyrimidine ring enhanced the activity. The positions such as carbon 2 and carbon 5 at R_1_ of the pyrimidine ring are important, and upon substituting the R at carbon 5 with methyl, the activity was enhanced, but it was further enhanced upon replacing methyl with the ethyl group. Substituting R_1_ with PhCOCH_3_ enhanced the antiviral activity, but adding a hydroxyl group at the 4th position of the phenyl group resulted in a maximum increase in activity, while replacing it with bromine reduced the activity. 152b emerged as the most active against WT HIV-1 strains, while NNRTI-resistant mutants K103N and Y181C were weakly inhibited.^121^

### 2.10. Piperidinyl-substituted [1,2,4]triazolo[1,5-*a*]Pyrimidines

**Scheme 28:**
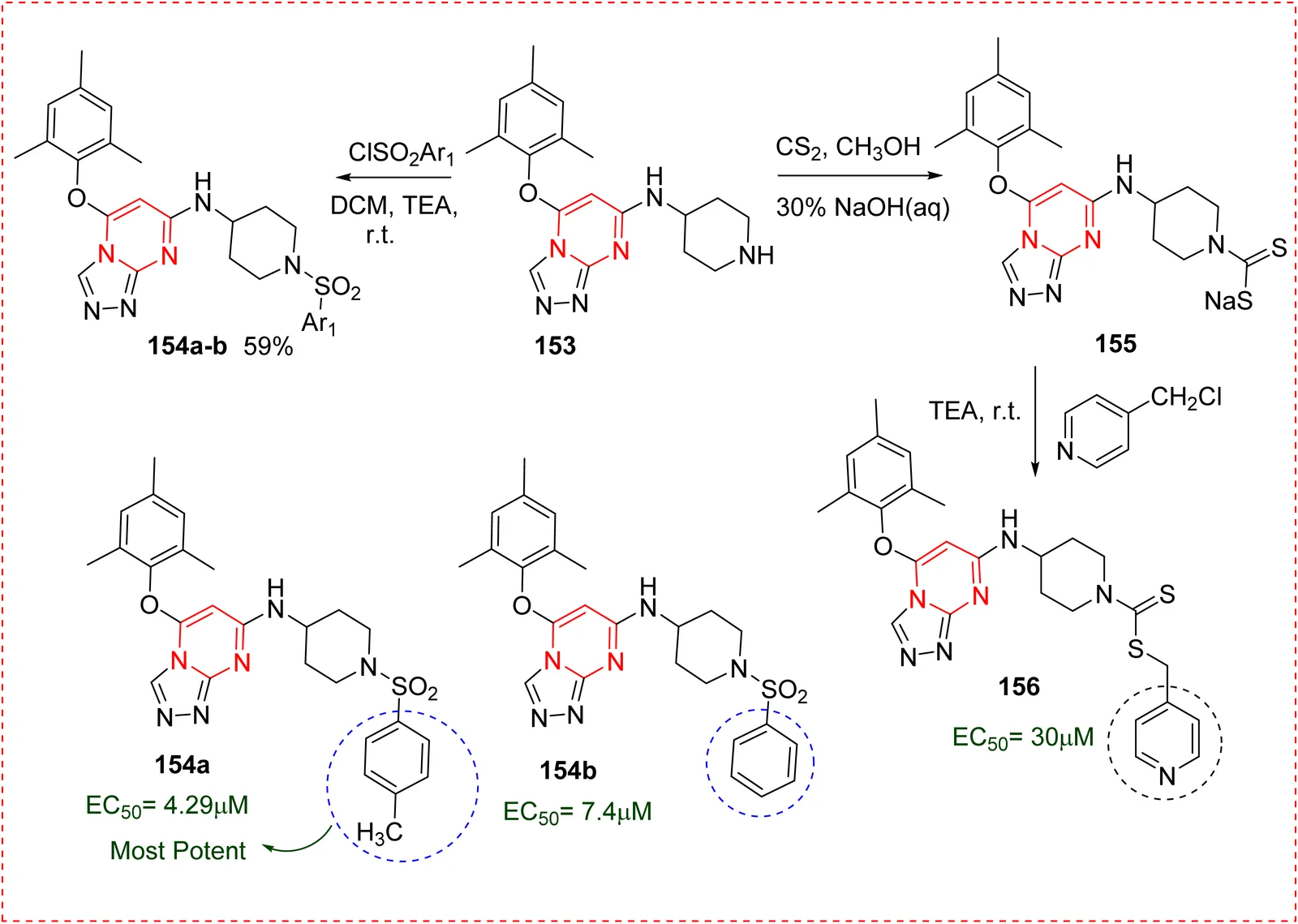
Synthesis of pyrimidine derivatives 154a, b.

Huang *et al.* synthesized 154a, b that demonstrated potency against the HIV-1 IIIB strain (Scheme 28). The synthesis initiated when compound 153 was reacted with chlorides containing sulphonyl to generate key compounds (154a, b). In the next step, compound 153, upon reacting with carbon disulphide using 30% sodium hydroxide solution, afforded intermediate 155, which, upon reacting with chloromethylated pyridine in the presence of TEA, generated another target compound 156. The synthetic strategies to 154a, b were constrained by moderate yields as well as the requirement of a sulfonyl spacer or a dithiocarbamate spacer, sometimes decreasing the potency. The reactions were vigorous under conventional laboratory conditions, and the lack of cost-effectiveness was due to the use of specialty reagents and multistep synthesis. These compounds were active against HIV-1 wild-type (IIIB) but inactive against the HIV-1 K103N + Y181C double-mutant and HIV-2 strains, thus exhibiting mutant selectivity limitations. 54a emerged as the most potent against the HIV-1 IIIB strain.^122^

### 2.11. Dihydrothiopyrano pyrimidines

#### 2.11.1. Dihydrothiopyrano[4,3-*d*] pyrimidine derivatives

**Scheme 29:**
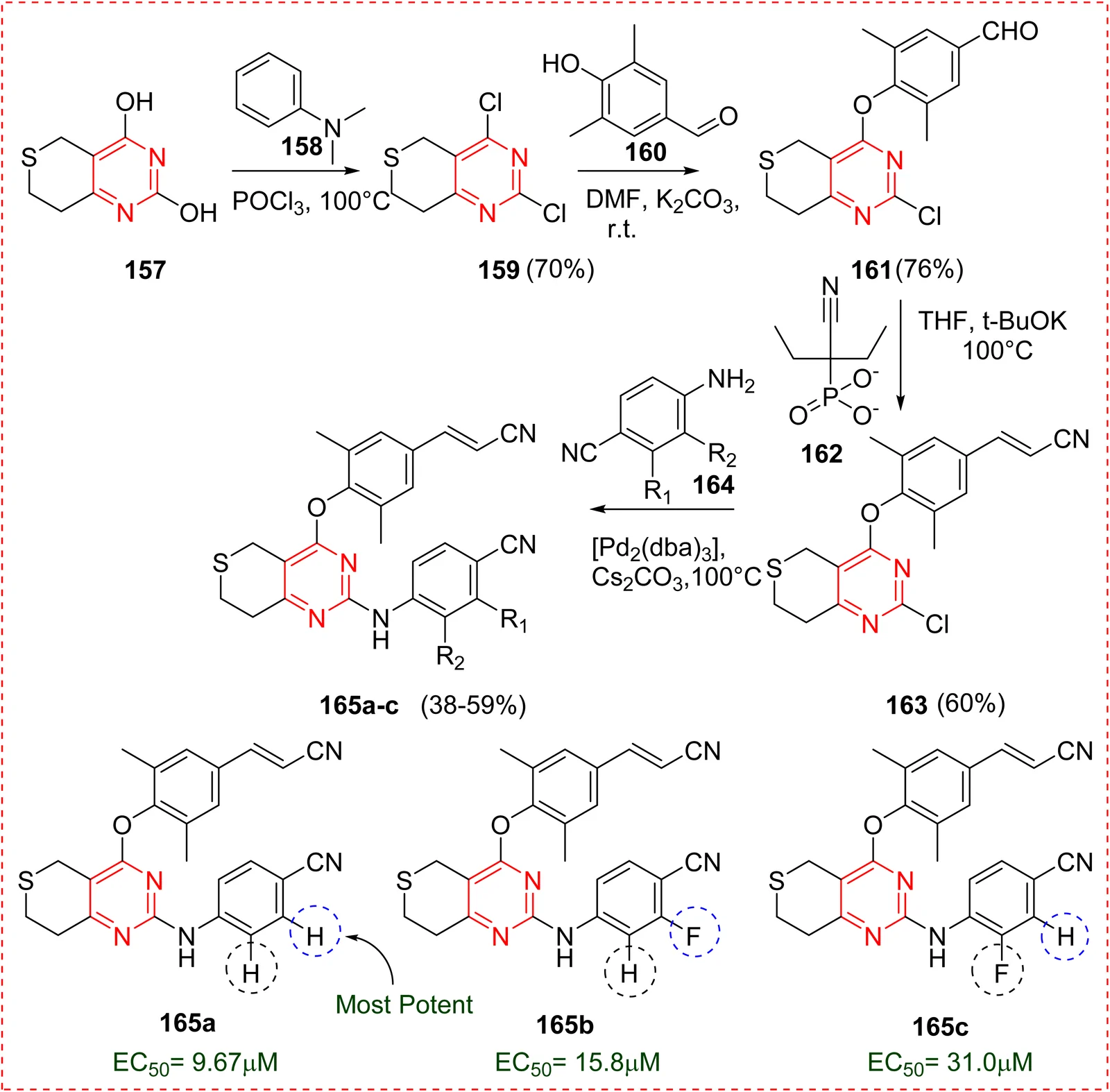
Synthesis of pyrimidine derivatives 165a–c.

Wang *et al.* reported the synthesis of thiopyrano pyrimidine derivatives (65), which demonstrated strong antiviral potency against different resistant strains of HIV-1. The synthesis initiated when the commercially available dihydrothiopyrano derivative 157, upon chlorination with POCl_3_ in the presence of *N*,*N*-dimethylaniline (158), generated compound 159. In the next step, compound 160, upon reacting with aldehyde 161, yielded the key intermediate 162. Upon the Wittig–Horner reaction with diethylcyanomethylphosphonate (163) using trifluoroacetic acid (TFA), compound 161 afforded intermediate 163. Upon undergoing the Buchwald–Hartwig reaction with different aminobenzonitriles (164), compound 163 generated the target compounds 165a–c (Scheme 29). Synthetic pathways to 165a–c NNRTIs were efficient, but produced moderate yields and were multi-step. The impurity profiles were clean, although some lipophilic substituents were sometimes added, causing cytotoxicity or metabolic instability. Later analogues, notably compound 165a, demonstrated a higher degree of robustness as they were potent against both wild-type and resistant HIV-1 strains. SAR analysis revealed that the phenyl ring without any substitutions enhanced the antiviral activity, but substituting it with a fluorine or nitrogen atom at the R_1_ position reduced the activity, and the activity was further reduced when the R_2_ position was substituted with a fluorine or nitrogen atom. 165a emerged as the most potent against WT HIV-1 IIIB L100I, K103N, Y181C, E138K and Y188L mutants.^123^

#### 2.11.2. Dihydrothiopyrano[3,2-*d*]pyrimidine scaffolds

**Scheme 30:**
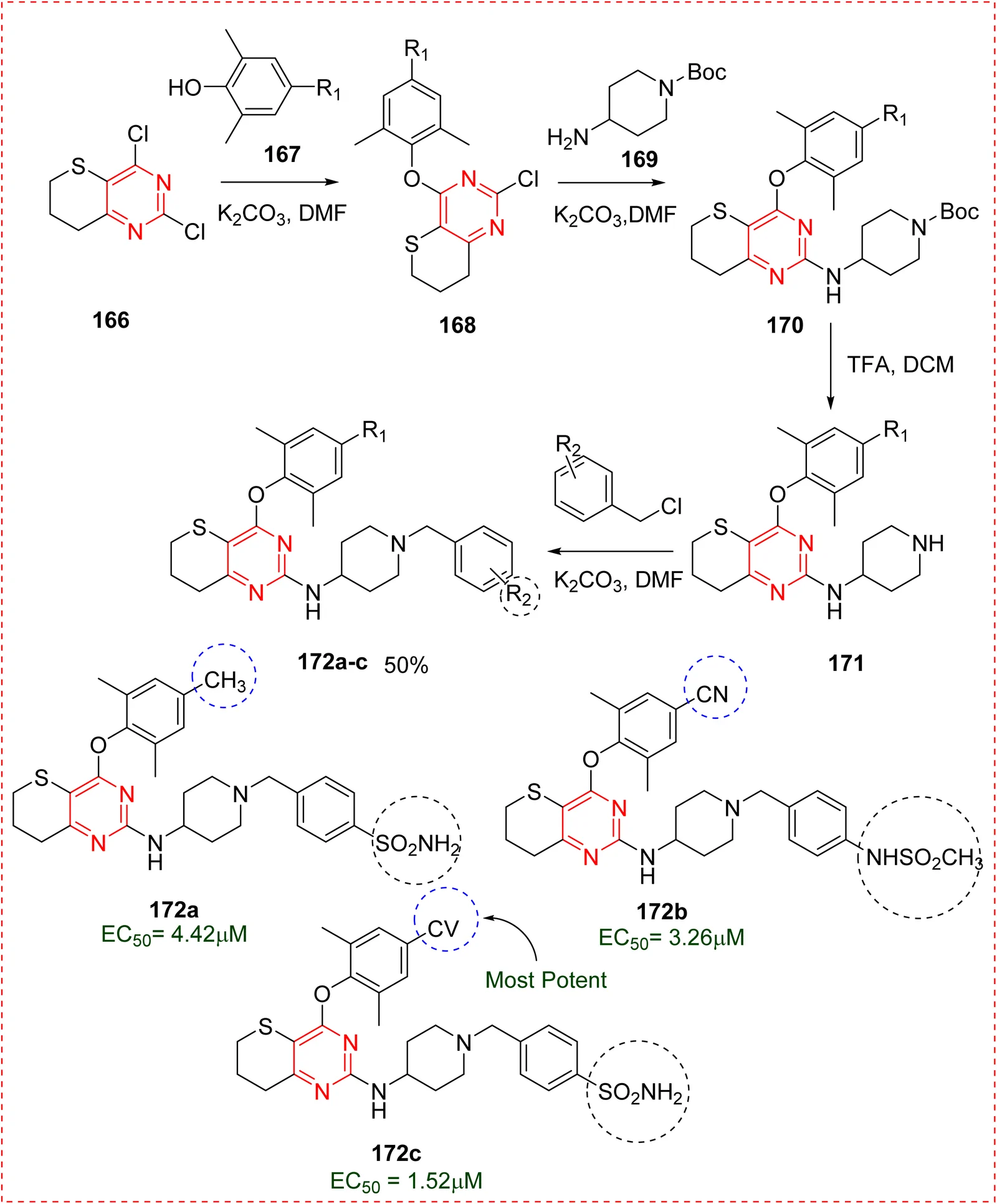
Synthesis of pyrimidine derivatives 172a–c.

Wang *et al.* reported the synthesis of thiopyrano-based pyrimidine derivatives 172a–c that showed excellent activity against the resistant strains of HIV-1 (Scheme 30). 172c emerged as the most potent against the resistant HIV-1 strain RES056, which carries the single mutations. The synthesis initiated when commercially available di-chloro thiopyrano pyrimidine (166), upon nucleophilic substitution with different phenols (167), afforded intermediate 168. In the next step, intermediate 168 reacted with Boc-protected piperidine (169) to yield intermediate 170, which, upon the removal of the Boc protecting group, generated the key intermediate 171. Upon a nucleophilic reaction with different benzyl chlorides, the target compounds (172a–c) were obtained. The reactions were sufficient to produce a variety of analogues, but practical production on a large scale was not mentioned, so current practicality is limited to preclinical synthesis. Cost-effectiveness was moderate, with the palladium-catalyzed steps and the presence of specialized intermediates being expensive, but the overall yields were still acceptable. The structure–activity relationship (SAR) analysis revealed that the substitution of the cyanovinyl (CV) group at R_1_ enhanced the potency more than the substitution of nitrile and methyl groups. 172c was active against L100I, K103N, Y181C, Y188L, E138K and the substantially more resistant double mutants F227L + V106A and K103N + Y181C.^124^

### 2.12. Piperidin-4-yl-aminopyrimidines

#### 2.12.1. Indazolyl-substituted piperidin-4-yl-aminopyrimidines

**Scheme 31:**
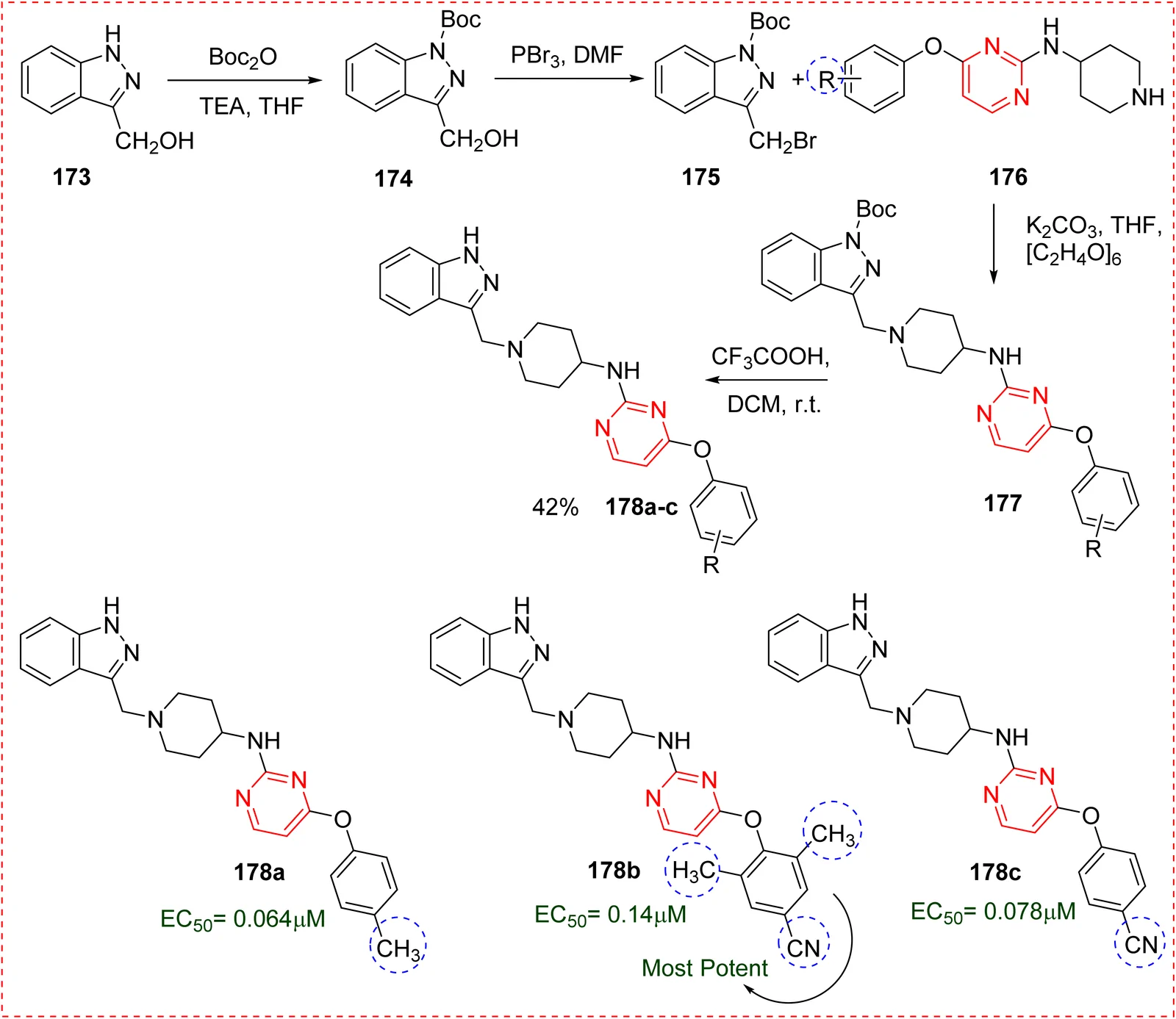
Synthesis of pyrimidine derivatives 178a–c.

Xiao *et al.* synthesized 178a–c that showed strong potency against wild-type HIV-1 strains (Scheme 31). The synthesis initiated when commercially available 173 reacted with di-*tert*-butyl dicarbonate in the presence of THF to generate compound 174. Upon bromination with PBr3, compound 174 afforded compound 175, which, upon reacting with compound 176 in the presence of K_2_CO_3_, generated the key intermediate 177. Upon removing the Boc protecting group of the key intermediate 177 in the presence of CF_3_COOH and dichloromethane (DCM), the target compounds (178a–c) were obtained. SAR analysis revealed that substituting the phenyl wing with an electron-donating group/electron-withdrawing group at the *para* position enhanced the antiviral activity more than a *meta*-substituent; furthermore, substituting this phenyl wing with methyl groups at *ortho* positions further enhanced the antiviral activity. 178b emerged as the most active against the HIV-1 IIIB strain. It had high activity against resistant mutants K103N, Y181C, and E138K, while RES056, the double mutant, was moderately resistant, demonstrating its resistance against clinically relevant mutations.^125^

#### 2.12.2. 2,4-Disubstituted pyrimidine derivatives

**Scheme 32:**
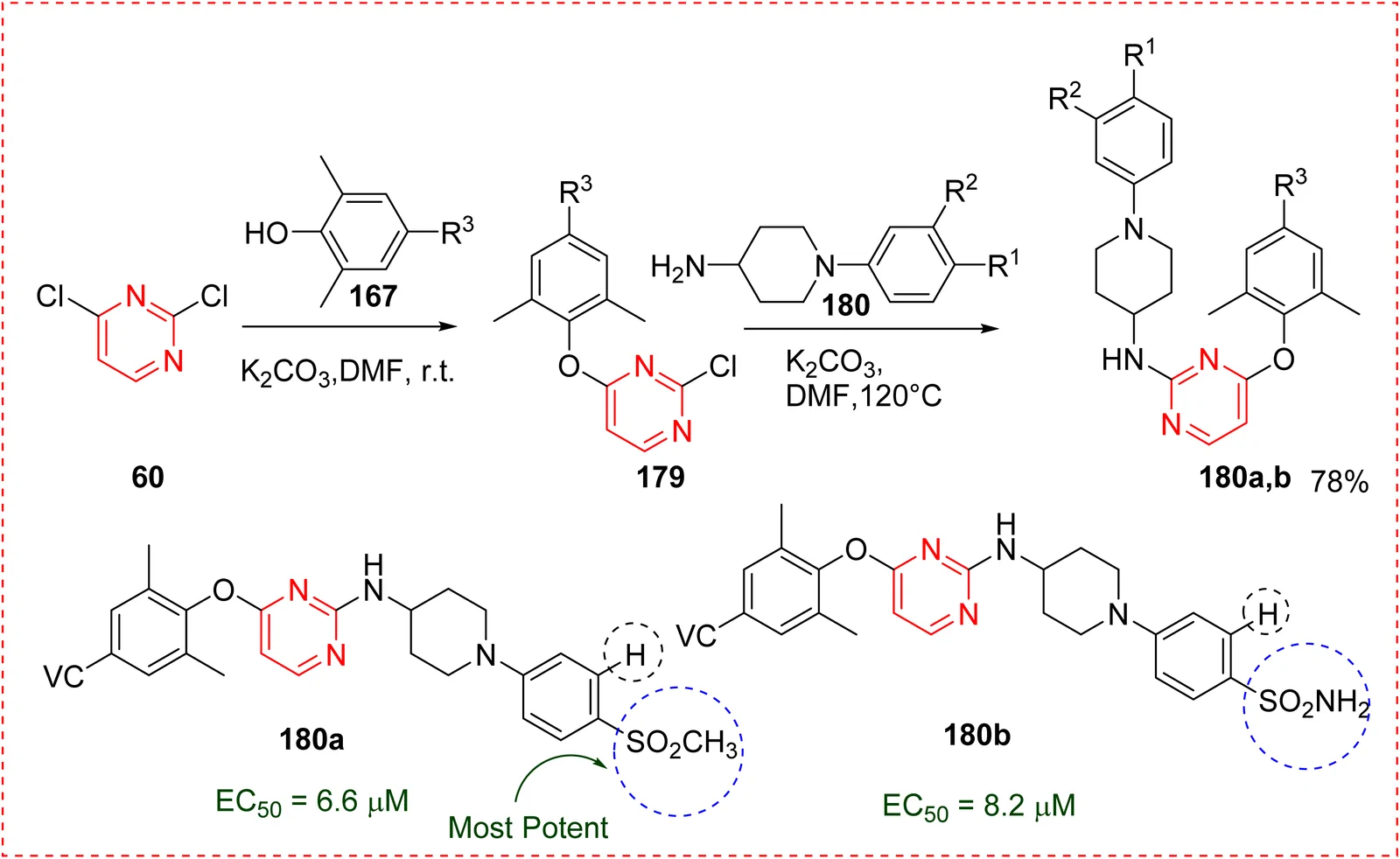
Synthesis of pyrimidine derivatives 180a, b.

For HIV-1 non-nucleoside reverse transcriptase inhibitors (NNRTIs) that have been reported to be prone to drug resistance issues, Xie *et al.* designed and synthesized a series of novel compounds (180a, b) to enhance their metabolic stability. Commercial 2,4-dichloropyrimidine (60) was used as the starting material, and it was nucleophilically substituted by substituted 4-hydroxy-3,5-dimethylbenzenes (167) in the presence of K_2_CO_3_ and DMF at room temperature to give intermediate 179. Finally, intermediate 179 was heated with different 4-(piperidin-1-yl) aniline derivatives (180) in DMF in the presence of potassium carbonate to produce the desired diarylpyrimidine derivatives (180a, b) (Scheme 32). Substituting *R*^3^ with a cyano-vinyl (CV) group was found to significantly improve the antiviral activities compared to a cyano (CN) group, according to SAR analysis. In addition, the optimization of the right-wing piperidine moiety showed that the presence of hydrogen-bond-donating and -accepting groups affected the potency, and the order of activities was found to be as follows: SO_2_CH_3_ > CONH_2_ > SO_2_NH_2_ > *m*-NO_2_ > *p*-NO_2_. Compound 180a proved to be the most active inhibitor in this series. It also had high activity against a broad panel of single-mutant strains (including K103N, L1001, and Y181C) and was very active against the highly resistant double-mutant strain F227L + V106A.^126^

#### 2.12.3. Hybrids of delavirdine and piperidin-4-yl-aminopyrimidines

**Scheme 33:**
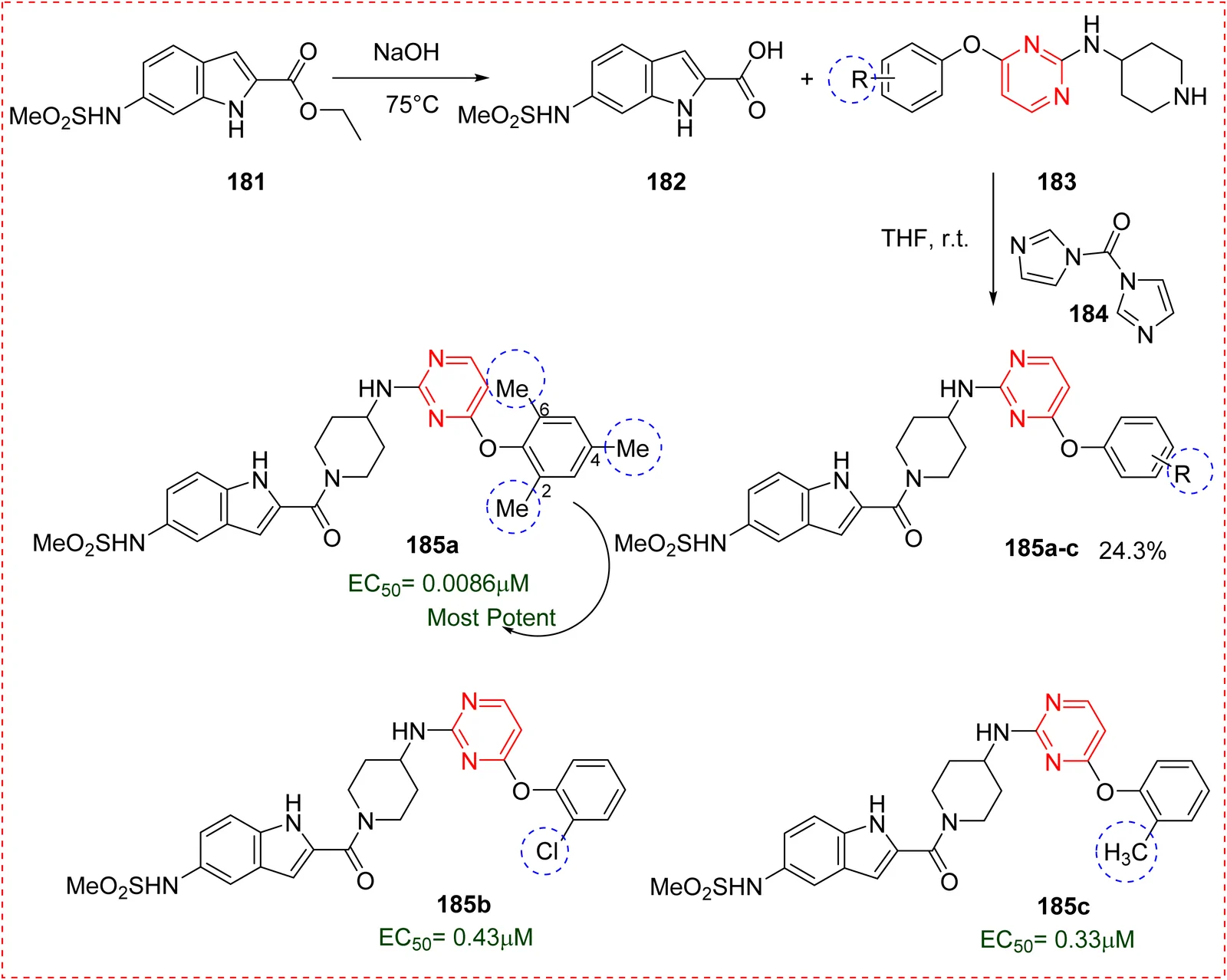
Synthesis of pyrimidine derivatives 185a–c.

The synthesis of delavirdine and piperidin-4-yl-aminopyrimidine hybrids (185a, b), which showed moderate potency against the wild-type HIV-1 strain and strong potency against single mutations in HIV-1, was reported by Ming *et al.* (Scheme 33). The synthesis initiated when the commercially available indole derivative 181 reacted with a base to generate compound 182, which, upon reacting with 183 in the presence of *N*,*N*-carbonyldiimidazole (184), yielded target hybrids (185a, b). Most of the impurities were purified by column chromatography, although yields were not always satisfactory, and some were controlled carefully to prevent the formation of side products. SAR analysis revealed that the substitutions of halogens or electron-donating groups at the *ortho* position of the left phenyl ring enhanced the antiviral activity more than *para* or *meta* substitutions. Substituting the phenyl ring with methyl groups at the 2,4 and 6 positions achieved excellent anti-viral activity. 185a emerged as the most potent against the WT HIV-1 IIIB strain and exhibited activity against critical drug-resistant mutants, such as L100I, K103N, Y181C, Y188L, E138K and the double-mutant F227L + V106A, demonstrating its resistance to resistance.^127^

### 2.13. Benzo[2,3-*d*]pyrimidines

**Scheme 34:**
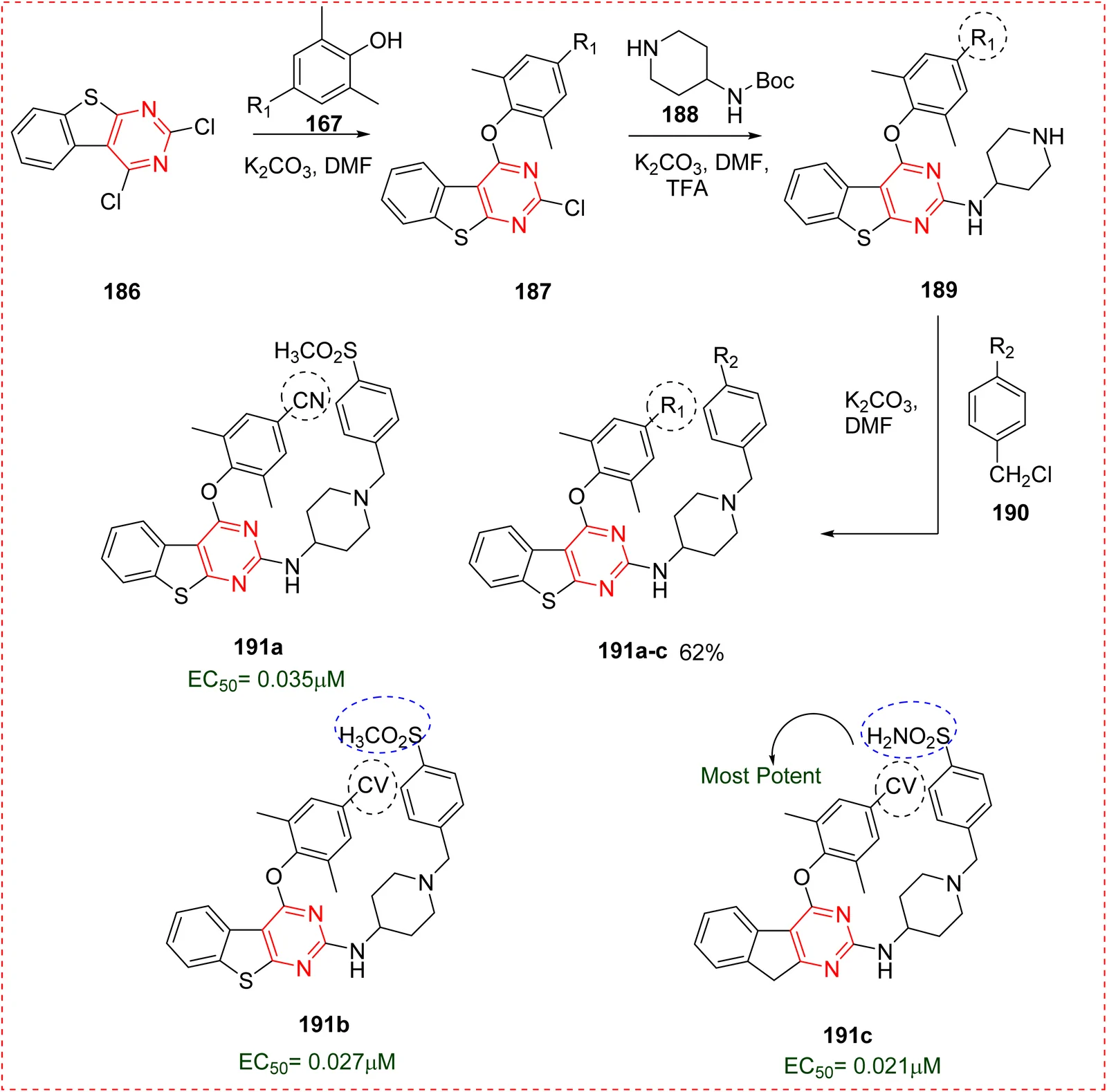
Synthesis of pyrimidine derivatives 191a–c.

The synthesis of 95a–c, which demonstrated excellent activity against mutations in HIV-1, was reported by Meng *et al.* The synthesis proceeded through commercially available 186, upon a substitution reaction with cyanovinyl- or nitrile-substituted dimethyl hydroxylbenzene (167), generating compound 187. Upon the reaction of compound 187 with Boc-protected aminopiperidine (188), followed by deprotection using TFA, compound 189 was obtained. In the final step, compound 189 reacted with substituted benzyl chloride (190) to afford the target compounds 191a–c (Scheme 34). SAR analysis revealed that by substituting R_1_ with nitrile or cyanovinyl, the activity was enhanced, but it was more enhanced by cyanovinyl (CV) substitutions, and replacing R_2_ with sulphonamide further enhanced the antiviral activity. 191c emerged as the most potent against the various mutants of HIV-1 (L100I, K103N, Y181C, Y188L, E138K and F227L/V106A).^128^

### 2.14. Thiazolone[3,2-*a*]pyrimidines

**Scheme 35:**
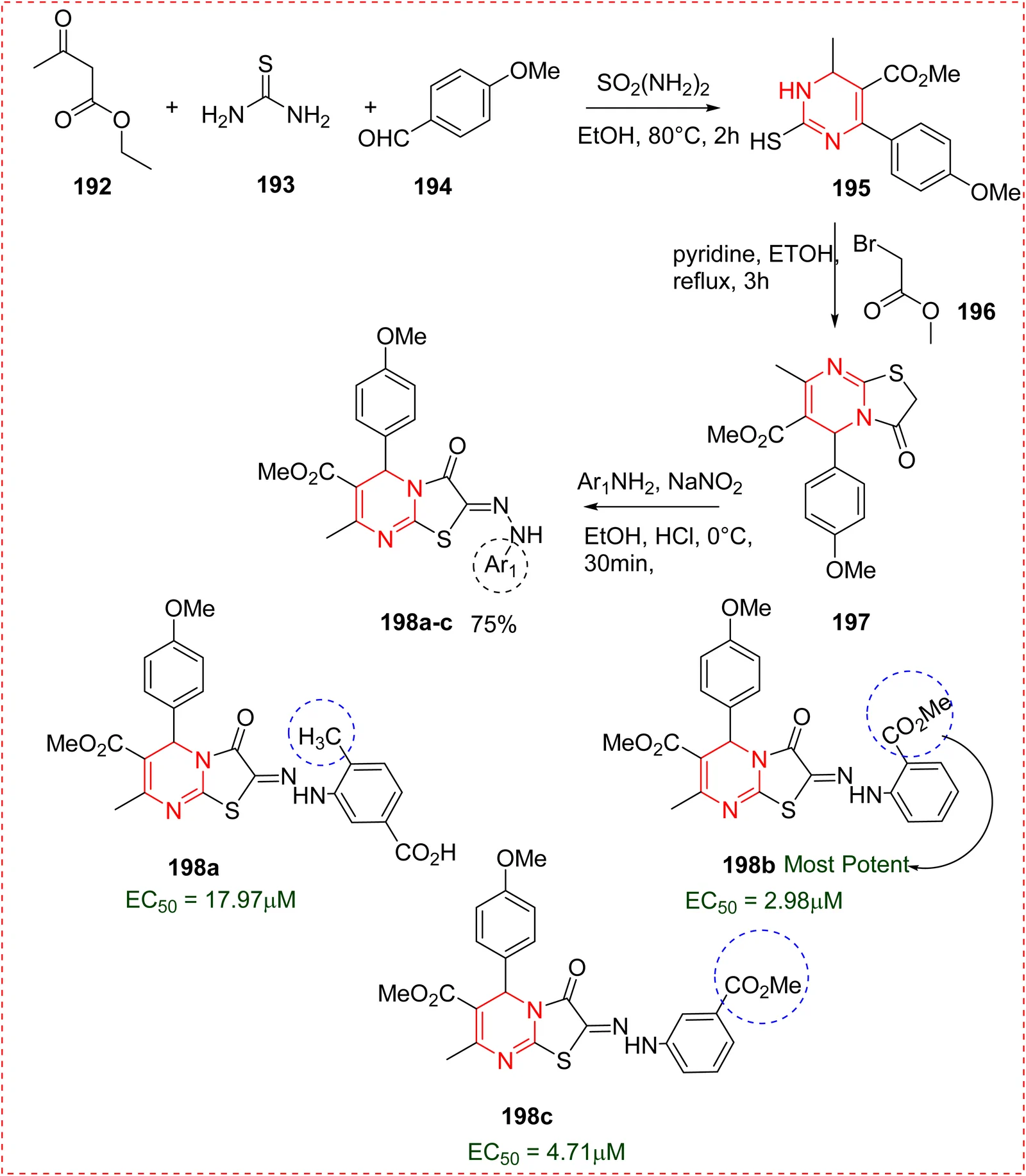
Synthesis of pyrimidine derivatives 198a–c.

Zhu *et al.* reported the synthesis of 198a–c, which demonstrated strong potency against HIV-1 ribonuclease H (RNase H; Scheme 35). The synthesis proceeded as commercially available ethyl acetoacetate (192) reacted with 193 and methoxy benzaldehyde (194) to generate compound 195. In the next step, compound 195 reacted with methyl bromoacetate to afford compound 197, which then reacted with various aromatic diazonium salts at 0 °C. The reaction proceeded for two hours at room temperature to yield the final target compounds 198a–c through a Biginelli-based method and diazonium coupling, which were obtained in moderate yields (30–90%), and diazonium intermediates needed to be handled with care, limiting the scalability. The impurity profile primarily consisted of the side products of incomplete diazotization or overreaction; HPLC was used to ensure acceptable purity. The cost-effectiveness was moderate because common reagents were employed, but purification steps were added to the process, which increased the cost.

SAR analysis revealed that polar substitutions at position 3 of the aryl group enhanced the activity, while substituting at position 2 with these groups further enhanced the antiviral activity, and nonpolar substitutions, such as methyl groups, reduced the activity. 198b turned out to be the most potent inhibitor of ribonuclease H.^129^

### 2.15. *N*-1-Substituted pyrimidines

**Scheme 36:**
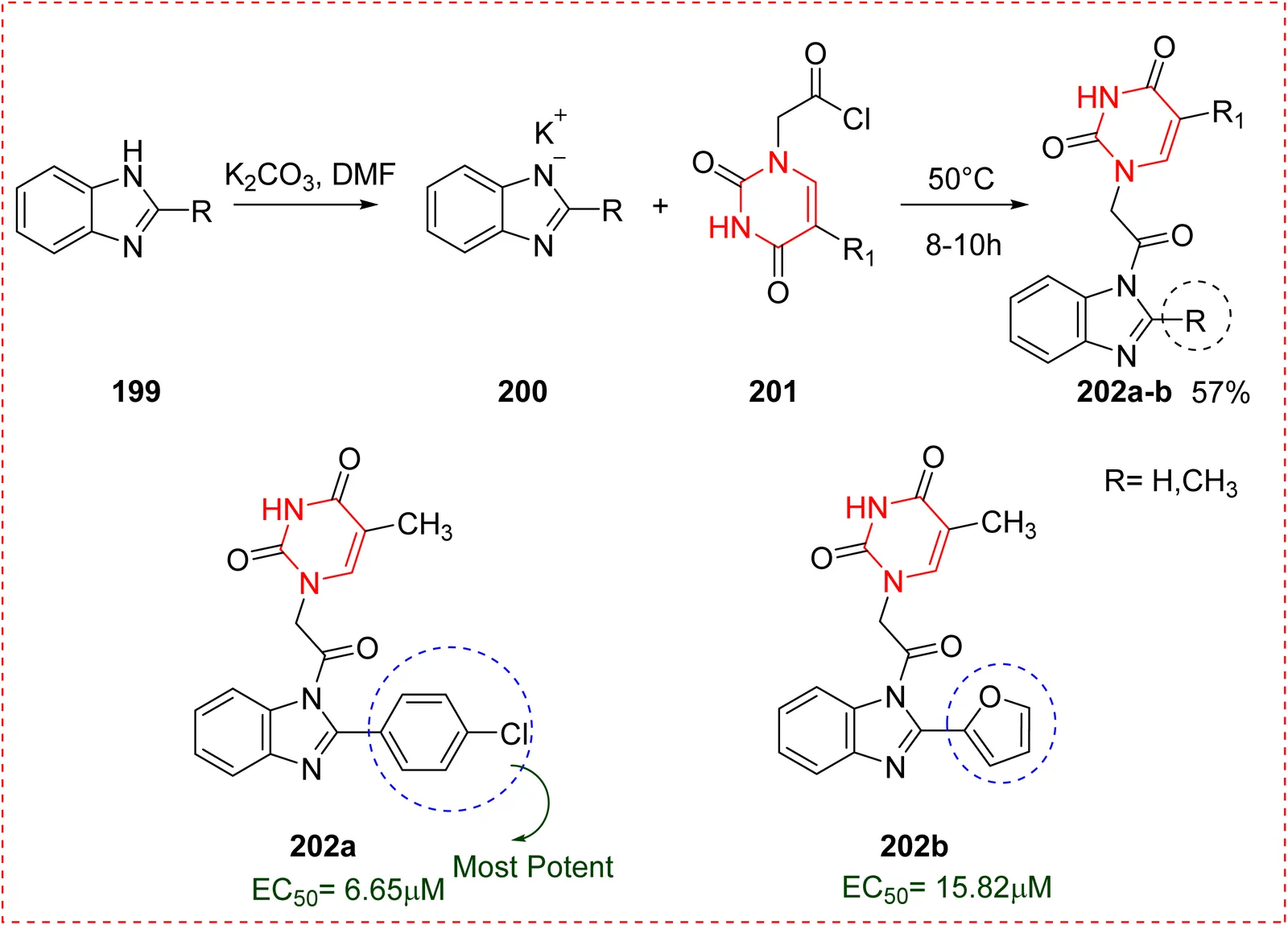
Synthesis of pyrimidine derivatives 202a, b.

Srivastava *et al.* synthesized *N*-1 substituted pyrimidines, which demonstrated activity against viruses such as Coxsackie virus and against HIV-1. The synthesis initiated when commercially available 2-aryl substituted benzimidazoles (199) reacted with thymine/uracil acetic acid (200, 201) in the presence of K_2_CO_3_ to generate the target compounds 202a–c (Scheme 36). 202a was prepared by a simple two-step synthesis, which had moderate yields (∼57%) and required column chromatography for purification, limiting the synthesis to the laboratory scale. The principal byproducts of the impurities were unreacted starting materials and residues of incomplete acylation, which were easily separated by silica gel chromatography. The reaction was very efficient under controlled conditions but only moderately efficient at a larger scale; the use of oxalyl chloride and DMF created hazards, but the reaction was relatively easy to reproduce and was moderately cost-effective. SAR analysis revealed that substituting the R of benzimidazole with a chlorophenyl group enhanced the antiviral activity more than a furyl ring. 202a emerged as the most potent against HIV-1.^130^

### 2.16. Furo[2,3-*d*]pyrimidine derivatives

**Scheme 37:**
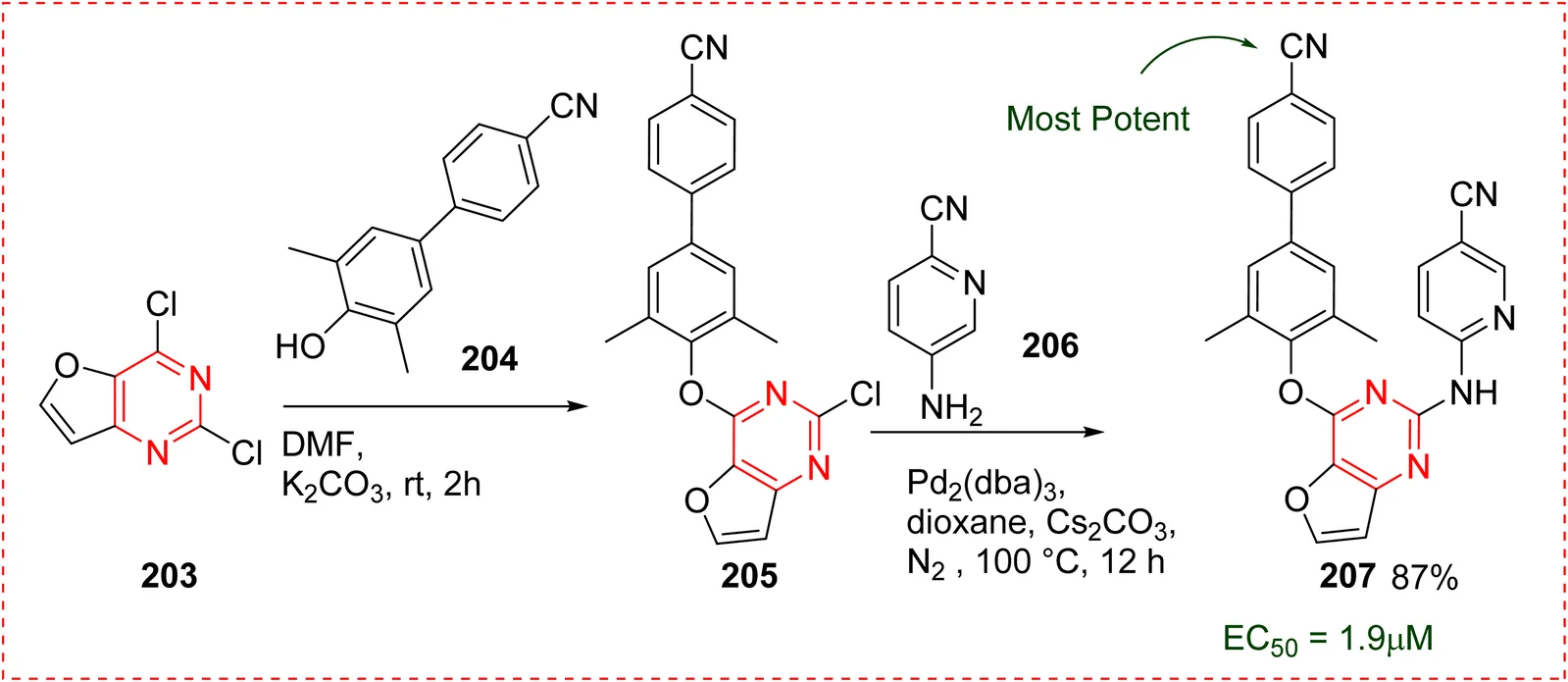
Synthesis of pyrimidine derivative 207.

Zhang *et al.* prepared a novel compound (207) with antimicrobial activity against HIV-1, which served as a non-nucleoside reverse transcriptase inhibitor (NNRTI) (Scheme 37). The synthesis was commenced with commercially available 2,4-dichlorofuro[3,2-*d*]pyrimidine (203), which underwent nucleophilic substitution to give intermediate 205 with (204) and potassium carbonate in DMF. The target compound (207) was obtained in the next step by the Buchwald–Hartwig cross-coupling of 205 with 6-aminonicotinonitrile (206) in the presence of Pd_2_(dba)_3_ and Cs_2_CO_3_ at 100 °C in dioxane. The replacement of the right-wing benzene ring with the modified *p*-cyanopyridylaniline moiety caused a marked improvement in the antiviral activity, increased the selectivity index and reduced the hERG potassium channel liability, as revealed by SAR analysis. Compound 197 proved to be the most active compound against the wild-type HIV-1 and was highly resistant to a number of NNRTI-resistant strains, such as the common single mutant K103N and the highly resistant double mutant F227L + V106A (Table 1).^131^

**Table 1:** Consolidated summary of potent compounds as strong anti-HIV agents

Sr. No.	Potent derivatives	EC_50_ (µM)	Resistant HIV-1 strains	Yield
1	8b	0.125	K103N and Y181C	80–90%
2	14b	6.19	K103N and Y181C	16.5%
3	21	0.281	K103N and Y181C	70.4%
4	26a	0.212	K103N and Y181C	70.4%
5	31	0.01	K103N and E138K	65%
6	36a	13.5	K103N, L100I, Y181C and E138K	80%
7	42c	10.5	L1001, K103N and Y181C	74%
8	47	11.23	K103N, E138K and L100I and the very resistant F227L, V106A	49%
9	52c	0.031	K103N, E138K, Y181C, Y188L, L100I and F227L/V106A	95%
10	56c	0.01	Y181C, Y188L, and F227L + V106A	89%
11	59a	1	L1001, K103N, Y181C, Y188L, E138K, and K103N/Y181C	40–46%
12	66a	0.0035	E138K and K103N	44%
13	72b	0.013	K103N	66.4%
14	76a	0.974	F227L + V106A	97%
15	82a	0.012	E138K, L100I, K103N, and Y181C	58%
16	85a	0.027	K103N + Y181C, F227L + V106A	48%
17	91a	0.006	K103N, E138K, L100I, and Y181C	50%
18	97c	0.007	K103N and Y181C	95%
19	101a	2.7	K103N, E138K and Y181C	82%
20	108a	3.05	K103N, Y181C, E138K	59%
21	116c	6.79	L100I, K103N, Y181C, Y188L, E138K, F227L + V106A	52%
22	121b	27.7	L100I, K103N, Y181C, E138K, F227L/V106A	71.2%
23	126a	7.28	K103N, Y181C, Y188L, E138K, and RES056	65.4%
24	132b	0.0058	L100I, K103N, Y181C, E138K and F227L/V106A	71–76%
25	137c	2.70	L100I and K103N + Y181C	83.8%
26	146c	3.4	E138K and K103N	16.5%
27	152b	0.06	K103N and Y181C	32%
28	154a	4.29	K103N + Y181C	59%
29	165a	9.67	L100I, K103N, Y181C, E138K and Y188L	59%
30	172c	1.52	L100I, K103N, Y181C, Y188L, E138K	50%
31	178b	0.14	K103N, Y181C, and E138K	42%
32	180a	6.6	K103N, L1001, and Y181C	78%
33	185a	0.0086	L100I, K103N, Y181C, Y188L, E138K	24.3%
34	191c	0.021	L100I, K103N, Y181C, Y188L, E138K and F227L/V106A	62%
35	198b	2.98	Scaffold for further development	75%
36	202a	6.65	Scaffold for further development	57%
37	207	1.9	F227L + V106A	87%

## 3. Consolidated summary of the potent compounds

## 4. Future perspectives

Anti-HIV drug resistance is one of the serious problems in the world. To solve this problem, novel anti-HIV drugs with enhanced activity and better pharmacokinetics are needed. This review serves as a milestone for chemists to design anti-HIV drugs that will help to eliminate the current problems of anti-HIV drug resistance. The focus of future research studies should be to use the potent compounds reported in the review as leads (1) to design new pyrimidine-based NNRTIs showing potency against new mutations of HIV-1 by adopting integrated computational modelling, high-throughput screening, and innovative delivery systems and (2) to identify novel scaffolds using high-throughput screening technologies in order to speed up the discovery process for resistant strains. In the future, the rational design of NNRTIs using integrated computational modelling and optimization through machine learning is likely to play a key role.

## 5. Conclusion

By assessing the literature from 2018 to 2026, this review concluded that pyrimidine-based NNRTIs, as potent antiviral drugs, are important in fighting HIV-1 strains. Pyrimidine scaffolds have made it possible to develop drugs that can be used against both WT and resistant HIV-1 strains. However, due to the emergence of new mutations, the virus continues to show resistance against drugs. This review presents 37 synthesized pyrimidine-based compounds, out of which 31, 52c, 56c, 66a, 72b, 82a, 85a, 132b, 185a and 191c can play an important role in developing new NNRTIs against HIV with higher potency against K103N, Y181C, and L100I and those HIV-1 strains that show resistance to drugs. Among all of them, only 66a is more potent than etravirine, while 66a, 56c, 72b, 85a, 132b and 185a are more potent than doravirine, but none is more potent than rilpivirine. This review summarizes the various scaffolds that were investigated for HIV-1 NNRTIs during the last decade and identifies some cores that are especially promising. The NNRTIs and diarylpyrimidines are most notable for their conformational flexibility, hydrogen bonding, and potential for substitutions that increase potency and that are tolerated in the face of resistance. The SARs observed over the scaffolds are recurring: halogenation at certain positions is generally beneficial for binding affinity; cyanovinyl and heteroaryl groups are consistently potent, whereas flexible linkers enable the molecule to adjust into the hydrophobic pocket of reverse transcriptase. Important, several series show measurable activity against clinically relevant resistant mutants, such as K103N, Y181C and L100I. No scaffold has proven to be completely resistant to multiresistant strains, but diarylpyrimidines and other analogues are the most consistently resistant scaffolds and may be the next generation of NNRTIs.

## Conflicts of interest

There are no conflicts to declare.

## Abbreviations

RTIs: Reverse transcriptase inhibitors
NNRTIs: Non-nucleoside reverse transcriptase inhibitors
NRTIs: Nucleoside reverse transcriptase inhibitors
HIV: Human immunodeficiency virus
HIV-1: Human immunodeficiency virus type-1
HIV-2: Human immunodeficiency virus type-2
Boc: *Tert*-butoxycarbonyl
r.t.: Room temperature
SARs: Structure–activity relationships
HOAc: Acetic acid
NIS: *N*-Iodosuccinimide
TFA: Trifluoroacetic acid
DMF: Dimethylformamide
DCM: Dichloromethane
CN: Cyanide
CV: Cyanovinyl
BINAP: 2,2′-bis(diphenylphosphino)-1,1′-binaphthyl
EtOH: Ethanol
Ar: Aromatic ring
TEA: Triethylamine
IC_50_: 50% Inhibition concentration
EC_50_: 50% effective concentration
DAPY: Diarylpyrimidine
*m*-CPBA: *Meta*-chloroperoxybenzoic acid
THF: Tetrahydrofuran
WT: Wild-type
Boc_2_O: Di-*tert*butyl dicarbonate
HATU: Hexafluorophosphate azabenzotriazole tetramethyl uranium

## Data Availability

No primary research results, software or code have been included and no new data were generated or analyzed as part of this review.
